# Bioinspired Synthesis of Platensimycin from Natural *ent*-Kaurenoic Acids

**DOI:** 10.1021/acs.orglett.3c01470

**Published:** 2023-06-20

**Authors:** Álvaro Pérez, José F. Quílez del Moral, Alberto Galisteo, Juan M. Amaro, Alejandro F. Barrero

**Affiliations:** † Department of Organic Chemistry, Institute of Biotechnology, University of Granada, 18071 Granada, Spain; ‡ Department of Chemistry, Faculty of Sciences, University of Los Andes, Merida 5101, Venezuela

## Abstract

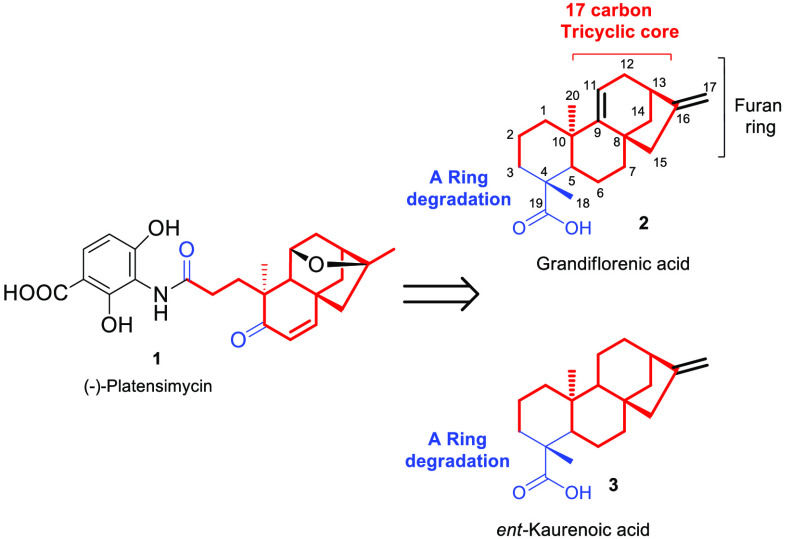

The biomimetic formal synthesis of the antibiotic platensimycin for the treatment of infection by multidrug-resistant bacteria was accomplished starting from either *ent*-kaurenoic acid or grandiflorenic acid, each of which is a natural compound available in multigram scale from its natural source. Apart from the natural origin of the selected precursors, the keys of the described approach are the long-distance functionalization of *ent*-kaurenoic acid at C11 and the efficient protocol for the A-ring degradation of the diterpene framework.

The proliferation of antibiotic-resistant bacteria and fungi is proving to be one of the major health issues in developed countries. According to estimates from the Centers for Disease Control and Prevention, >2.8 million people are infected by antibiotic-resistant pathogens each year in the United Sates, resulting in at least 35 000 deaths, a figure that is very similar to that for Europe.^1^ In this context, the search for new antibiotics presenting new modes of action to fight against resistant bacteria is becoming a pressing global need. In 2006, (−)-platensimycin (PTM), a meroditerpenoid from *Streptomyces platensis*, was isolated by Merck researchers.^2,3^ Its activity has been tested against Gram-positive bacteria, including methicillin-resistant *Staphylococcus aureus* (MRSA), vancomycin-intermediate *S. aureus* (VISA), and vancomycin-resistant enterococci (VRE) among others.^4,5^ Additionally, this substance possesses a unique mode of action as a suppressor of β-ketoacyl-(acyl-carrier protein) synthase II (FabF) engaged in the mechanism of fatty acid biosynthesis.^2,3^ This unique mode of action, its challenging structural and functional architecture, has attracted the attention of many synthetic chemists, and consequently, a number of synthetic strategies have been developed to complete the synthesis of PTM and analogues.^6−16^ Furthermore, a fermentation process for producing PTM from *S. platensis* SB12026 was described.^17^
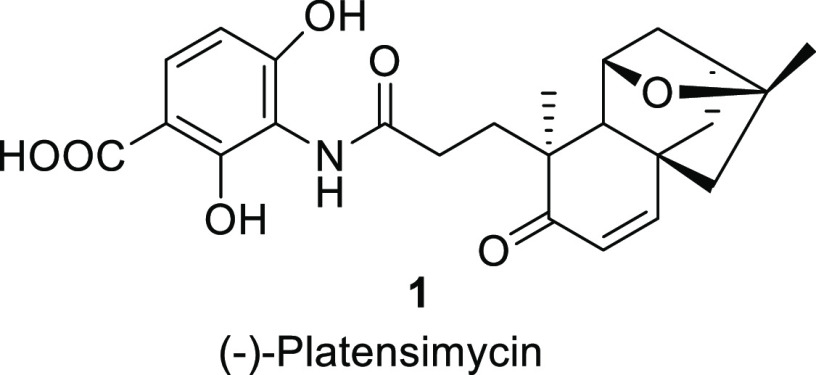


Recent studies have proven that *ent*-kauran-16α-ol and other *ent*-kauranes^18−22^ are involved as intermediates in the PTM tricyclic core biosynthesis. On the basis of this biosynthetic evidence, it was anticipated that natural *ent*-kaurenoic and grandiflorenic acids contain the structural and stereochemical requirements to eventually afford PTM. Indeed, both compounds include in their structure not only the C17 tricyclic carbon framework of PTM but also the appropriate stereochemistry at C8–C10 and C13. Furthermore, the exocyclic methylene at C16 (together with the C9=C11 bond in the case of grandiflorenic acid) should enable generation of the tetrahydrofuran moiety. Finally, the presence of the carboxylic acid at C4 will be key to the development of a new A-ring degradation protocol that should involve the loss of C4, C18, and C19 (Figure 1).

**Figure 1:**
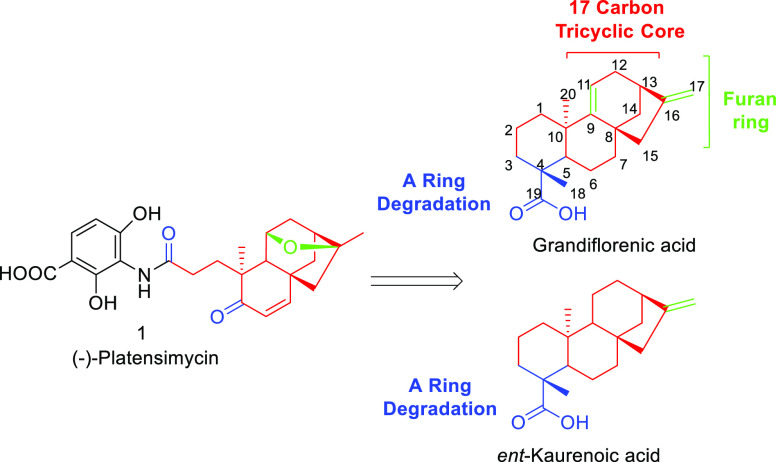
Evaluation of the structural features of grandiflorenic and *ent*-kaurenoic acids enabling the synthesis of PTM.

The use of these starting materials will depend on their availability and easy accessibility in multigram quantities. Because both *ent*-kaurenoic (**3**) and grandiflorenic (**2**) acids are commercially available from Sigma Aldrich with expensive prices ranging from 301 € for grandiflorenic acid to 342 € for *ent*-kaurenoic acid (prices per milligram), the search for suitable natural sources of these compounds constitutes a determining factor for the success of this strategy. In this regard, plants such as *Helianthus annuus* (sunflower) and *Stevia lucida* were reported to possess good concentrations of kaurenoic acid (sunflower)^23,24^ and *ent*-kaurenoic and grandiflorenic acids (*S. lucida*).^25^ In addition, because the worldwide production of sunflower oil in 2022 exceeded 57 million tons,^26^ the use of the residue of sunflower heads would constitute a nice example of turning agricultural residues into ecological and economic assets.^27^

The retrosynthetic planning of our work is shown in Scheme 1. The first disconnection, that is, the formation of an amide bond from platensic acid (**4**) to obtain PTM (**1**), was described by Nicolaou and co-workers in 2009.^16^ The synthesis of acid **4** was proposed to occur via the oxidative degradation of the olefin present on the A-ring of **5**, which, in turn, will be obtained by oxidative decarboxylation of **6**. The generation of **6** from grandiflorenic acid would involve a double regio- and stereoselective hydration to generate the 11,17-diol formation, which after dehydration of the primary alcohol and subsequent acid cyclization would produce the ether bridge present in **6**. Moreover, the methylene anti-Markownikoff hydration of kaurenoic acid would enable us to obtain **6** through a long-distance functionalization mediated by lead tetraacetate (LTA). Grandiflorenic acid (**2**) and *ent*-kaurenoic acid (**3**) will be obtained from the aerial parts from *S. lucida*., and *H. annuus* heads, respectively.

**Scheme 1:**
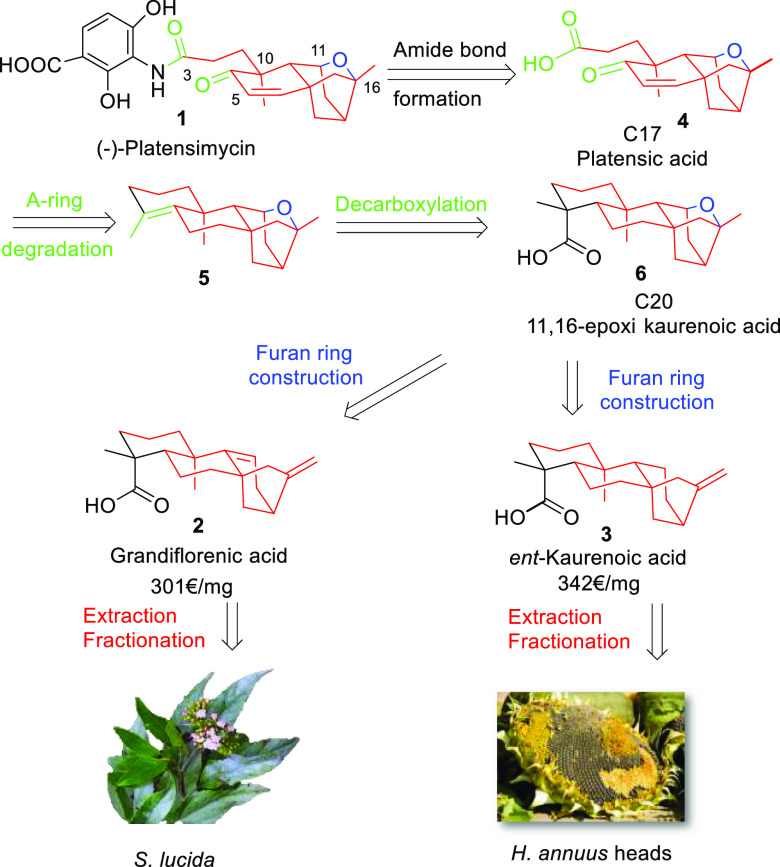
Retrosynthetic Analysis of PTM

As mentioned above, the availability of starting materials from inexpensive and accessible natural sources represents a crucial feature of our strategy. For this reason, we started by developing an appropriate procedure for the extraction of *ent*-kaurenoic acid from sunflower heads (Barrero et al., 2023, unpublished results). On the contrary, acids from aerial parts of *S. lucida* were obtained via Soxhlet extraction and fractionation following Amaro’s procedure.^25^

Once we had multigram quantities of our starting materials in hand, we decided to study the key step of A-ring degradation using the product of hydrogenation of *ent*-kaurenoic acid (**7**) as a simple model. Thus, the synthetic procedure started with the reduction of *ent*-kaurenoic acid by catalytic hydrogenation to afford **7** in 75% yield (Scheme 2).^28^ Oxidative decarboxylation of **7** with lead tetraacetate^29,30^ afforded a variable mixture of acetylated compound **8** and regioisomers **9a**–**c**. Treatment of this mixture with *p*-toluenesulfonic acid caused both acetic acid elimination and olefin isomerization to afford only the desired tetrasubstituted olefin **9c** in 91% yield in two steps. A-Ring opening was accomplished by bubbling ozone through a solution of **9c** in dichloromethane at 0 °C to furnish diketone **10** in 82% yield. The intramolecular aldolic condensation of **10** provided pentacyclic methyl ketone **11** (Scheme 2). It is noteworthy that exposure of **11** to ozone caused the opening of the cyclopentene ring to afford, after esterification, ketoester **12** in 85% yield. As required, two carbon atoms were lost during the process, which should involve degradation of the initially formed α-diketone. A proposed mechanism for rationalizing this transformation is shown in the Supporting Information. Finally, α,β-unsaturated ketone **13** was obtained via treating **12** with phenyl selenium chloride in EtOAc and subsequent elimination of the corresponding selenoxide generated after the addition of H_2_O_2_ [83% yield (Scheme 2)].

**Scheme 2:**
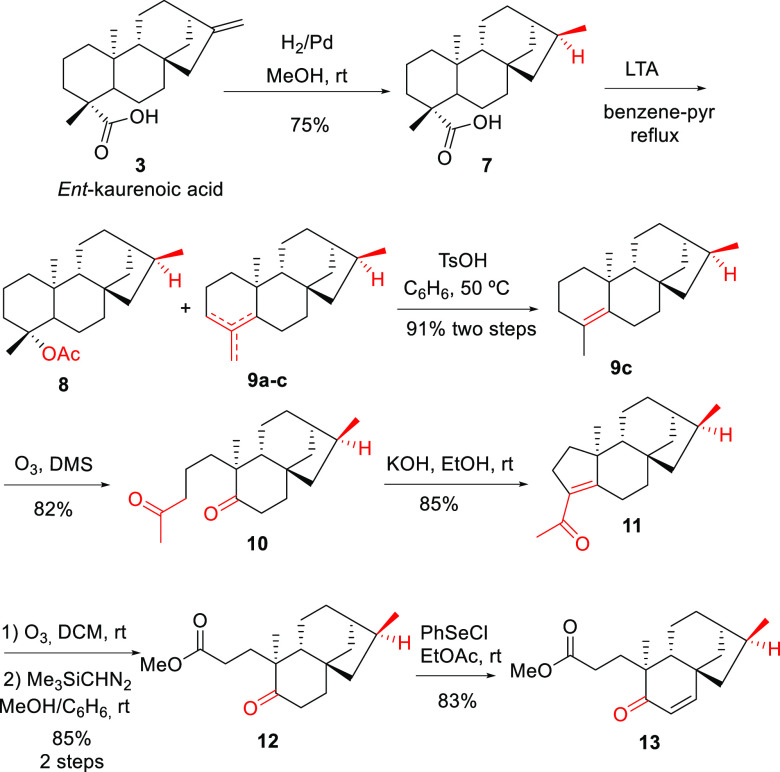
*ent*-Kaurenoic Acid A-Ring Degradation Model

Once the appropriate experimental conditions for the A-ring degradation were found, we focused our efforts on developing a strategy for the construction of the furan ring in PTM using the methyl esters of *ent*-kaurenoic and grandiflorenic acids as starting materials (see Schemes 4 and 5). The two parallel approaches starting from each one of these precursors converged in the generation of furan derivative **6** (Scheme 3).

**Scheme 3:**
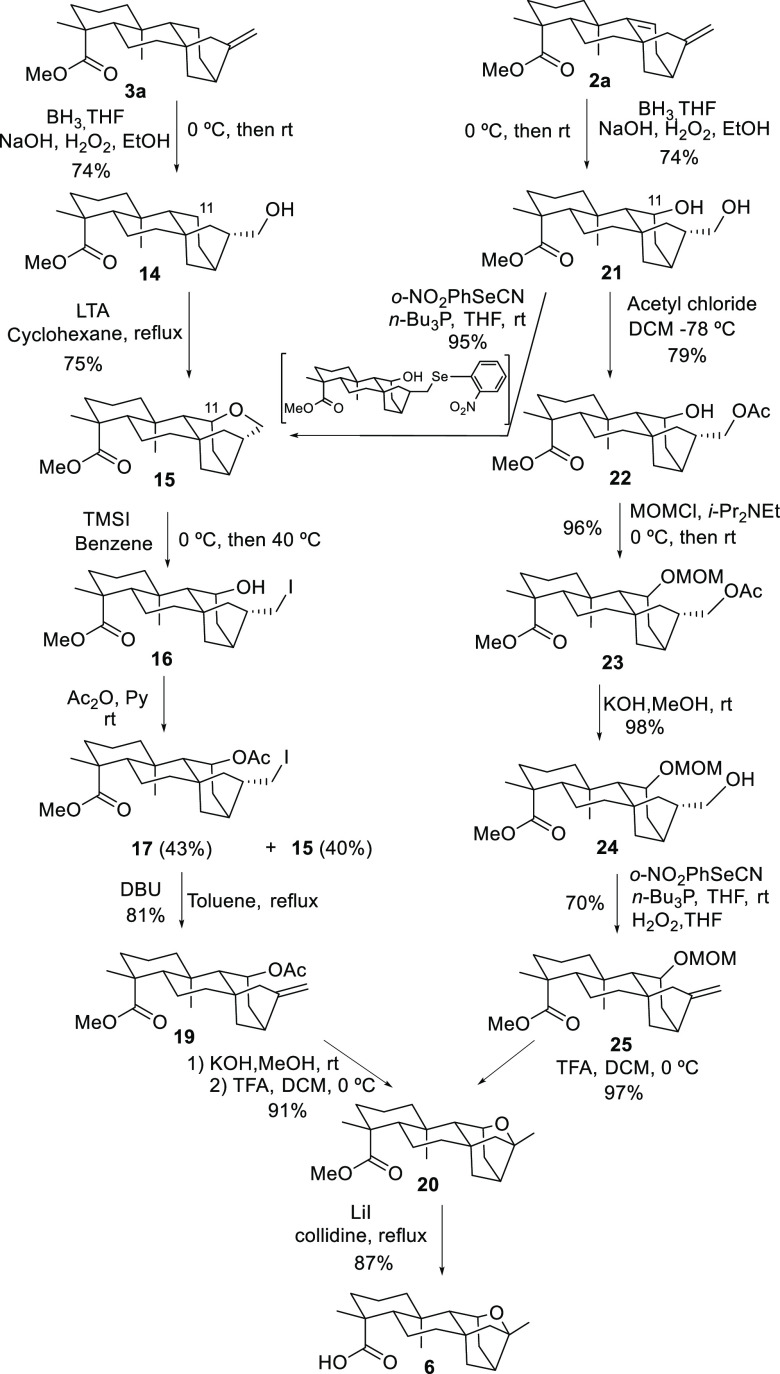
Ether Ring Synthesis

Starting from the methyl ester of *ent*-kaurenoic acid (**3a**), our first approach to PTM began with the regio- and stereoselective hydration by the most accessible α face of the olefin present in **3a** using BH_3_ in THF as a hydroborating agent and H_2_O_2_/KOH as an oxidant. Primary alcohol **14** was thus obtained in 74% yield. The required oxygenation at C11 was accomplished by treating **14** with LTA in refluxing cyclohexane^31−33^ to produce **15** in a remarkable 75% yield, improving the result reported by McAlles and McCrindle^32^ for this transformation. This good yield is the result of the spatial proximity of C17-hydroxyl to H11β.

Trimethylsilyl iodide-mediated opening of cyclic ether **15** produced, after acetylation of the resulting unstable iodo alcohol **16**, primary iodide **17** and starting **15** in 40% and 43% yields, respectively, in a one-pot reaction. Additionally, we obtained a minor product (**18**) in 13% yield with a double bond at C11, as a result of the secondary hydroxyl group elimination. It should be noted that the presence of **15** derives from cyclization of the intermediate iodo alcohol and not from an unaltered starting material. Iodine elimination of **17** with DBU produced exocyclic olefin **19** in 81% yield, along with minor proportions of cyclic ether **15** (6% yield). Hydrolysis of acetate **19**(34) and subsequent treatment of the corresponding alcohol with TFA caused the desired regioselective cyclization to generate targeted furan derivative **20** in a combined 91% yield. Finally, treatment of **20** with lithium iodide in collidine at reflux afforded acid **6**(35) in 87% yield (Scheme 3).

The route from grandiflorenic acid started with the double regio- and selective hydroboration of the olefinic bonds of methyl gradiflorenate (**2a**) to give diol **21**. The selectivity of this double hydration process is noteworthy, especially if we considered that the diol obtained after a stepwise hydroboration is **21b**, the C11 epimer of diol **21**. To rationalize this facial selectivity, it is proposed that the BH_3_/THF reagent initially binds selectively by the α face to the exocyclic olefin to produce an alkylborane complex (**I**), which evolves toward cyclic dialkylborane **II** after an intramolecular hydroboration of the C9=C11 bond by the β face (Scheme 4).

**Scheme 4:**
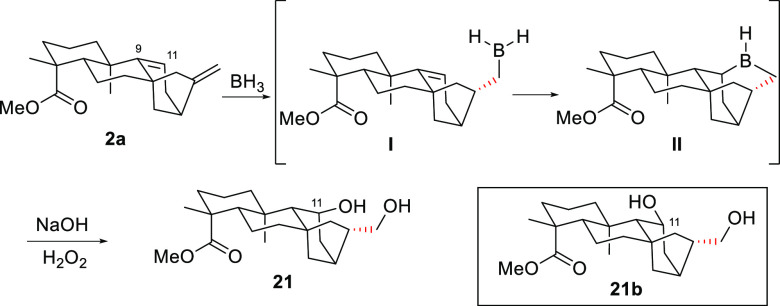
Selective Double Hydroboration–Oxidation of **6**

Once we obtained diol **21**, selective dehydration of the primary alcohol was tried using Grieco’s dehydration procedure.^36^ Via this process, pyran derivative **15** was again formed, most likely due to the spatial proximity of the hydroxyl groups, which tends to collapse toward the stable six-membered heterocyclic ring via a SN_2_ displacement of the corresponding seleno intermediate. Although at this point cyclic ether **15** is an intermediate in the kaurenic acid approach to furan derivative **6**, we geared our efforts toward establishing an alternative route to target **6**.

Our new approach from diol **21** involved some protecting group manipulation. Thus, selective acetylation of the primary alcohol of **21** was accomplished using acetyl chloride at −78 °C to afford acetate **22** in 79% yield. The secondary alcohol was then protected with methoxymethyl chloride (MOMCl) to give **23** in 96% yield. Alkaline deprotection of the acetate group led to primary alcohol **24**, which was now successfully eliminated following Grieco’s protocol^33^ to furnish olefin **25** (69% yield, two steps). Finally, furan derivative **20** was produced in 97% yield by treating **25** with TFA, after a one-pot deprotection–cyclization process.

Once we succeeded in making our two approaches converge in tetrahydrofuran derivative **6**, our next goal was to apply to **6** the A-ring degradation experimental conditions that were optimized with the reduced derivative of kaurenoic acid (**7**) (Scheme 2). Following the procedure detailed above, the decarboxylation of carboxylic acid **6** was conducted using lead tetraacetate. The resulting reaction mixture was then reacted with *p*TsOH to obtain olefin **5** in low yields. Gratifyingly, when the mentioned crude was treated with molecular iodine, the desired tetrasubstituted **5** was obtained in 78% yield over two steps. Ozonolysis led to A-ring cleavage, affording diketone **26** in 85% yield (Scheme 5). Intramolecular aldol condensation of compound **26** with potassium hydroxide in ethanol provided enone **27** in 69% yield. Ozonolysis of **27** caused, as previously described, the loss of two carbon atoms and the generation of methyl ester **28** after methylation with trimethylsilyl diazomethane of the corresponding acid (63% yield, two steps). Finally, the dehydrogenation of **28** to produce methyl platensinoate (**4a**) was accomplished in 83% yield using the same PhSeCl/H_2_O_2_ protocol previously described in the model study.

**Scheme 5:**
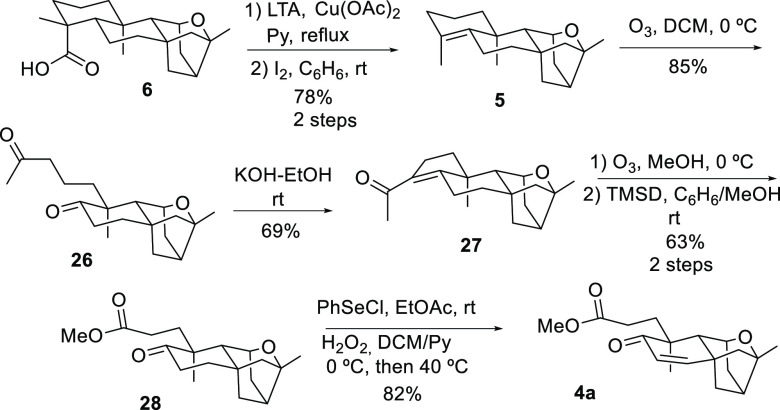
Synthesis of Methyl Platensinoate (**4a**)

Because compound **4a** constitutes an intermediate in the PTM synthesis described by Nicolaou in 2009,^16^ the herein described synthetic approach constitutes a formal enantioselective synthesis of PTM. It should be noted that the spectroscopic data of our methyl platensinoate (**4a**) match completely those described by Nicolau et al. for the same product.

In conclusion, we have investigated two approaches to **4a**, resulting in the formal bioinspired synthesis of PTM, a multidrug-resistant antibiotic possessing a unique mode of action as a suppressor of fatty acid biosynthesis in bacteria. The use of renewable starting materials was essential in our strategies. The use of *ent*-kaurenoic acid present in sunflowers, whose oil worldwide production in 2020 exceeded 55 million tons, features the harnessing of agricultural residues to produce value-added chemicals. Also key in our strategies was the unprecedented protocol described for the A-ring degradation of kaurene diterpenoids, which additionally enabled the required loss of three carbon atoms for the synthesis of methyl platensinoate. Additionally, the long-distance functionalization at C11 in the first approach, as well as the double hydroboration–oxidation in the second, also stands out as a key step. All in all, our synthetic route competes favorably with other synthetic approaches, resulting in fact in one of the shortest sequences to this natural product.^37^ Finally, our protocol is flexible enough to be applicable to the synthesis of other PTM analogues in the search for more active antibiotics.

## Data Availability

The data underlying this study are available in the published article and its Supporting Information.

## Supporting Information Available

Copies of ^1^H and ^13^C{^1^H} NMR and two-dimensional spectra of the obtained compounds (PDF)
FAIR data, including the primary NMR FID files, for compounds **2**–**15** and **17**–**28** (ZIP)
