# Supplementary material for: Bioinspired Synthesis of Platensimycin from Natural ent-Kaurenoic Acids
Source: Org Lett. 2023 Jun 20;25(29):5401–5. doi: 10.1021/acs.orglett.3c01470 (PMC10391625; doi:10.1021/acs.orglett.3c01470)

Bioinspired Synthesis of Platensimycin from Natural *ent*-Kaurenoic acids.

Álvaro Pérez†, José F.Quílez del Moral†*, Alberto Galisteo†, Juan M. Amaro§ and Alejandro F. Barrero†*

†Department of Organic Chemistry, Institute of Biotechnology, University of Granada, 18071 Granada, Spain.

§ Department of Chemistry, Faculty of Sciences, University of Los Andes, Merida C.P. 5101, Venezuela.

* jfquilez@ugr.es; afbarre@ugr.es

Table of Contents

Material and methods, general procedure and spectral data S-2

References S-23

^{1}H and ^{13}C NMR Spectra S-26

Materials and Methods

All air- and water-sensitive reactions were performed in flaks flame-dried under a positive flow of argon and conducted under an argon atmosphere. The solvents used were purified according to standard literature techniques and stored under argon. Anhydrous dichloromethane was distilled from calcium hydride (5% w/v) under positive pressure of nitrogen. THF, Cyclohexane, Benzene and Toluene were freshly distilled immediately prior to use from sodium/benzophenone and strictly deoxygenated for 30 min under argon. Reagents were purchased at the higher commercial quality and used without further purification, unless otherwise stated. Silica gel SDS 60 (35−70 µm) was used for flash column chromatography. Reactions were monitored by thin-layer chromatography carried out on 0.25 mm E. Merck silica gel plates (60F-254) using UV light as the visualizing agent and solutions of phosphomolybdic acid in ethanol. Ozonizations were carried out with ECO-DE Ozonator plus with an O_3 stream (500mg/h, 0,17 mmol/min). NMR spectra were recorded with BRUKER Avance NEO (^{1}H NMR 500 MHz/^{13}C NMR 125 MHz), and BRUKER Avance NEO (^{1}H NMR 400 MHz/^{13}C NMR 100 MHz), spectrometers. High-resolution mass spectra (HRMS) were determined on an Autospec-Q VG-Analytical (FISONS) mass spectrometer, equipped with a quadrupole-time-of-flight (Q-TOF) mass analyzer. Two-dimensional (correlated spectroscopy, heteronuclear single-quantum correlation spectroscopy, heteronuclear multiple bond correlation, nuclear Overhauser enhancement spectroscopy) NMR spectroscopy was used where appropriate to assist the assignment of signals in the ^{1}H and ^{13}C NMR spectra. Structural assignments were made with additional information from gCOSY, gHSQC, and gHMBC experiments.

Plant material and isolation of starting material.

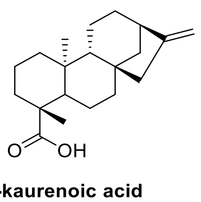

ent-kaurenoic acid

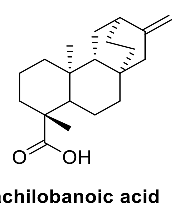

trachilobanoic acid

Helianthus annuus were collected in Villalobos, Zamora, Spain in July 2019. Sunflower heads (25 kg), without seeds, were extracted by maceration in MTBE, two times 15 minutes each. The resultant extract (25 g) was treated with different bases to give a 4:1 mixture (11974 mg) of *ent*-

kaurenoic acid and trachilobanoic acid. Ratios were determined by integration of the ^{1}H spectrum of the mixture. Spectroscopic data match with those reported on bibliography. [1] The detailed procedure has been recently submitted to be patented.

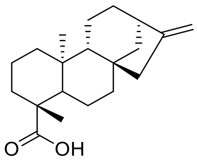
ent-Kaurenoic acid

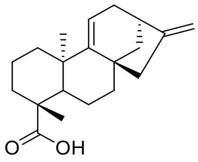
Grandiflorenic acid

Stevia lucida was collected in Páramo de la Negra, close to El Delgadito (Merida, Venezuela) in March 2019. Dried aerial parts (22 Kg) were milled to powder and extracted in a Soxhlet using EtOH. The resultant extract (5657 g) was redissolved on MeOH, adsorbed on silica gel and extracted with hexane. The solvent was evaporated in vacuo and concentrate (682 g), then solved in hot methanol (MeOH) and refrigerated 12 hours at 4°C. The mixture was centrifugated and evaporated the supernatant, obtaining 613 g of extract which was absorbed on celite and purified in a silica gel chromatography column using increasing gradients of ethyl acetate (EtOAc) in hexane (H). *Ent*-kaurenoic acid (**3**) (6277 mg) and grandiflorenic acid (**2**) (5940 mg) were separated on silica gel/$AgNO_3$ column. Spectroscopic data match with those reported on bibliography.[2]

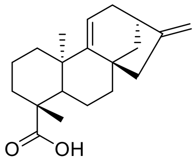
Grandiflorenic acid

*Grandiflorenic acid (**2**).* White crystals. ^{1}H NMR (400 MHz, $CDCl_3$) δ 5.26 (t, *J* = 3.5 Hz, 1H), 4.94 (s, 1H), 4.82 (s, 1H), 2.79 (s, 1H), 2.63 (d, *J* = 15.6, 2.4 Hz, 1H), 2.54 – 2.40 (m, 2H), 2.25 – 2.15 (m, 2H), 2.05 – 1.83 (m, 5H), 1.70 – 1.60 (m, 2H), 1.56 – 1.44 (m, 3H), 1.33 – 1.29 (m, 1H), 1.27 (s, 3H), 1.05 (s, 3H), 1.04 – 0.99 (m, 1H). ^{13}C NMR (101 MHz, $CDCl_3$) δ 183.1 (C), 158.6 (C), 155.9 (C), 114.9 (CH), 105.5 (CH_2), 50.3 (CH_2), 46.6 (CH), 44.9 (CH_2), 44.7 (C), 42.3 (C), 41.2 (CH), 40.8 (CH_2), 38.8 (C), 38.3 (CH_2), 37.9 (CH_2), 29.7 (CH_2), 28.2 (CH_3), 23.6 (CH_3), 20.2 (CH_2), 18.5 (CH_2). HRMS (ESI-QTOF) m/z: $[M + H]^+$ calcd for $C_{20}H_{29}O_2$ 301.2168, found 301.2169.

Synthesis of methyl grandiflorenate (2a).

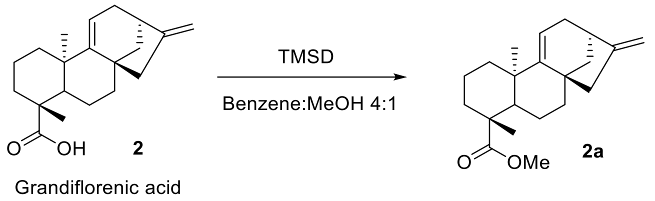

To a solution of grandiflorenic acid (2958 mg, 9.85 mmol) in 60 mL of benzene/MeOH (4:1), $TMSCHN_2$ (1340 mg, 11,8 mmol) was added. After consumption of starting material, the solvent was removed under reduced pressure to give methyl ester **2a** (2774 mg, 90% yield). Spectroscopic data match with those reported on bibliography.[3,7]

Synthesis of *ent*-kaur-16-en-18-oic acid, methyl ester (3a).

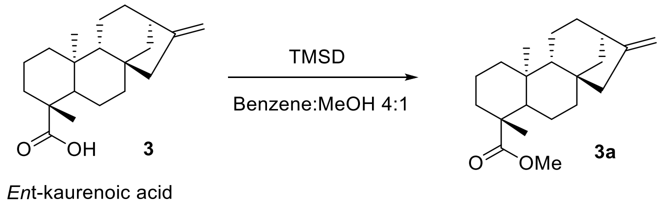

To a solution of *ent*-kaurenoic acid (**3**) (5000 mg, 16.53 mmol) in 100 mL of a 4:1 mixture of benzene/MeOH, $TMSCHN_2$ (2266 mg, 20 mmol) was added. The mixture was stirred at room temperature conditions for 15 minutes. After consumption of starting material, the solvent was removed under reduced pressure to give **3a** (4813mg, 92%). Spectroscopic data match with those reported on bibliography.[1]

*Kaur-16-en-18-oic acid, methyl ester (**3a**).* White solid. ^{1}H NMR (500 MHz, $CDCl_3$) δ 4.82 (s, 1H), 4.76 (s, 1H), 3.66 (s, 3H), 2.65 (s, 1H), 2.19 (d, J = 13.2, 2.8 Hz, 1H), 2.09 – 2.05 (m, 2H), 1.99 (dd, J = 11.4, 2.3 Hz, 1H), 1.91 – 1.74 (m, 4H), 1.67 – 1.42 (m, 7H), 1.19 (s, 3H), 1.15 (dd, J = 11.3, 5.0 Hz, 1H), 1.09 – 1.03 (m, 2H), 1.02 (d, J = 4.4 Hz, 1H), 0.85 (s, 3H), 0.82 (d, J = 4.3 Hz, 1H). ^{13}C NMR (126 MHz, $CDCl_3$) δ 178.1 (C), 155.9 (C), 103.0 (CH_2), 57.1 (CH), 55.1 (CH), 51.1 (CH_3), 49.0 (CH_2), 44.2 (C), 43.9 (CH), 43.8 (C), 41.3 (CH_2), 40.8 (CH_2), 39.7 (CH_2), 39.4 (C), 38.1 (CH_2), 33.1

(CH_2), 28.8 (CH_3), 21.9 (CH_2), 19.2 (CH_2), 18.4 (CH_2), 15.4 (CH_3). HRMS (ESI-QTOF) m/z: $[M + H]^+$ calcd for $C_{21}H_{33}O_2$ 317.2481, found 317.2466.

Hydrogenation of *ent*-kaurenoic acid to give *ent*-kauran-19-oic acid (7).

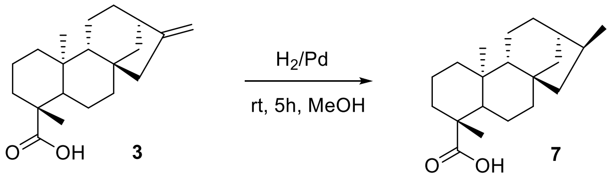

To a stirred solution of *ent*-kaurenoic acid (**3**) (204 mg, 0.68 mmol) in dry methanol (9.5 mL) was added a catalytic amount of palladium in activated carbon. The flask was tightly closed and sealed. The mixture was stirred for 7h under a hydrogen balloon pressure. After that, the reaction mixture was diluted with MTBE (50 mL) and filtered through celite. The resultant crude was dried with anhydrous Na_2SO_4 and concentrated under reduced pressure. Purification by flash chromatography with H/MTBE, 4:1 provided 152 mg (75% yield) of a 3:1 mixture of epimers where **7** was the major one. The spectroscopic data of compound **7** match with those reported in the literature.[3,4]

*Ent-kauran-19-oic acid (**7**).* $[\alpha]_D$ -32.1 (c 1, DCM). White solid. ^1H NMR (500 MHz, $CDCl_3$) δ 2.17 (bd, *J* = 12.3 Hz, 1H), 2.05 (h, 1H), 1.99 (d, *J* = 11.3 Hz, 1H), 1.95 – 1.75 (m, 5H), 1.64 (t, *J* = 12.5 Hz, 1H), 1.60 – 1.38 (m, 7H), 1.25 (s, 3H), 1.10 – 1.00 (m, 3H), 1.02 (d, *J* = 7.1 Hz, 3H), 0.97 (s, 3H), 0.97 – 0.78 (m, 3H). ^{13}C NMR (126 MHz, $CDCl_3$) δ 183.3 (C), 57.1 (CH), 56.6 (CH), 48.8 (CH_2), 44.8 (C), 43.7 (C), 42.20 (CH_2), 40.8 (CH_2), 40.8 (CH_2), 40.1 (CH), 39.7 (C), 37.9 (CH_2), 34.4 (CH), 29.0 (CH_3), 25.9 (CH_2), 22.1 (CH_2), 19.1 (CH_2), 19.0 (CH_2), 15.8 (CH_3), 15.5 (CH_3). HRMS (ESI-QTOF) m/z: $[M + H]^+$ calcd for $C_{20}H_{33}O_2$ 305.2481, found 305.2467.

Decarboxylation of 7 to give compound 8 and the mixture of isomers 9a-c.

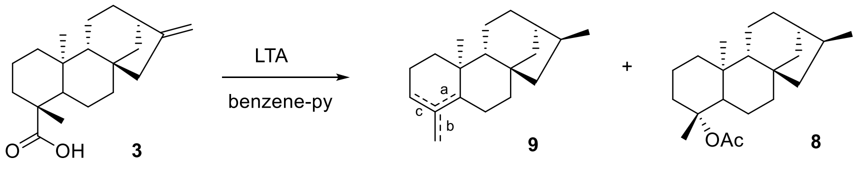

To a dry, flamed and argon-filled flask containing 200 mg (0.66 mmol) of stirred **7** in anhydrous bencene (2 mL), 0.2 mL of distilled pyridine (Py) was added followed by $Cu(OAc)_2$ (5 mg, 0.028 mmol) and lead tetracetate (LTA) (368 mg, 0.83 mmol)[5]. The mixture was refluxed for 2 h (oil bath) and then cooled to room temperature. The resulting mixture was diluted with MTBE (50 mL) and filtered through a short plug of silica gel, which was washed with ethyl acetate. The mixture was washed with 2N HCl, saturated $NaHCO_3$ and brine, and dried with anhydrous Na_2SO_4. The solvent was removed, giving a residue, which was flash chromatographed (H/MTBE, 9:1) to give 70 mg (33% yield) of a mixture of epimers where **8** was the major one, and the mixture of isomers **9a-c** in a 1.4:1:1 proportion (103 mg, 60% yield). Ratios were determined by integration of the ^1H spectrum of the mixture.

*Compound **8***. White solid. $[\alpha]_D$ -43.6 (c 1.0, DCM). ^1H NMR (500 MHz, $CDCl_3$) δ 2.58 (d, *J* = 11.0 Hz, 1H), 2.02 (h, 1H), 1.95 (s, 1H), 1.93 (s, 3H), 1.92 – 1.88 (m, 1H), 1.79 – 1.42 (m, 13H), 1.41 (s, 3H), 1.32 (td, *J* = 12.1, 4.3 Hz, 1H), 1.06 (d, *J* = 7.0 Hz, 1H), 1.00 (d, *J* = 2.0 Hz, 3H), 0.99 (s, 3H), 0.98 – 0.91 (m, 2H), 0.85 – 0.78 (m, 1H); ^{13}C {^1H} NMR (126 MHz, $CDCl_3$) δ 170.4 (C), 86.3 (C), 57.3 (CH), 54.7 (CH), 48.8 (CH_2), 44.6 (C), 41.3 (CH_2), 40.8 (CH_2), 40.1 (CH), 40.0 (C), 39.2 (CH_2), 37.4 (CH_2), 34.3 (CH), 25.9 (CH_2), 22.9 (CH_3), 19.9 (CH_2), 19.2 (CH_2), 19.0 (CH_3), 18.7 (CH_2), 17.5 (CH_3), 15.8 (CH_3).

Synthesis of 9c.

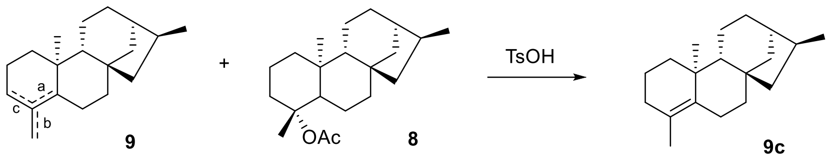

To a solution of the previous crude (227 mg) in 10 mL of benzene was added *p*-toluenesulfonic acid (TsOH) (30 mg, 0.18 mmol) under argon. The reaction was heated at 50ºC for 2.5 h (oil bath), and then diluted with 100mL of MTBE. The resulting mixture as washed with saturated Na_2CO_3 and brine, and dried with anhydrous Na_2SO_4. The solvent was removed under reduced pressure obtaining 202 mg of crude which was purified by flash chromatography with H/MTBE, 9:1 to afford 220 mg (97% yield) of a mixture of epimers where **9c** was the major one.

*Compound **9c**.* Colorless oil. $[\alpha]_D$ -80.1 (c 1.0, DCM). ^1H NMR (500 MHz, $CDCl_3$) δ 2.47 (dt, *J* = 13.6, 3.4 Hz, 1H), 2.11 (d, *J* = 11.9 Hz, 1H), 2.05 (h, *J* = 12.7, 6.6 Hz, 1H), 1.96 – 1.78 (m, 6H), 1.61 (s, 3H), 1.61 – 1.50 (m, 6H), 1.49 – 1.33 (m, 3H), 1.12 (s, 3H), 1.09 – 1.04 (m, 2H), 1.02 (d, *J* = 7.1 Hz, 3H), 0.90 (td, *J* = 6.5, 1.7 Hz, 1H); ^{13}C {^1H} NMR (126 MHz, $CDCl_3$) δ 137.2 (C), 123.7 (C), 56.2 (CH), 48.3 (CH_2), 45.0 (C), 41.2 (CH_2), 40.4 (CH_2), 40.1 (CH), 39.7 (CH_2), 39.4 (C), 34.6 (CH), 33.0 (CH_2), 26.0 (CH_2), 24.4 (CH_2), 22.8 (CH_3), 19.8 (CH_2), 19.6 (CH_3), 18.9 (CH_2), 15.9 (CH_3). HRMS (ESI-QTOF) m/z: $[M - H]^+$ calcd for $C_{19}H_{29}$ 257.2269, found 257.2266.

Ozonolysis of 9c.

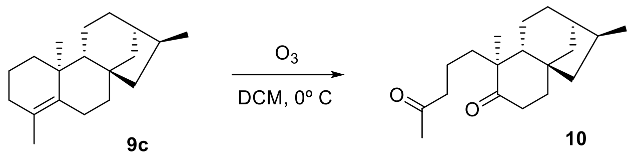

A mixture of epimers where **9c** was the major component (44 mg, 0,17 mmol) was solved in dichloromethane (DCM) (11 mL) and Py (0.06mL) in a two-neck flask at 0 ºC of temperature. Then, an O_3 stream (500mg/h, 0,17 mmol/min) was bubbled in the stirring mixture[6]. After 1 hour, 3 mL of dimethyl sulfide was added and the resulting mixture was diluted with 50 mL of DCM. The organic layer was washed with brine and dried over Na_2SO_4. The crude was purified via flash chromatography (5:1 H/MTBE), to give 40 mg (82% yield) of a mixture of epimers where **10** was the major one.

*Compound **10**.* Colorless oil. $[\alpha]_D$ -94.9 (c 1, DCM). ^1H NMR (500 MHz, $CDCl_3$) δ 2.61 (td, *J* = 14.8, 5.9 Hz, 1H), 2.50 – 2.30 (m, 2H), 2.26 (ddd, *J* = 15.1, 4.4, 2.8 Hz, 1H), 2.13 (s, 3H), 2.10 (h, *J* = 12.7, 6.6 Hz, 1H), 2.07 – 2.01 (m, 2H), 1.84 – 1.59 (m, 6H), 1.52 – 1.43 (m, 2H), 1.34 – 1.25 (m, 3H), 1.23 (dd, *J* = 11.6, 5.6 Hz, 2H), 1.18 (s, 3H), 1.05 (d, *J* = 7.1 Hz, 3H), 1.04 – 1.03 (m, 1H). ^{13}C NMR (126 MHz, $CDCl_3$) δ 216.3 (C), 209.2 (C), 52.5 (C), 47.2 (CH_2), 46.6 (CH), 44.3 (CH_2), 43.9 (C), 39.8 (CH), 39.7 (CH_2), 37.4 (CH_2), 37.3 (CH_2), 37.2 (CH_2), 34.6 (CH), 29.9 (CH_3), 25.4 (CH_2), 24.4 (CH_3), 20.0 (CH_2), 19.5 (CH_2), 15.7 (CH_3). HRMS (ESI-QTOF) m/z: $[M + H]^+$ calcd for $C_{19}H_{31}O_2$ 291.2324, found 291.2326.

Synthesis of compound 11.

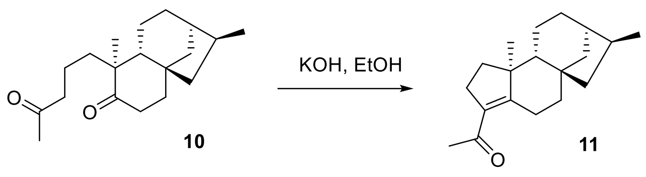

A mixture of epimers where **10** was the major component (65 mg, 0.22 mmol) was solved in EtOH (5mL). Potassium hydroxide (KOH) (840 mg, 15 mmol) was added, turning the reaction from white to yellow color. After the consumption of the starting material, the reaction was diluted in MTBE, washed with brine and dried over Na_2SO_4. The crude was concentrated and purified by flash chromatography (H/MTBE, 9:1) to obtain getting 51 mg (85% yield) of a mixture of epimers where **11** was the major one.

Compound ***11****.* Colorless syrup. $[\alpha]_D$ -74.5 (c 1, DCM). ^{1}H NMR (500 MHz, $CDCl_3$) δ 3.29 (dt, *J* = 14.2, 3.4 Hz, 1H), 2.65 – 2.57 (m, 1H), 2.53 (ddt, *J* = 15.5, 9.5, 1.5 Hz, 1H), 2.23 (s, 3H), 2.14 – 2.05 (m, 1H), 2.02 (d, *J* = 11.5 Hz, 1H), 1.98 (q, *J* = 4.9, 4.3 Hz, 1H), 1.85 – 1.79 (m, 1H), 1.74 (q, *J* = 13.9, 7.2 Hz, 1H), 1.68 (t, 1H), 1.65 – 1.59 (m, 1H), 1.58 – 1.24 (m, 6H), 1.14 (s, 3H), 1.10 (ddt, *J* = 11.4, 4.1, 1.8 Hz, 1H), 1.03 (d, *J* = 7.1 Hz, 3H), 0.93 – 0.86 (m, 1H). ^{13}C NMR (126 MHz, $CDCl_3$) δ 199.4 (C), 163.7 (C), 131.5 (C), 55.33 (CH), 53.7 (C), 47.2 (CH_2), 44.3 (C), 40.8 (CH_2), 39.9 (CH), 39.5 (CH_2), 39.5 (CH_2), 34.4 (CH), 30.8 (CH_2), 30.6 (CH_3), 25.4 (CH_2), 23.5 (CH_2), 21.0 (CH_3), 20.8 (CH_2), 15.7 (CH_3). HRMS (ESI-QTOF) m/z: $[M + H]^+$ calcd for $C_{19}H_{29}O$ 273.2218 , found 273.2223.

Synthesis of 12.

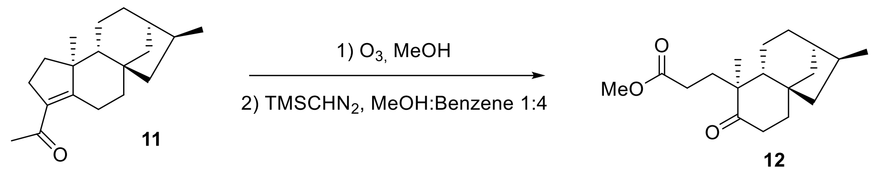

A O_3 stream (500 mg/h, 0.17 mmol/min) was bubbled into a solution of mixture of epimers where **11** was the major component (83 mg, 0.30 mmol) in MeOH (5 mL) for 45 min at 0 °C. The solvent was completely removed and 2.5 mL of a 4:1 mixture of benzene/MeOH was added to the residue. To this solution, 0.16 mL (0.34 mmol) of trimethylsilyldiazomethane ($TMSCHN_2$) was added. The mixture was stirred for 15 min at room temperature and then, the solvent was

concentrated under reduced pressure and the crude was purified via flash chromatography using H:MTBE 6:1 as eluent to furnish 75 mg (85% yield, 2 steps) of a mixture of epimers where **12** was the major one.

*Compound **12***. Colorless syrup. $[\alpha]_D$ -15.2 (c 1, DCM). ^{1}H NMR (500 MHz, $CDCl_3$) δ 3.65 (s, 3H), 2.63 (td, *J* = 14.6, 5.9 Hz, 1H), 2.30 – 1.87 (m, 7H), 1.80 – 1.46 (m, 9H), 1.26 – 1.23 (m, 1H), 1.23 (s, 3H), 1.05 (d, *J* = 7.1 Hz, 3H), 1.03 – 0.99 (m, 1H). ^{13}C NMR (126 MHz, $CDCl_3$) δ 215.77 (C), 174.26 (C), 51.88 (C), 51.54 (CH_3), 47.31 (CH), 47.26 (CH_2), 43.89 (C), 39.67 (CH_2), 39.67 (CH), 37.56 (CH_2), 37.15 (CH_2), 34.49 (CH), 32.36 (CH_2), 29.97 (CH_2), 25.37 (CH_2), 23.61 (CH_3), 20.05 (CH_2), 15.68 (CH_3).. HRMS (ESI-QTOF) m/z: $[M + H]^+$ calcd for $C_{18}H_{29}O_3$ 293.2117, found 293.2121.

Scheme 1. *Ent*-kaurenoic ring A degradation. Mechanistic proposal

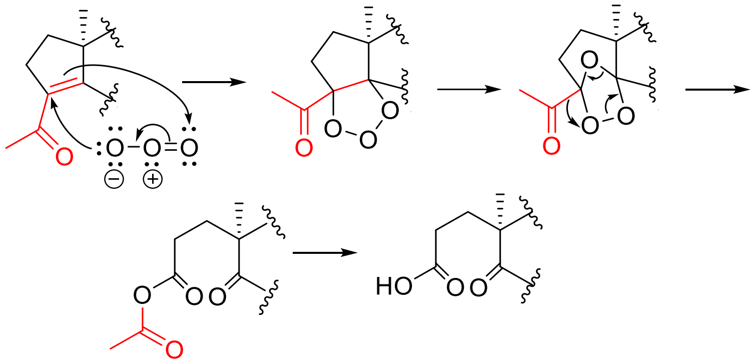

Synthesis of 13.

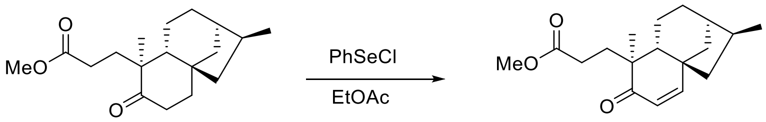

12 **13**

To a solution of a mixture of epimers where **12** was the major component (23 mg, 0.08 mmol) in 2.5 mL of EtOAc, PHSeCl (24 mg, 0.13 mmol) was added. The mixture was stirred for 22 h at room temperature under argon until the consumption of starting material. The solvent was then removed. The resulting crude was dissolved in 0.01 mL of pyridine and 2.5 mL of DCM. Finally, 0.01 mL of H_2O_2 was added at 0 ºC. The 0 ºC bath was then removed and the mixture refluxed

for 10 min. The mixture was purified via flash chromatography using H:EtOAc 6:1 as eluent to obtain 20 mg (83% yield) of a mixture of epimers where **13** was the major one.

Compound ***13***. Colorless syrup. [α]D -7.4 (c 1, DCM). ^{1}H NMR (500 MHz, $CDCl_3$) δ 6.60 (d, *J* = 10.0 Hz, 1H), 5.80 (d, *J* = 10.0 Hz, 1H), 3.58 (s, 3H), 2.19 – 1.95 (m, 5H), 1.80 (m, 2H), 1.73 – 1.55 (m, 4H), 1.44 – 1.37 (m, 2H), 1.19 (s, 1H), 1.16 – 1.14 (m, 1H), 1.13 (s, 3H), 1.02 (d, *J* = 7.1 Hz, 3H). ^{13}C NMR (126 MHz, $CDCl_3$) δ 204.4 (C), 174.0 (C), 159.0 (CH), 126.7 (CH), 51.6 (CH_3), 48.7 (C), 48.4 (CH_2), 45.6 (C), 43.0 (CH), 40.9 (CH_2), 39.3 (CH), 35.1 (CH), 32.1 (CH_2), 29.7 (CH_2), 25.3 (CH_2), 23.8 (CH_3), 18.4 (CH_2), 15.6 (CH_3). HRMS (ESI-QTOF) m/z: $[M + H]^+$ calcd for $C_{18}H_{27}O_3$ 291.1960, found 291.1954.

Synthesis of 14.

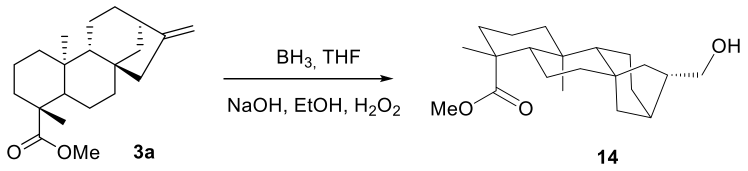

To a solution of 2600 mg (8.22 mmol) of **3a** in dry THF (100 mL), 2.3 mL of a 1M borane-THF solution was added dropwise at 0 ºC under inert gas (Ar). After 15 min, the 0 ºC bath was removed and the mixture was stirred for 1 h until consumption of the starting material. Then, the reaction was cooled (0 ºC) and EtOH (32 mL), 4N NaOH (22.5 mL) and 29 mL of H_2O_2 were added. The resulting mixture was stirred for 25 min and then diluted with 150 mL of EtOAc, washed with water and brine and dried over Na_2SO_4. The solvent was evaporated over reduced pressure and the crude was chromatographed via silica gel column (H/MTBE 5:1) to afford **14** (1524 mg, 74% yield). The spectroscopic data of compound **14** match with those reported in the literature[8,13,14].

*Compound **14**.* White foam. [α]D -60.2 (c 1, DCM). ^{1}H NMR (500 MHz, $CDCl_3$) δ 3.64 (dd, *J* = 7.3, 6.0 Hz, 2H), 3.57 (s, 3H), 2.16 – 2.06 (m, 3H), 1.92 (d, *J* = 11.1 Hz, 1H), 1.83 – 1.72 (m, 3H), 1.64 (qd, *J* = 13.6, 3.0 Hz, 1H), 1.54 – 1.32 (m, 8H), 1.09 (s, 3H), 1.01 – 0.88 (m, 5H), 0.75 (s, 3H), 0.70 (dd, *J* = 13.8, 4.1 Hz, 1H). ^{13}C NMR (126 MHz, $CDCl_3$) δ 178.16 (C), 64.29 (CH_2), 56.99 (CH), 56.42 (CH), 51.13 (CH_3), 44.25 (C), 43.82 (C), 43.62 (CH_2), 43.23 (CH), 42.09 (CH_2), 40.76 (CH_2),

40.36 (CH_2), 39.43 (C), 38.10 (CH_2), 36.94 (CH), 28.73 (CH_3), 26.02 (CH_2), 22.22 (CH_2), 19.16 (CH_2), 19.12 (CH_2), 15.36 (CH_3). HRMS (ESI-QTOF) m/z: $[M + H]^+$ calcd for $C_{21}H_{35}O_3$ 335.2586, found 335.2591.

Synthesis of 15.

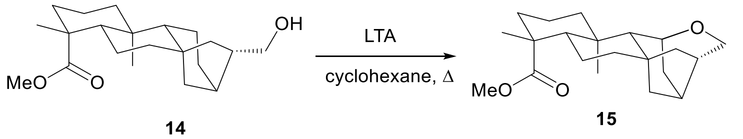

14 15

To a solution of **14** (730 mg, 2.18 mmol) in dry cyclohexane (137 mL), LTA (6580 mg, 14.84 mmol) was added. The reaction was heated (oil bath) to reflux under inert atmosphere.[15] After 24 h, the reaction was cooled to room temperature and passed through a small column of celite/silica gel (1:1) that was washed with MTBE. The solvent was evaporated under reduced pressure and the resultant crude was purified via column chromatography (H/MTBE 7:1) to afford **15** (542 mg, 75%).

*Compound **15**.* White solid. $[\alpha]_D$ -26.0 (c 1, DCM). ^1H NMR (500 MHz, $CDCl_3$) δ 3.94 (bs, 1H), 3.77 (d, *J* = 10.0 Hz, 1H), 3.54 (s, 3H), 3.44 (dd, *J* = 10.0, 1.8 Hz, 1H), 2.25 – 1.97 (m, 4H), 1.84 (dt, *J* = 13.4, 3.8 Hz, 1H), 1.80 – 1.48 (m, 5H), 1.45 – 1.32 (m, 5H), 1.19 (bs, 1H), 1.09 (s, 3H), 1.09 – 1.04 (m, 1H), 1.03 – 0.88 (m, 3H), 0.66 (s, 3H). ^{13}C NMR (126 MHz, $CDCl_3$) δ 178.2 (C), 69.1 (CH), 64.7 (CH_2), 62.9 (CH), 57.3 (CH), 51.2 (C), 48.8 (CH_2), 43.80 (C), 42.5 (CH_2), 42.2 (C), 40.7 (CH_2), 39.8 (CH_2), 38.0 (CH_2), 37.7 (C), 35.6 (CH_3), 32.5 (CH), 31.2 (CH_2), 29.0 (CH_3), 22.3 (CH_2), 19.2 (CH_2), 16.1 (CH_3). HRMS (ESI-QTOF) m/z: $[M + H]^+$ calcd for $C_{21}H_{33}O_3$ 333.2430, found 333.2432.

Synthesis of 17.

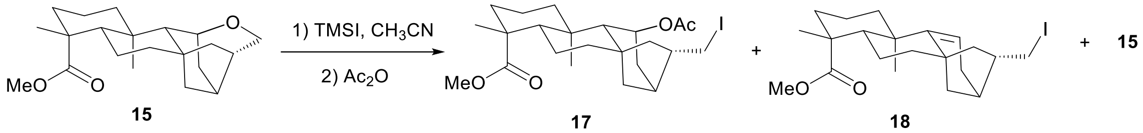

15 17 18

To a solution of **15** (71 mg, 0.21 mmol) in 5 mL of dry acetonitrile, trimethylsilyl iodide (ITMS) (0.03 mL, 0.25 mmol) was added dropwise at 0 ^oC under argon. After 15 minutes the ice bath was removed and the solution heated to 40 ^oC (oil bath) for 45 min. The mixture was then cooled

to room temperature and Ac_2O (0.36 mL) was added. After stirring for 10 min, the mixture was cooled to 0 ^{O}C, diluted with 50 mL of MTBE and washed with 2N HCl, saturated $NaCO_3$ and brine. The organic layer was dried over Na_2SO_4 and the solvent removed under reduced pressure. The crude was purified via flash chromatography in (H/MTBE (10:1)) to afford **17** (39 mg, 40% yield), **18** (12 mg, 13% yield), and **15** (31 mg, 43% yield).

*Compound **17***. White solid. $[\alpha]_D$ -10.3 (c 1, DCM). ^{1}H NMR (500 MHz, $CDCl_3$) δ 5.14 (d, *J* = 6.4 Hz, 1H), 3.66 (s, 3H), 3.42 (dt, *J* = 47.1, 9.0 Hz, 2H), 2.47 – 2.36 (m, 1H), 2.28 (q, 1H), 2.19 (dt, 1H), 2.07 (s, 3H), 2.02 (d, *J* = 11.7 Hz, 2H), 1.88 – 1.74 (m, 3H), 1.66 – 1.60 (m, 2H), 1.23 – 1.21 (m, 1H), 1.19 (s, 3H), 1.09 – 1.05 (m, 1H), 1.02 (td, 1H), 0.79 (s, 3H). ^{13}C NMR (126 MHz, $CDCl_3$) δ 177.8 (C), 169.2 (C), 68.3 (CH), 63.3 (CH), 56.7 (CH), 51.2 (CH_3), 45.0 (CH_2), 44.2 (C), 43.8 (C), 43.8 (CH), 41.9 (CH_2), 40.2 (CH_2), 39.7 (CH_2), 39.1 (CH), 38.5 (C), 37.9 (CH_2), 32.2 (CH_2), 28.8 (CH_3), 21.7 (CH_3), 21.7 (CH_2), 18.9 (CH_2), 15.2 (CH_3), 9.2 (CH_2). HRMS (ESI-QTOF) m/z: $[M + H]^+$ calcd for $C_{23}H_{36}O_4I$ 503.1658, found 503.1650.

*Compound **18***. Pale oil. $[\alpha]_D$ -101.1 (c 1, DCM). ^{1}H NMR (400 MHz, $CDCl_3$) δ 5.94 (ddt, *J* = 9.5, 6.3, 1.5 Hz, 1H), 5.69 (dd, *J* = 9.8, 3.8 Hz, 1H), 3.65 (s, 3H), 3.23 – 3.11 (m, 2H), 2.65 – 2.44 (m, 2H), 2.19 (dt, 1H), 2.01 (d, *J* = 11.0 Hz, 1H), 1.89 – 1.78 (m, 3H), 1.74 – 1.61 (m, 3H), 1.53 – 1.41 (m, 3H), 1.20 (s, 3H), 1.15 – 1.01 (m, 4H), 0.93 (ddd, *J* = 13.6, 7.6, 1.8 Hz, 1H), 0.74 (s, 3H). ^{13}C NMR (101 MHz, $CDCl_3$) δ 177.87 (C), 130.39 (CH), 128.44 (CH), 62.12 (CH), 55.87 (CH), 51.17 (CH_3), 50.48 (CH), 48.95 (CH_2), 44.09 (C), 43.88 (C), 41.69 (CH), 41.40 (CH_2), 39.87 (CH_2), 38.86 (C), 37.99 (CH_2), 37.38 (CH_2), 28.71 (CH_3), 21.77 (CH_2), 18.9 (CH_2)9, 15.27 (CH_3), 11.04 (CH_2). HRMS (ESI-QTOF) m/z: $[M + H]^+$ calcd for $C_{21}H_{32}O_2I$ 443.1447, found 443.1441.

Synthesis of 19.

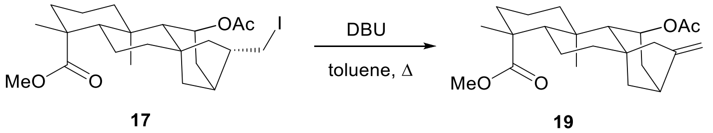

17 19

To a solution of **17** (198 mg, 0.39 mmol) in anhydrous toluene (5 mL), 0.19 mL (1.3 mmol) of 1,8-diazabicyclo[5.4.0]undec-7-ene (DBU) was added. The mixture was heated to reflux (oil bath) under inert atmosphere for 19 h, and then column chromatographed using H/MTBE (3:1) as eluent. Compound **19** (119 mg, 81% yield) and ether **15** (8 mg, 6% yield) were obtained.

*Compound **19**.* Colourless oil. ^{1}H NMR (500 MHz, $CDCl_3$) δ 5.05 (d, *J* = 5.2, 1.5 Hz, 1H), 4.84 (s, 1H), 4.70 (s, 1H), 3.66 (s, 3H), 2.64 (dt, *J* = 3.8 Hz, 1H), 2.50 (d, *J* = 15.1 Hz, 1H), 2.20 (dt, *J* = 9.7 Hz, 1H), 2.08 – 1.96 (m, 2H), 1.95 (s, 3H), 1.91 – 1.63 (m, 6H), 1.62 – 1.44 (m, 3H), 1.32 (s, 1H), 1.20 (s, 3H), 1.19 – 1.17 (m, 1H), 1.11 (dd, *J* = 12.1, 2.3 Hz, 1H), 1.07 – 0.90 (m, 2H), 0.80 (s, 3H). ^{13}C NMR (126 MHz, $CDCl_3$) δ 177.85 (C), 170.06 (C), 155.17 (C), 103.11 (CH_2), 69.35 (CH), 60.83 (CH), 56.88 (CH), 51.24 (CH_3), 47.80 (CH_2), 43.85 (C), 43.04 (C), 42.23 (CH), 41.13 (CH_2), 40.24 (CH_2), 39.50 (CH_2), 39.15 (CH_2), 38.32 (C), 37.96 (CH_2), 28.81 (CH_3), 21.70 (CH_3), 21.68 (CH_2), 19.02 (CH_2), 15.29 (CH_3). $[\alpha]_D$ -76.3. HRMS (ESI-QTOF) m/z: $[M + H]^+$ calcd for $C_{23}H_{35}O_4$ 375.2535, found 375.2531.

Synthesis of 20.

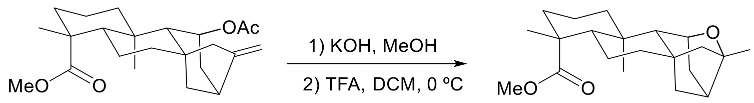

19 **20**

A solution of **19** (119 mg, 0.32 mmol) in 14 mL of 10% KOH/MeOH was stirred at room temperature for 19 h. After this time, the solvent was evaporated under reduced pressure and the crude was dissolved in MTBE (50 mL), washed with NH_4Cl, water and brine and dried over Na_2SO_4. The resulting crude after evaporation of the solvent was dissolved in DCM (14 mL) and cooled to 0 ^oC. After that, trifluoroacetic acid (TFA) (1,13 mL, 14.7 mmol) was added and the mixture was stirred at 0 ^oC for 45 min under argon. Then, the solution was diluted with MTBE (50 mL), washed with Na_2CO_3 and brine and dried over Na_2SO_4. Finally, the organic layer was concentrated under reduced pressure and the crude was purified via flash chromatography (H/MTBE 2:1). Ether **20** (96 mg, 91% yield) was obtained in two steps. The spectroscopic data of compound **20** match with those reported in the literature.[1,11]

*Compound **20**.* Colorless oil. $[\alpha]_D$ -56.8 (c 1, DCM). ^{1}H NMR (600 MHz, $CDCl_3$) δ 4.28 (bt, *J* = 3.5 Hz, 1H), 3.57 (s, 3H), 2.14 (t, *J* = 6.5 Hz, 1H), 2.12 – 2.10 (m, 1H), 1.93 (d, *J* = 11.2 Hz, 1H), 1.87 (dd, *J* = 11.7, 3.6 Hz, 1H), 1.84 – 1.66 (m, 4H), 1.61 – 1.53 (m, 1H), 1.45 (d, *J* = 3.6 Hz, 1H), 1.44 (s, 1H), 1.39 – 1.33 (m, 3H), 1.27 (s, 3H), 1.27 (s, 1H), 1.19 – 1.15 (m, 1H), 1.10 (s, 3H), 1.05 – 0.92 (m, 3H), 0.80 (s, 3H). ^{13}C NMR (151 MHz, $CDCl_3$) δ 178.2 (C), 85.7 (C), 76.9 (CH), 58.3 (CH), 57.5 (CH), 56.9 (CH_2), 51.2 (CH_3), 45.5 (CH), 45.0 (C), 43.7 (C), 43.4 (CH_2), 41.4 (CH_2), 40.5 (CH_2), 38.3

(CH_2), 38.0 (CH_2), 36.9 (C), 28.9 (CH_3), 23.2 (CH_3), 21.6 (CH_2), 19.0 (CH_2), 17.4 (CH_3). HRMS (ESI-QTOF) m/z: $[M + H]^+$ calcd for $C_{21}H_{33}O_3$ 333.2430, found 333.2427.

Synthesis of compound 21.

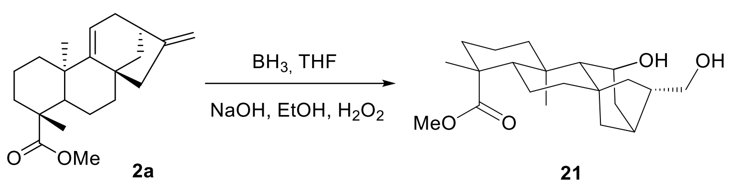

To a solution of 2774 mg (8.82 mmol) of **2a** in dry tetrahydrofuran (THF) (100 mL), 2.5 mL of a 1M borane-THF solution was added dropwise at 0 ºC under inert gas (Ar). After 15 min, the bath was removed, and the mixture was stirred for 1 h until consumption of the starting material. Then, the reaction was cooled (0 ºC) and EtOH (33 mL), NaOH 4N (24 mL) and 30 mL of H_2O_2 were added. The reaction was further stirring for 30 min. The resulting mixture was then diluted with 150 mL of EtOAc, washed with water and brine and dried over Na_2SO_4. The solvent was evaporated over reduced pressure and the crude was chromatographed via silica gel column using H:TBME 1:1 as eluent to afford diol **21** (2287 mg, 74% yield). Spectroscopic data of compound **21** match with those reported in the literature.[8]

*Compound **21***. White solid. $[\alpha]_D$ -28.9 (c 1, DCM). ^{1}H NMR (600 MHz, $CDCl_3$) δ 3.94 – 3.85 (m, 3H), 3.64 (s, 3H), 2.22 – 2.14 (m, 2H), 2.11 (dh, *J* = 11.9, 6.6, 5.9 Hz, 1H), 2.00 – 1.74 (m, 8H), 1.67 (q, *J* = 13.4 Hz, 1H), 1.62 – 1.40 (m, 4H), 1.22 (bs, 1H), 1.18 (s, 3H), 1.12 – 1.00 (m, 3H), 0.71 (s, 3H). ^{13}C NMR (151 MHz, $CDCl_3$) δ 178.0 (C), 72.9 (C), 66.8 (CH), 65.6 (CH), 63.1 (CH_2), 56.8 (CH), 51.2 (CH_3), 43.8 (C), 42.8 (CH), 42.1 (CH_2), 40.2 (CH_2), 40.0 (CH_2), 39.9 (CH_2), 38.4 (C), 38.0 (CH_2), 37.78 (CH), 35.4 (CH_2), 28.7 (CH_3), 21.9 (CH_2), 19.0 (CH_2), 14.9 (CH_3). HRMS (ESI-QTOF) m/z: $[M - OH]^+$ calcd for $C_{21}H_{33}O_3$ 333.2430, found 333.2429.

Synthesis of compound 15.

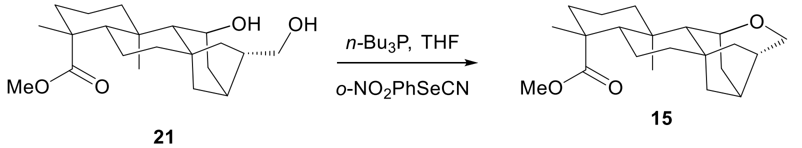

A solution of 2-nitrophenyl selenocyanate (*o*-NO_2PhSeCN) (379 mg, 1.67 mmol) in dry THF (7.5 mL) was added dropwise to a solution of **21** (390 mg, 1.11 mmol) in dry THF (32.5 mL). After that, 0.43 mL (1.67 mmol) of *n*-tributylphosphine (*n*-Bu_3P) was added dropwise. The mixture was stirred at room temperature under argon for 2 h. After this time, the reaction was diluted with MTBE, washed with NH_4Cl, brine and the organic layer dried over Na_2SO_4. The solvent was evaporated in vacuo and the crude was purified by flash chromatography (H/MTBE (1:1)) to obtain pure **15** (341 mg, 95% yield).[9,10]

Synthesis of 22.

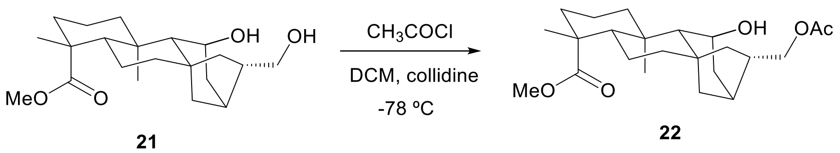

To a solution of **21** (802 mg, 2.29 mmol) at -78 ºC in 76 mL of dry dichloromethane, 1.1 mL of anhydrous collidine (8.47 mmol) and 0.15 mL of acetyl chloride (2.13 mmol) were added dropwise under argon. The mixture was stirred for 20 min. After this time, the mixture was diluted with MTBE and washed with HCl 2N. The aqueous phase was extracted several times with MTBE and the combined organic layer was dried over anhydrous Na_2SO_4 and then, concentrated in vacuo. Column chromatography (H/EtOAc 5:1) provided **22** (712 mg, 79% yield).

*Compound **22**.* White solid. $[\alpha]_D$ -57.3 (c 1, DCM). ^{1}H NMR (500 MHz, $CDCl_3$) δ 4.37 (dd, *J* = 10.8, 8.3 Hz, 1H), 4.23 (dd, *J* = 10.8, 6.6 Hz, 1H), 3.79 (d, *J* = 6.5 Hz, 1H), 3.53 (s, 3H), 2.17 – 2.04 (m, 3H), 1.93 (s, 3H), 1.83 (dt, *J* = 12.5, 3.2 Hz, 1H), 1.78 – 1.70 (m, 5H), 1.66 – 1.32 (m, 7H), 1.08 (s, 3H), 1.06 (s, 1H), 0.98 (dd, *J* = 12.2, 2.3 Hz, 1H), 0.96 – 0.90 (m, 2H), 0.61 (s, 3H). ^{13}C NMR (126 MHz, $CDCl_3$) δ 177.9 (C), 171.4 (C), 66.7 (CH), 66.5 (CH_2), 65.6 (CH), 56.8 (CH), 51.2 (CH_3), 43.8 (C), 42.9 (C), 42.0 (CH_2), 41.5 (CH_2), 40.0 (CH_2), 39.6 (CH_2), 39.5 (CH), 38.3 (C), 38.0 (CH_2), 37.1 (CH), 35.3 (CH_2), 28.7 (CH_3), 21.9 (CH_2), 21.1 (CH_3), 19.0 (CH_2), 14.9 (CH_3). HRMS (ESI-QTOF) m/z: $[M + Na]^+$ calcd for $C_{23}H_{36}O_5Na$ 415.2460, found 415.2453.

Synthesis of 23.

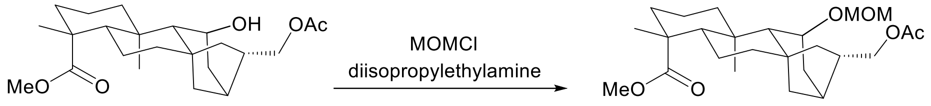

22 **23**

To a solution of **22** (712 mg, 1.81 mmol) in dry DCM (12 mL) at 0 °C, diisopropylethylamine (830 mg, 6.42 mmol) was added under inert atmosphere. After stirring for 5 minutes, chloromethyl methyl ether (MOMCl) (0.45 mL, 5.90 mmol) was added. The reaction mixture was then allowed to warm to room temperature. After stirring for 2h, the reaction was cooled (0 °C) and diluted with MTBE (60 mL). Then, it was washed with 2N HCl, water, $NaHCO_3$ and brine. The organic layer was dried over Na_2SO_4 and concentrated in vacuo. Flash chromatography (H/MTBE 2:1) afforded **23** (763 mg, 96% yield).

*Compound **23**.* Pale syrup. $[\alpha]_D$ -44.5, (c 1, DCM). ^1H NMR (400 MHz, $CDCl_3$) δ 4.52 (s, 2H), 4.34 (dd, J = 10.8, 8.3 Hz, 1H), 4.20 (dd, J = 10.8, 6.4 Hz, 1H), 3.64 (d, J = 6.6 Hz, 1H), 3.56 (s, 3H), 3.27 (s, 3H), 2.17 – 2.06 (m, 3H), 1.96 (s, 3H), 1.92 – 1.71 (m, 5H), 1.63 – 1.32 (m, 7H), 1.19 (s, 1H), 1.10 (s, 3H), 1.04 – 0.86 (m, 4H), 0.65 (s, 3H). ^{13}C NMR (101 MHz, $CDCl_3$) δ 177.9 (C), 171.32(C), 93.40(CH_2), 69.2 (CH), 66.3 (CH_2), 63.4 (CH), 56.9 (CH), 55.4 (CH_3), 51.2 (CH_3), 43.9 (C), 43.1 (C), 42.1 (CH_2), 40.91(CH_2), 40.2 (CH_2), 40.0 (CH_2), 39.7 (CH), 38.2 (C), 38.0 (CH_2), 37.0 (CH), 32.4 (CH_2), 28.8 (CH_3), 22.0 (CH_2), 21.13(CH_3), 19.1 (CH_2), 15.2 (CH_3). HRMS (ESI-QTOF) m/z: $[M + Na]^+$ calcd for $C_{25}H_{40}O_6Na$ 459.2723, found 459.2721.

Synthesis of 24.

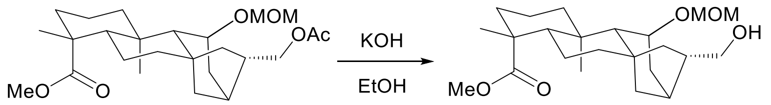

23 **24**

A solution of **23** (763 mg, 1.75 mmol) in 68 mL of 10% KOH/MeOH was stirred at room temperature for 10 min. After this time, the solvent was evaporated under reduced pressure and the crude was dissolved in MTBE (60 mL), washed with NH_4Cl, water and brine and dried over Na_2SO_4. The solvent was evaporated under reduced pressure affording **24** (673 mg, 98% yield).

*Compound **24**.* Pale syrup. [α]$_D$ -36.2, (c 1, DCM). ^{1}H NMR (400 MHz, $CDCl_3$) δ 4.61 (s, 2H), 3.91 (dd, *J* = 10.5, 7.8 Hz, 1H), 3.84 (dd, *J* = 10.7, 6.2 Hz, 1H), 3.74 (d, *J* = 6.8 Hz, 1H), 3.65 (s, 3H), 3.36 (s, 3H), 2.26 – 2.06 (m, 3H), 2.01 – 1.78 (m, 5H), 1.73 – 1.40 (m, 7H), 1.28 (s, 1H), 1.19 (s, 3H), 1.14 – 0.97 (m, 4H), 0.74 (s, 3H). ^{13}C NMR (101 MHz, $CDCl_3$) δ 177.9 (C), 93.4 (CH_2), 69.4 (CH), 64.0 (CH_2) 63.6 (CH), 56.9 (CH), 55.5 (CH_3), 51.2 (CH_3), 43.9 (C), 43.7 (CH), 43.0 (C), 42.2 (CH_2), 40.4 (CH_2), 40.2 (CH_2), 40.2 (CH_2), 38.3 (C), 38.0 (CH_2), 37.1 (CH), 32.5 (CH_2), 28.8 (CH_3), 21.9 (CH_2), 19.1 (CH_2), 15.2 (CH_3). HRMS (ESI-QTOF) m/z: [M + Na]$^+$ calcd for $C_{23}H_{38}O_5Na$ 417.2617, found 417.2619.

Synthesis of 25.

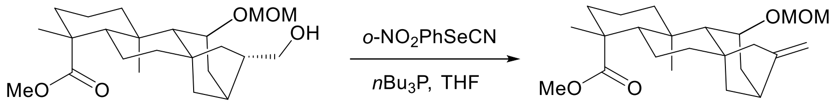

24 **25**

To a solution of **24** (120 mg, 0.21 mmol) in dry THF (10 mL) in a dry flamed flask, a solution of *o*-NO_2PhSeCN (207 mg, 0.912 mmol) in dry THF (5 mL) was added dropwise. After that, 0.23 mL (0.91 mmol) of *n*-Bu_3P were also added. The reaction mixture was stirred at room temperature for 2 h. After this time, the reaction was diluted with MTBE, washed with NH_4Cl and brine and dried over Na_2SO_4. The solvent was evaporated under reduced pressure. The resulting crude was dissolved in dry THF (5.6 mL) at room temperature. Then, 0.16 mL (7.38 mmol) of H_2O_2 was added, and the mixture was heated for 10 minutes. The reaction was then diluted with MTBE 40 mL, washed with distilled water and brine and dried over Na_2SO_4. Flash chromatography (H/MTBE 9:1) afforded **25** (120 mg, 70% yield).

*Compound **25**.* White solid. [α]$_D$ -52.7, (c 1, DCM). ^{1}H NMR (500 MHz, $CDCl_3$) δ 4.77 (s, 1H), 4.56 (s, 1H), 4.52 (d, *J* = 7.0 Hz, 1H), 4.44 (d, *J* = 6.9 Hz, 1H), 3.71 (d, *J* = 5.5 Hz, 1H), 3.57 (s, 3H), 3.25 (s, 3H), 2.53 (dt, *J* = 3.9 Hz, 1H), 2.44 (dd, *J* = 16.7, 2.5 Hz, 1H), 2.16 – 2.10 (m, 1H), 1.97 – 1.34 (m, 11H), 1.30 (s, 1H), 1.12 (s, 3H), 1.06 – 0.91 (m, 4H), 0.67 (s, 3H). ^{13}C NMR (126 MHz, $CDCl_3$) δ 178.0 (C), 155.6 (C), 102.7 (CH_2), 93.6 (CH_2), 70.3 (CH), 61.7 (CH), 57.0 (CH), 55.2 (CH_3), 51.2 (CH_3), 47.4 (CH_2), 43.9 (C), 43.0 (C), 42.7 (CH), 41.4 (CH_2), 40.2 (CH_2), 39.6 (CH_2), 38.2 (CH_2), 38.2 (C), 38.0 (CH_2), 28.8 (CH_3), 21.8 (CH_2), 19.1 (CH_2), 15.2 (CH_3). HRMS (ESI-QTOF) m/z: [M + H]$^+$ calcd for $C_{23}H_{37}O_4$ 377.2692, found 377.2676.

Synthesis of compound 20 from 25.

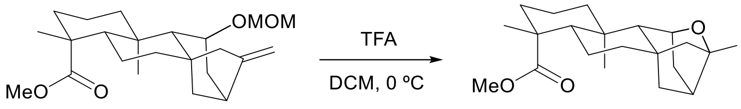

25 **20**

To a cooled (0 ^{o}C) solution of **25** (317 mg, 0.84mmol) in dry DCM, TFA (3.0 mL, 39.18 mmol) was added under argon. The mixture was stirred at 0 ^{o}C for 10 min. Then, the solution was diluted with MTBE (50 mL, washed with Na_2CO_3 and brine and dried over Na_2SO_4. The organic layer was then concentrated under vacuo and the crude was purified via flash chromatography (H/MTBE 2:1) to afford **20** (271 mg, 97% yield).

Synthesis of 6.

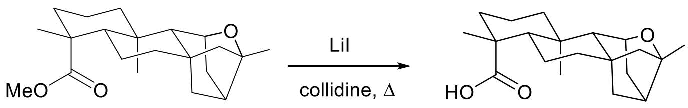

20 **6**

To a solution of **20** (330 mg, 0.99 mmol) in dry collidine (13.9 mL), lithium Iodide (1328 mg, 9.93 mmol) was added. The mixture was heated to reflux (oil bath) under argon for 3 h. The reaction crude was cooled to 0 ^{o}C and quenched with distilled water and MTBE. The mixture was acidulated to pH 3 with 2N HCl and extracted several times with MTBE (70 mL). The combined organic layers were washed with saturated $Na_2S_2O_3$ and dried over Na_2SO_4. The solvent was evaporated over reduced pressure and the resultant crude was purified by flash chromatography (H/EtOAc, (5:1)) to afford **6** (275 mg, 87% yield). The spectroscopic data of compound **6** match with those reported in the literature.[1]

*Compound **6***. Whyte solid. $[\alpha]_D$ -49.8 (c 1, DCM). ^{1}H NMR (500 MHz, $CDCl_3$) δ 4.40 (t, *J* = 3.5 Hz, 1H), 2.27 – 2.18 (m, 2H), 2.04 (d, *J* = 11.3 Hz, 1H), 1.99 (dd, *J* = 11.7, 3.5 Hz, 1H), 1.95 – 1.68 (m, 5H), 1.57 (dd, *J* = 11.2, 3.4 Hz, 1H), 1.54 (bs, 1H), 1.50 – 1.42 (m, 3H), 1.39 – 1.35 (m, 1H), 1.37 (s, 3H), 1.31 – 1.26 (m, 1H), 1.26 (s, 3H), 1.16 – 1.02 (m, 3H), 1.00 (s, 3H). ^{13}C NMR (126 MHz, $CDCl_3$) δ 183.9 (C), 85.8(C), 76.9 (CH), 58.2 (CH), 57.4 (CH), 56.9 (CH_2), 45.5 (CH), 45.0 (C), 43.5 (C), 43.4 (CH_2), 41.3 (CH_2), 40.5 (CH_2), 38.2 (CH_2), 37.7 (CH_2), 37.2 (C), 29.2 (CH_3), 23.2 (CH_3), 21.5 (CH_2), 19.0 (CH_2), 17.7 (CH_3). HRMS (ESI-QTOF) m/z: $[M + H]^+$ calcd for $C_{20}H_{30}O_3$ 319.2273, found 319.2268.

Synthesis of 5.

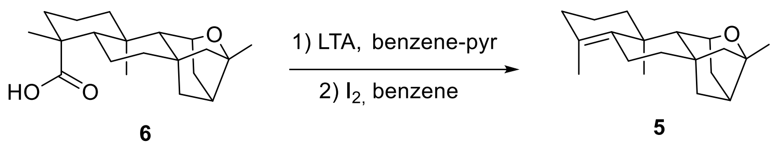

To a dry, flamed and argon-filled flask with 258 mg (0.81 mmol) of **6** in anhydrous benzene (4 mL) was added 0.25 mL of distilled pyridine, $Cu(OAc)_2$ (11 mg, 0.065 mmol) and lead tetracetate (LTA) (934 mg, 2.1 mmol). The mixture was refluxed (oil bath) for 4 h and then cooled to room temperature, diluted in MTBE (50 mL) and filtered through a short plug of silica gel, which was washed with ethyl acetate. The resultant mixture was washed with 2N HCl, saturated $NaHCO_3$ and brine, and dried over anhydrous Na_2SO_4. This reaction crude was treated with I_2 (15 mg, 0.06 mmol) in benzene at room temperature and inert atmosphere (Ar). After stirring for 3 h, the reaction mixture was quenched with MTBE and washed with aqueous 10% $Na_2S_2O_3$, brine and dried over anhydrous Na_2SO_4. The organic layer was concentrated in vacuo and purified by flash column chromatography (H/EtOAc 5:1)) to give **5** (198 mg, 78% yield).

*Compound **5**.* Colorless oil. $[\alpha]_D$ -31.0 (c 1, DCM).^{1}H NMR (500 MHz, $CDCl_3$) δ 4.30 (t, 1H), 2.36 (dt, *J* = 13.4, 3.4 Hz, 1H), 2.17 (t, *J* = 6.5, 1.1 Hz, 1H), 2.03 (dd, *J* = 3.5 Hz, 1H), 2.00 (d, *J* = 11.2 Hz, 1H), 1.96 – 1.74 (m, 4H), 1.70 (dt, 1H), 1.53 (s, 3H), 1.53 (bs, 1H), 1.50 – 1.47 (m, 2H), 1.45 (dd, *J* = 11.1, 3.7 Hz, 1H), 1.29 (s, 3H), 1.36 – 1.20 (m, 5H), 1.11 (d, *J* = 0.8 Hz, 3H). ^{13}C NMR (126 MHz, $CDCl_3$) δ 137.1 (C), 123.8 (C), 85.8 (C), 77.6 (CH), 57.3 (CH), 56.5 (CH_2), 45.4 (CH), 45.2 (C), 42.9 (CH_2), 40.3 (CH_2), 39.9 (CH_2), 37.2 (CH_2), 36.9 (C), 32.7 (CH_2), 24.9 (CH_3), 23.4 (CH_2), 23.3 (CH_3), 19.5 (CH_3), 18.7 (CH_2). HRMS (ESI-QTOF) m/z: $[M + H]^+$ calcd for $C_{19}H_{29}O$ 273.2218, found 273.2216.

Ozonolysis of 5.

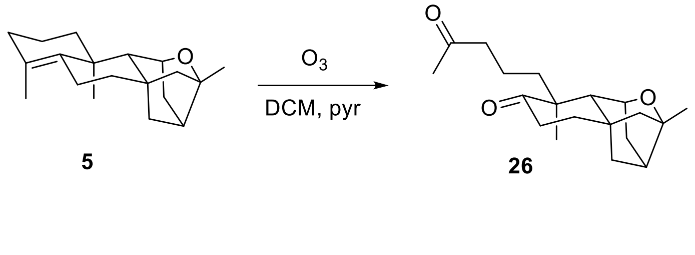

A O_3 stream (500 mg/h, 0.17 mmol/min) was bubbled into a solution of **5** (66 mg, 0,17mmol) in 16 mL of DCM and 0.07 mL of Py at 0 ^{o}C. After stirring for 1 h, 15 mL of dimethyl sulfide were added, and the resulting mixture was diluted in 50 mL of DCM. The reaction crude was washed with brine and dried over Na_2SO_4. The crude was purified via flash chromatography (H/MTBE, 1:4) to give pure **26** (63 mg, 85% yield).

*Compound **26**.* Colorless oil. $[\alpha]_D$ -47.4, (c 1, DCM). ^{1}H NMR (400 MHz, $CDCl_3$) δ 4.32 (t, *J* = 3.5 Hz, 1H), 2.55 (ddd, *J* = 15.1, 13.4, 6.2 Hz, 1H), 2.43 (q, *J* = 7.4 Hz, 2H), 2.36 – 2.26 (m, 2H), 2.16 (s, 1H), 2.14 (s, 3H), 2.05 – 1.93 (m, 3H), 1.87 (td, *J* = 13.6, 5.2 Hz, 1H), 1.67 (td, *J* = 10.8, 10.4, 3.1 Hz, 1H), 1.64 – 1.53 (m, 3H), 1.49 – 1.42 (m, 4H), 1.41 (s, 3H), 1.18 (s, 3H). ^{13}C NMR (101 MHz, $CDCl_3$) δ 215.2 (C), 208.7 (C), 86.3 (C), 77.2 (CH), 55.0 (CH_2), 49.8 (CH), 49.6 (C), 44.8 (CH), 44.4 (C), 44.1 (CH_2), 42.8 (CH_2), 40.7 (CH_2), 38.0 (CH_2), 36.3 (CH_2), 33.5 (CH_2), 29.8 (CH_3), 23.6 (CH_3), 23.1 (CH_3), 19.1 (CH_2). HRMS (ESI-QTOF) m/z: $[M + H]^+$ calcd for $C_{19}H_{29}O_3$ 305.2117, found 305.2110.

Synthesis of compound 27.

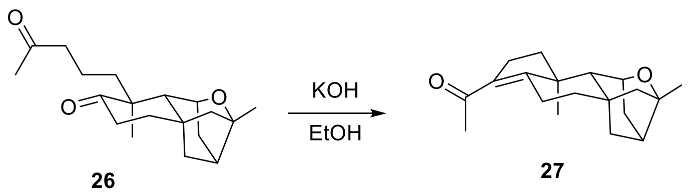

To a solution of **26** (48 mg, 0.16 mmol) in EtOH (5mL), KOH (840mg, 15 mmol) was added. The mixture was stirred for 30 min at room temperature. It was then diluted with MTBE, washed with brine and dried over Na_2SO_4. The crude was concentrated and purified by flash chromatography (H/MTBE, 2:1) to give **27** (31 mg, 69% yield).

*Compound **27**.* Pale syrup. $[\alpha]_D$ -48.7 (c 1, DCM). ^{1}H NMR (500 MHz, $CDCl_3$) δ 4.25 (t, *J* = 3.5 Hz, 1H), 3.33 (dt, *J* = 13.9, 3.5 Hz, 1H), 2.74 – 2.58 (m, 2H), 2.30 (t, *J* = 6.5 Hz, 1H), 2.25 (s, 3H), 2.10 (d, *J* = 11.4 Hz, 1H), 2.05 (dd, *J* = 11.8, 3.4 Hz, 1H), 2.02 – 1.95 (m, 2H), 1.86 (ddd, *J* = 12.5, 7.8, 2.2 Hz, 1H), 1.82 (s, 1H), 1.69 (q, 1H), 1.56 (td, *J* = 10.6, 9.9, 3.7 Hz, 1H), 1.52 – 1.48 (m, 2H), 1.43 (d, *J* = 11.1 Hz, 1H), 1.39 (s, 3H), 1.38 – 1.34 (m, 1H), 1.17 (s, 3H). ^{13}C NMR (126 MHz, $CDCl_3$)

δ 199.5 (C), 163.0 (C), 131.1 (C), 86.0 (C), 77.8 (CH), 57.1 (CH), 55.9 (CH_2), 50.2 (C), 45.2 (CH), 44.9 (C), 42.2 (CH_2), 40.1 (CH_2), 40.0 (CH_2), 36.1 (CH_2), 31.5 (CH_2), 30.6 (CH_3), 23.3 (CH_3), 23.1 (CH_3), 22.6 (CH_2). HRMS (ESI-QTOF) m/z: $[M + H]^+$ calcd for $C_{19}H_{27}O_2$ 287.2011, found 287.2014.

Synthesis of compound 28.

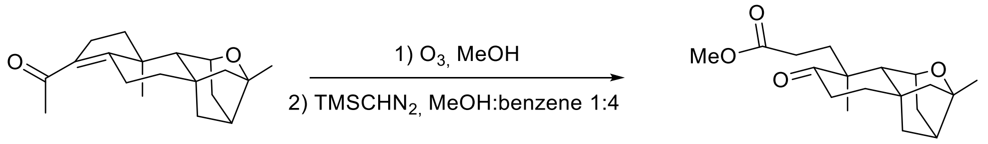

27 28

A O_3 stream (500mg/h, 0,17 mmol/min) was bubbled into a solution of methyl ketone **27** (31 mg, 0.11 mmol) in MeOH (7.2 mL) at 0 ^oC. After 15 min, the reaction was heated to room temperature and stirred for further 45 min. Then, the solvent was evaporated and 2 mL of benzene/MeOH (4:1) was added to the mixture. To this solution, 0.06 mL of trimethylsilyldiazomethane (0.13 mmol) was added and the mixture was stirred for 15 min. Then, the solvent was concentrated under reduced pressure and the crude was purified via flash chromatography (H/MTBE 6:1) to furnish **28** (21 mg, 63% yield) (2 steps).

*Compound **28***. White solid. $[\alpha]_D$ -32.5 (c 1, DCM). ^1H NMR (400 MHz, $CDCl_3$) δ 4.33 (t, *J* = 3.5 Hz, 1H), 3.68 (s, 3H), 2.59 (td, *J* = 14.2, 6.1 Hz, 1H), 2.40 – 2.25 (m, 3H), 2.11 – 1.96 (m, 5H), 1.86 (td, *J* = 13.9, 5.1 Hz, 1H), 1.80 – 1.73 (m, 1H), 1.70 – 1.59 (m, 2H), 1.52 – 1.44 (m, 2H), 1.41 (s, 3H), 1.33 – 1.24 (m, 1H), 1.22 (s, 3H). ^{13}C NMR (101 MHz, $CDCl_3$) δ 214.63 (C), 173.95 (C), 86.28 (C), 77.11 (CH), 55.15 (CH_2), 51.66 (CH_3), 50.13 (CH), 49.08 (C), 44.78 (CH), 44.44 (C), 42.65 (CH_2), 40.67 (CH_2), 36.20 (CH_2), 33.91 (CH_2), 32.92 (CH_2), 29.56 (CH_2), 23.48 (CH_3), 23.05 (CH_3).. HRMS (ESI-QTOF) m/z: $[M + H]^+$ calcd for $C_{18}H_{27}O_4$ 307.1909, found 307.1913.

Synthesis of 4a.

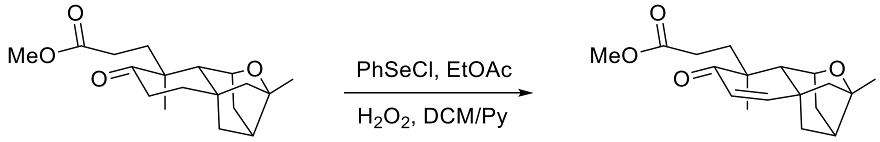

28 4a

To a solution of **28** (21mg, 0.07mmol) in EtOAc (2.5 mL), PHSeCl (21 mg, 0.11 mmol) was added. The solution was stirred for 22 h at room temperature under inert atmosphere. The solvent was then removed and the resulting crude was dissolved in 0.01 mL of Py and 2.5 mL of DCM. To this solution, 0.01 mL of H_2O_2 was added at 0 ºC. The 0 ºC bath was removed and the mixture refluxed (oil bath) for 10 min. The mixture was purified via flash chromatography using H/EtOAc (3:1) as eluent to give **4a** (17 mg, 82% yield). Spectroscopic data match with those reported on literature.[12]

*Compound **4a**.* Pale syrup. $[\alpha]_D$ -39.5 (c 1, DCM). ^{1}H NMR (400 MHz, $CDCl_3$) δ 6.48 (d, *J* = 10.1 Hz, 1H), 5.90 (d, *J* = 10.0 Hz, 1H), 4.41 (t, 1H), 3.68 (s, 3H), 2.42 (t, *J* = 6.5 Hz, 1H), 2.37 (s, 1H), 2.34 – 2.20 (m, 3H), 2.14 – 2.00 (m, 3H), 1.88 (dd, *J* = 11.1, 3.6 Hz, 1H), 1.83 – 1.71 (m, 2H), 1.63 (d, *J* = 11.1 Hz, 1H), 1.46 (s, 3H), 1.25 (s, 3H). ^{13}C NMR (126 MHz, $CDCl_3$) δ 203.2 (C), 173.8 (C), 153.5 (CH), 127.3 (CH), 87.0 (C), 76.5 (CH), 54.9 (CH_2), 51.6 (CH_3), 46.3 (C), 46.0 (CH), 46.0 (C), 44.7 (CH), 43.2 (CH_2), 40.6 (CH_2), 30.8 (CH_2), 29.1 (CH_2), 24.5 (CH_3), 23.0 (CH_3). HRMS (ESI-QTOF) m/z: $[M + H]^+$ calcd for $C_{18}H_{25}O_4$ 305.1753, found 305.1745.

References:

(1) Zhang, X.; Dong, L.-B.; Yang, L.-C.; Rudolf, J. D.; Shen, B.; Renata, H. Harnessing the Biocatalytic Potential of PtmO6, an α-Ketoglutarate-Dependent Dioxygenase from Platensimycin Biosynthesis, for the Chemoenzymatic Synthesis of Highly Oxidized Ent-Kaurane Diter-Penes. *ChemRxiv* **2020**. https://doi.org/10.26434/chemrxiv.7663112.v1

(2) Amaro-Luis, J. M. An Ent-Kaurenolide from Stevia Lucida. *Phytochemistry* **1993**, *32* (6). https://doi.org/10.1016/0031-9422(93)85190-3.

(3) Soares, A. C. F.; Matos, P. M.; Dias, H. J.; Aguiar, G. de P.; dos Santos, E. S.; Martins, C. H. G.; Veneziani, R. C. S.; Ambrósio, S. R.; Heleno, V. C. G. Variability of the Antibacterial Potential among Analogue Diterpenes against Gram-Positive Bacteria: Considerations on the Structure–Activity Relationship. *Canadian Journal of Chemistry* **2019**, *97* (7). https://doi.org/10.1139/cjc-2018-0369.

(4) Hesricki, C. A.; Jefferies, P. R. *The Chemistry of The Ecphorbiaceae*. *Australian Journal of Chemistry*. **1964**, *17*, 915.

(5) Jensen, N. P.; Johnson, W. S. A Three-Step Synthesis of Fichtelite from Abietic Acid. *The Journal of Organic Chemistry* **1967**, *32* (6). https://doi.org/10.1021/jo01281a097.

(6) Willand-Charnley, R.; Fisher, T. J.; Johnson, B. M.; Dussault, P. H. Pyidine Is an Organocatalyst for the Reductive Ozonolysis of Alkenes. *Organic Letters* **2012**, *14* (9), 2242–2245. https://doi.org/10.1021/ol300617r.

(7) Peixoto, A. F.; de Melo, D. S.; Fernandes, T. F.; Fonseca, Y.; Gusevskaya, E. v.; Silva, A. M. S.; Contreras, R. R.; Reyes, M.; Usubillaga, A.; dos Santos, E. N.; Pereira, M. M.; Bayón, J. C. Rhodium Catalyzed Hydroformylation of Kaurane Derivatives: A Route to New Diterpenes with Potential Bioactivity. *Applied Catalysis A: General* **2008**, *340* (2). https://doi.org/10.1016/j.apcata.2008.02.015.

(8) Batista, R.; García, P. A.; Castro, M. A.; Miguel del Corral, J. M.; Speziali, N. L.; de P. Varotti, F.; de Paula, R. C.; García-Fernández, L. F.; Francesch, A.; San Feliciano, A.; de Oliveira, A. B. Synthesis, Cytotoxicity and Antiplasmodial Activity of Novel Ent -Kaurane Derivatives. *European Journal of Medicinal Chemistry* **2013**, *62*. https://doi.org/10.1016/j.ejmech.2012.12.010.

(9) Lewis, N. J.; MacMillan, J. Terpenoids. Part 8. Partial Syntheses of Ent-11β-, 12α-, and 12β-Hydroxykaur-16-En-19-Oic Acids from Grandiflorenic Acid. *J. Chem. Soc., Perkin Trans. 1* **1980**, *0*. https://doi.org/10.1039/P19800001270.

(10) James Mcalees A N D, A.; Mccrindle', R. Ring C Functionalized Diterpenoids. Part I. The Hypoiodite Reaction as a Route to Ring C Functionalized Kauranes and Phyllocladanes. *Canadian Journal of Chemistry* **1973**, *51*. https://doi.org/10.1139/v73-614

(11) Dong, L.-B.; Zhang, X.; Rudolf, J. D.; Deng, M.-R.; Kalkreuter, E.; Cepeda, A. J.; Renata, H.; Shen, B. Cryptic and Stereospecific Hydroxylation, Oxidation, and Reduction in Platensimycin and Platencin Biosynthesis. *Journal of the American Chemical Society* **2019**, *141* (9). https://doi.org/10.1021/jacs.8b13452.

(12) Nicolaou, K. C.; Li, A.; Edmonds, D. J.; Tria, G. S.; Ellery, S. P. Total Synthesis of Platensimycin and Related Natural Products. *Journal of the American Chemical Society* **2009**, *131* (46). https://doi.org/10.1021/ja9068003.

(13) Batista, R.; García, P. A.; Castro, M. A.; del Corral, J. M. M.; San Feliciano, A.; Oliveira, A. B. de. New Oxidized Ent-Kaurane and Ent-Norkaurane Derivatives from Kaurenoic Acid. *Journal of the Brazilian Chemical Society* **2007**, *18* (3). https://doi.org/10.1590/S0103-50532007000300020.

(14) Batista, R.; Humberto, J. L.; Chiari, E.; de Oliveira, A. B. Synthesis and Trypanocidal Activity of Ent-Kaurane Glycosides. *Bioorganic & Medicinal Chemistry* **2007**, *15* (1). https://doi.org/10.1016/j.bmc.2006.09.048.

(15) Node, M.; Kajimoto, T.; Fujita, E.; Fuji, K. Regioselective Hydroxylation in the C Ring of Ent-Kaurene; Syntheses of Ent-11α-Hydroxykaurene and Ent-11α-Hydroxykauren-15-One. *Bulletin of the Institute for Chemical Research, Kyoto University* **1987**, *65*, 129-133.

^{1}H-NMR, ^{13}C-NMR and Bidimensional Spectra

^{1}H NMR of **2** (400 MHz, $CDCl_3$)

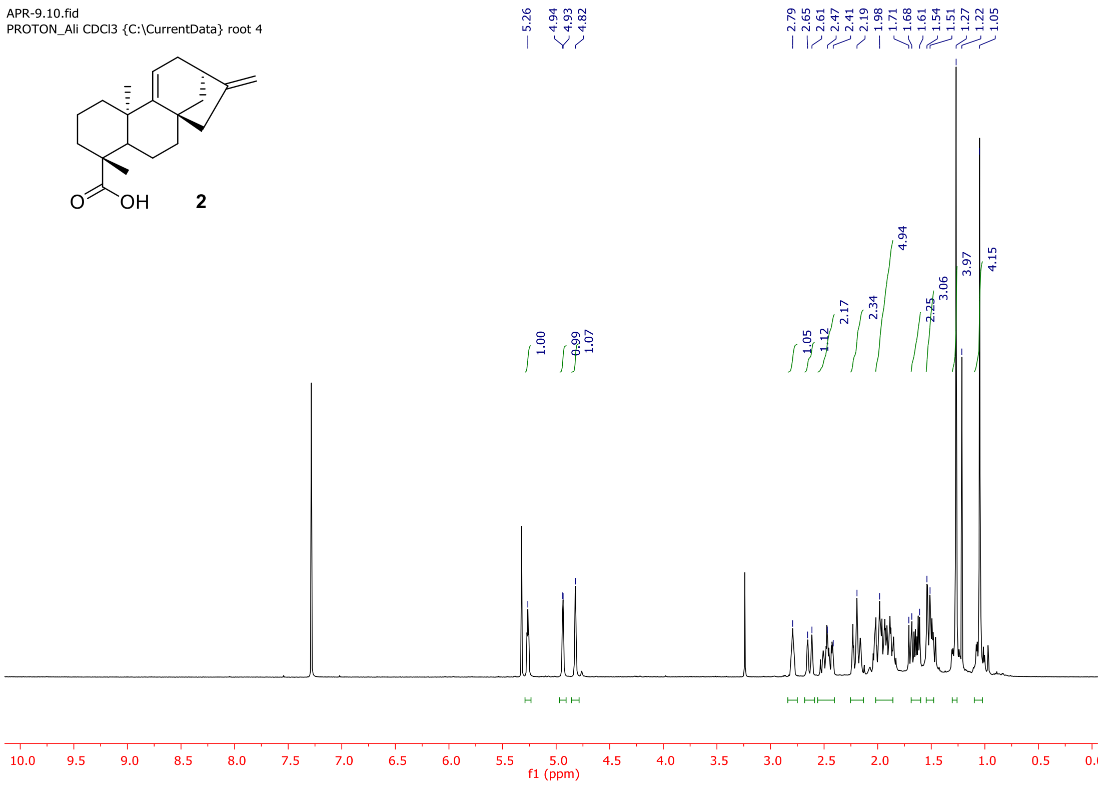

^{13}C{^{1}H} NMR of **2** (100 MHz, $CDCl_3$)

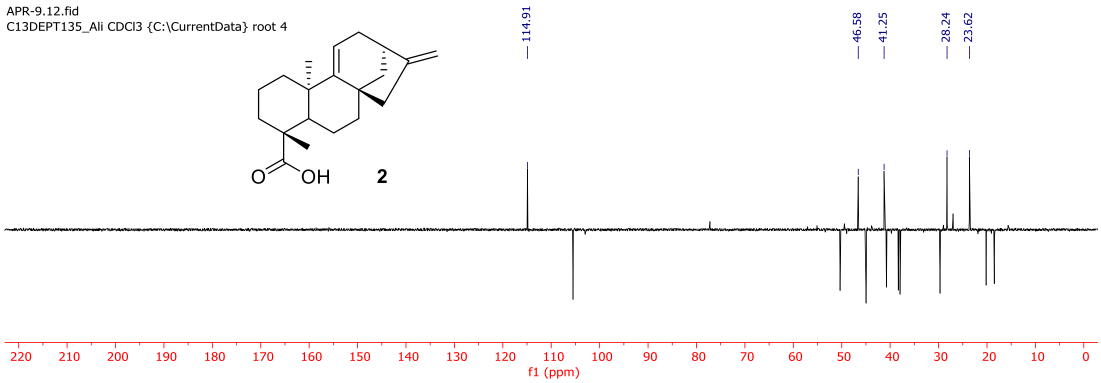

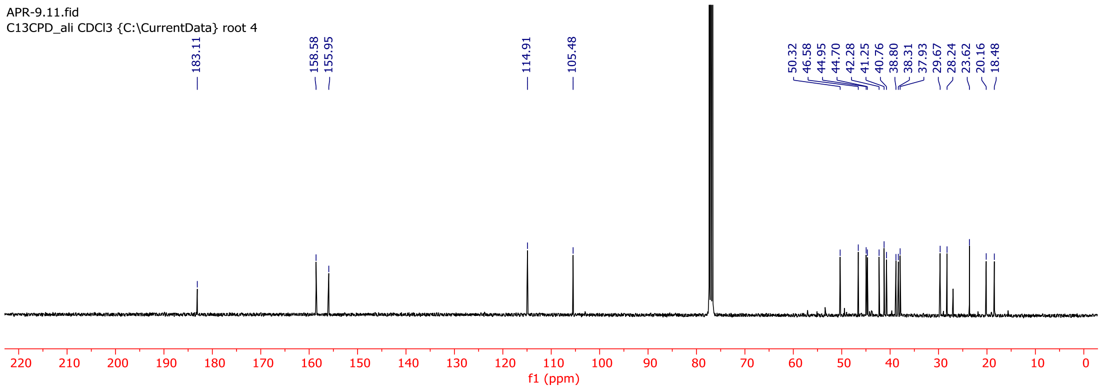

HSQC of 2 (400/100 MHz, $CDCl_3$)

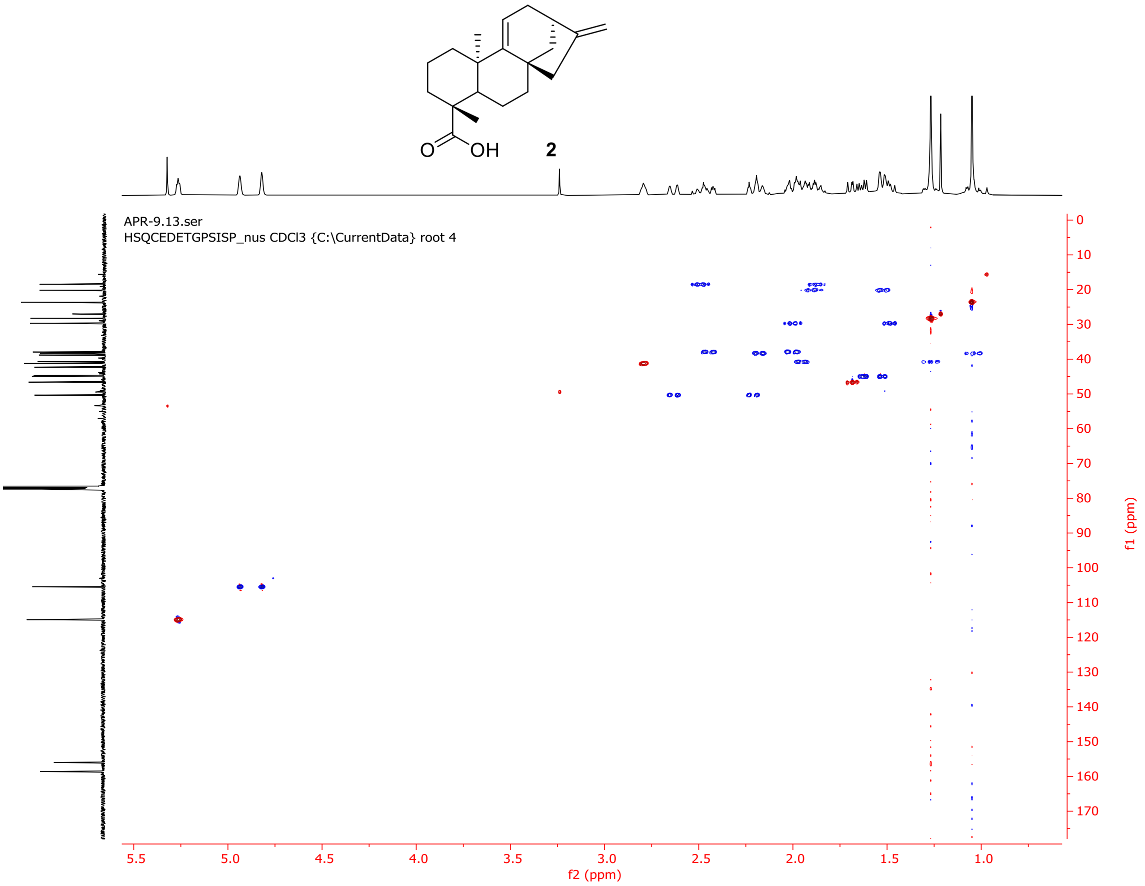

^{1}H NMR of **3a** (500 MHz, $CDCl_3$)

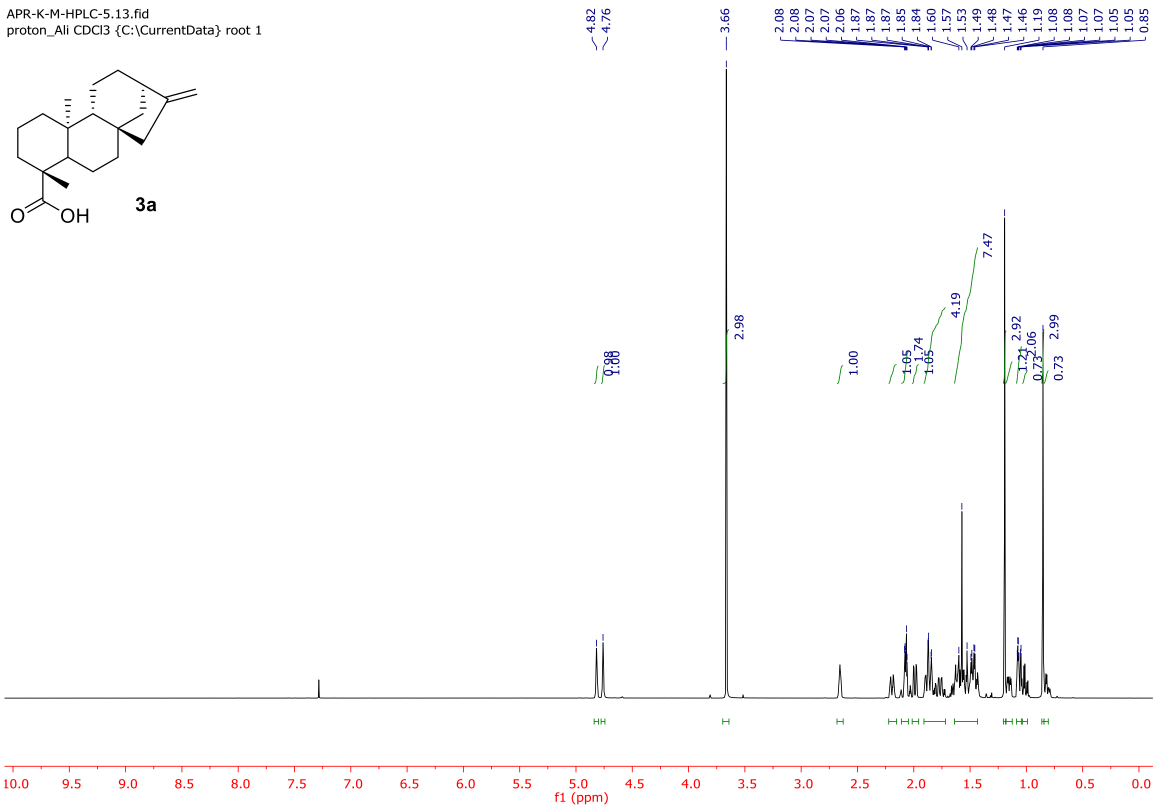

$^{13}C\{^{1}H\}$ NMR of **3a** (125 MHz, $CDCl_3$)

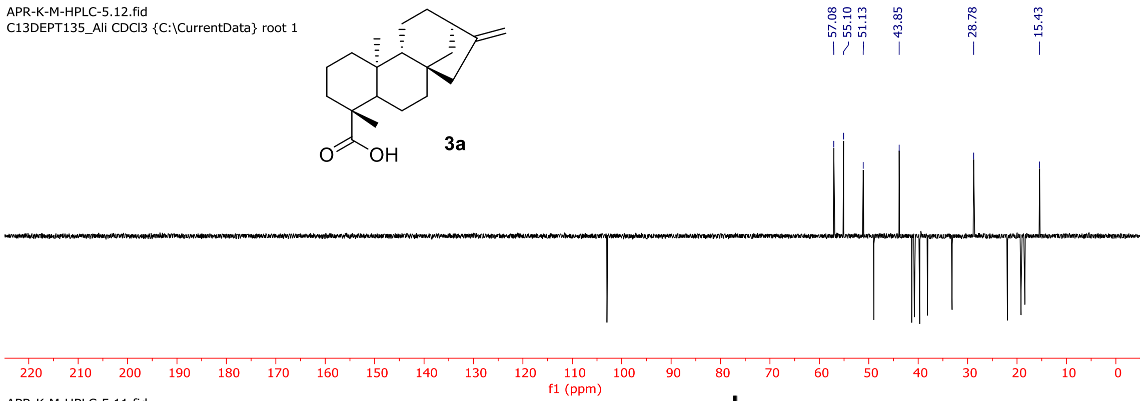

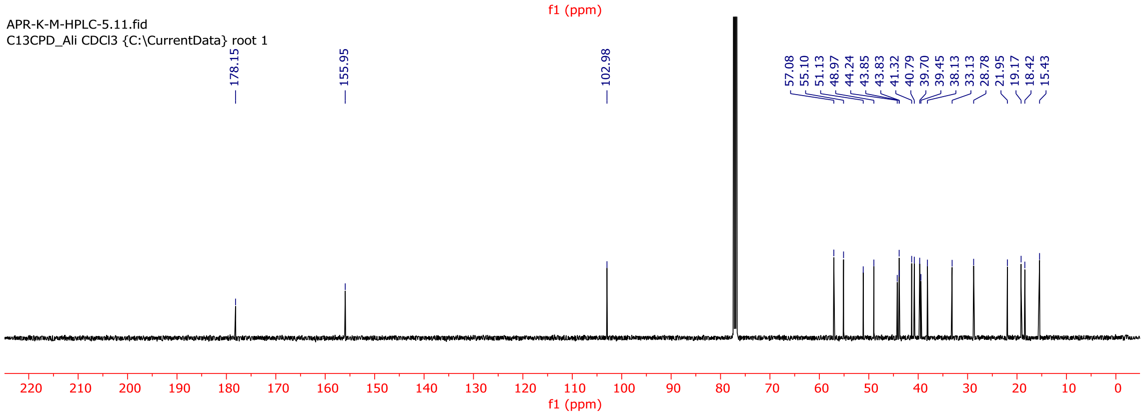

HSQC of 3a (500/125 MHz, $CDCl_3$)

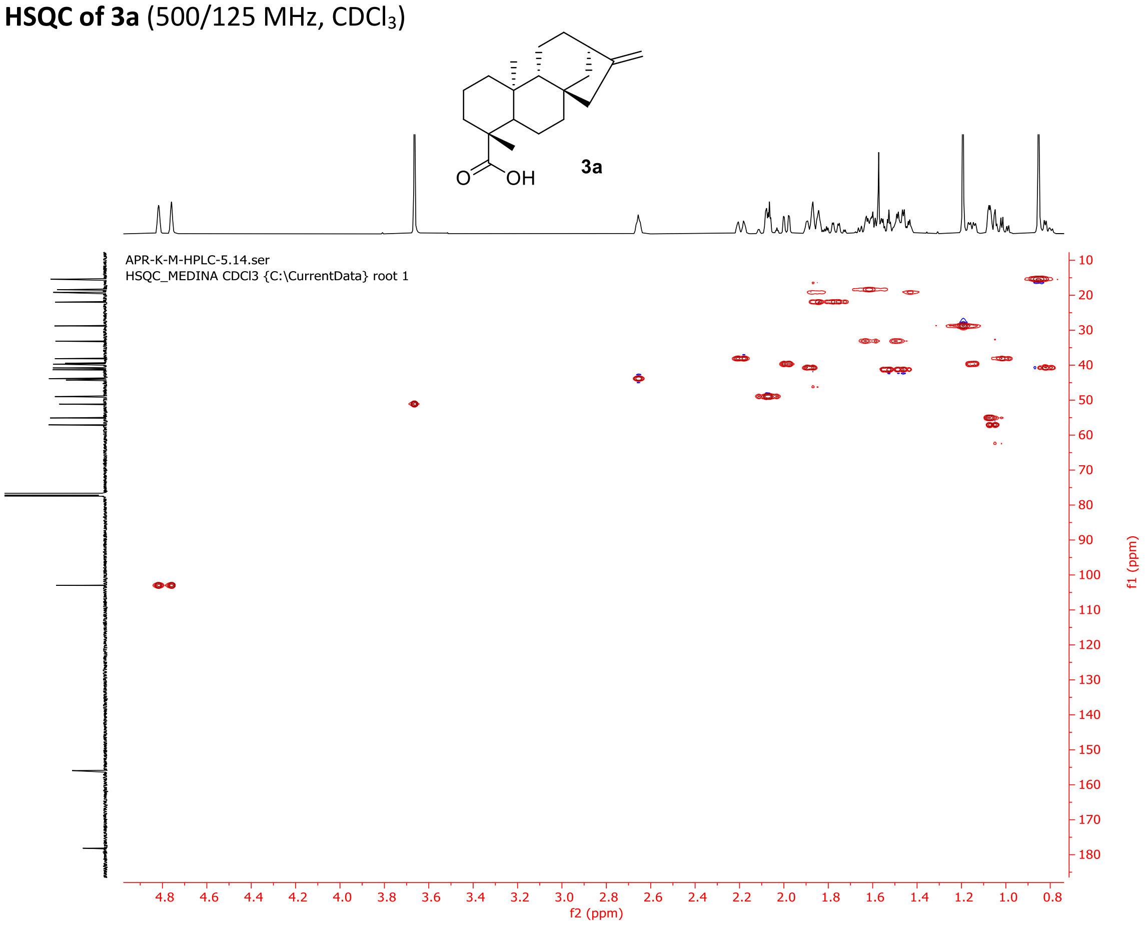

^{1}H NMR of **7** (500 MHz, $CDCl_3$)

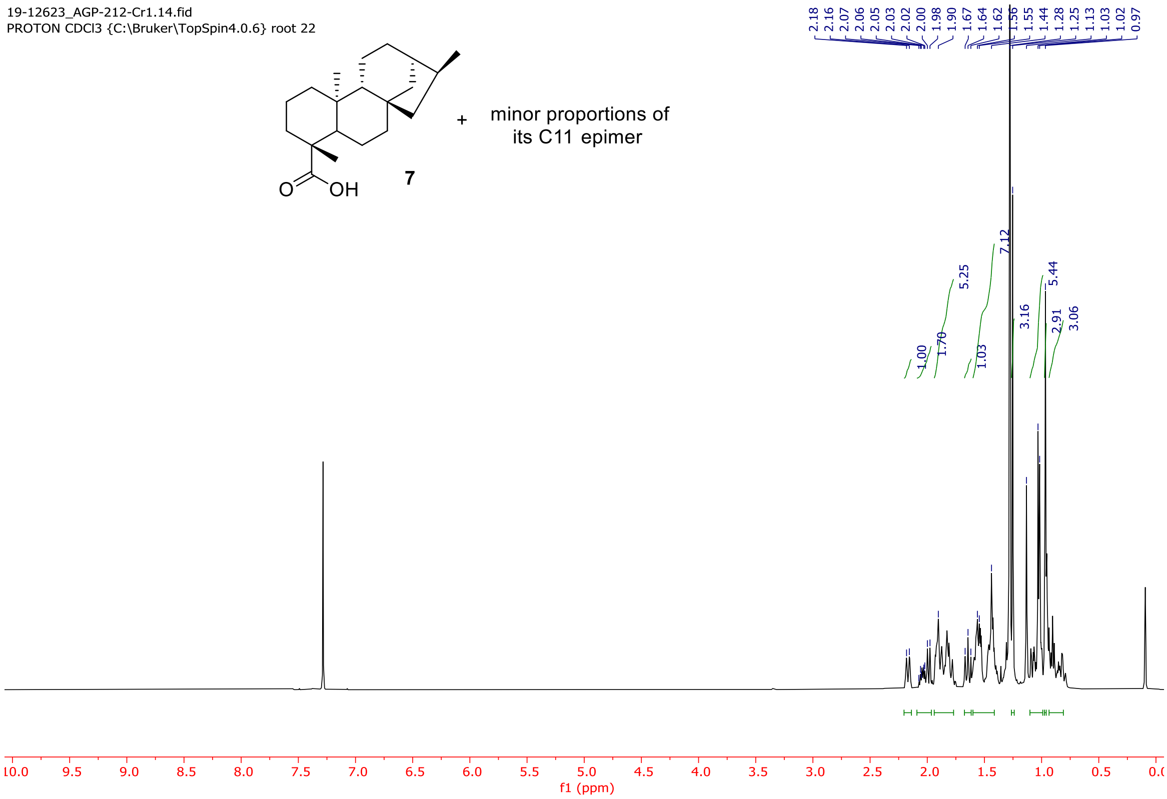

$^{13}C\{^{1}H\}$ NMR of **7** (125 MHz, $CDCl_3$)

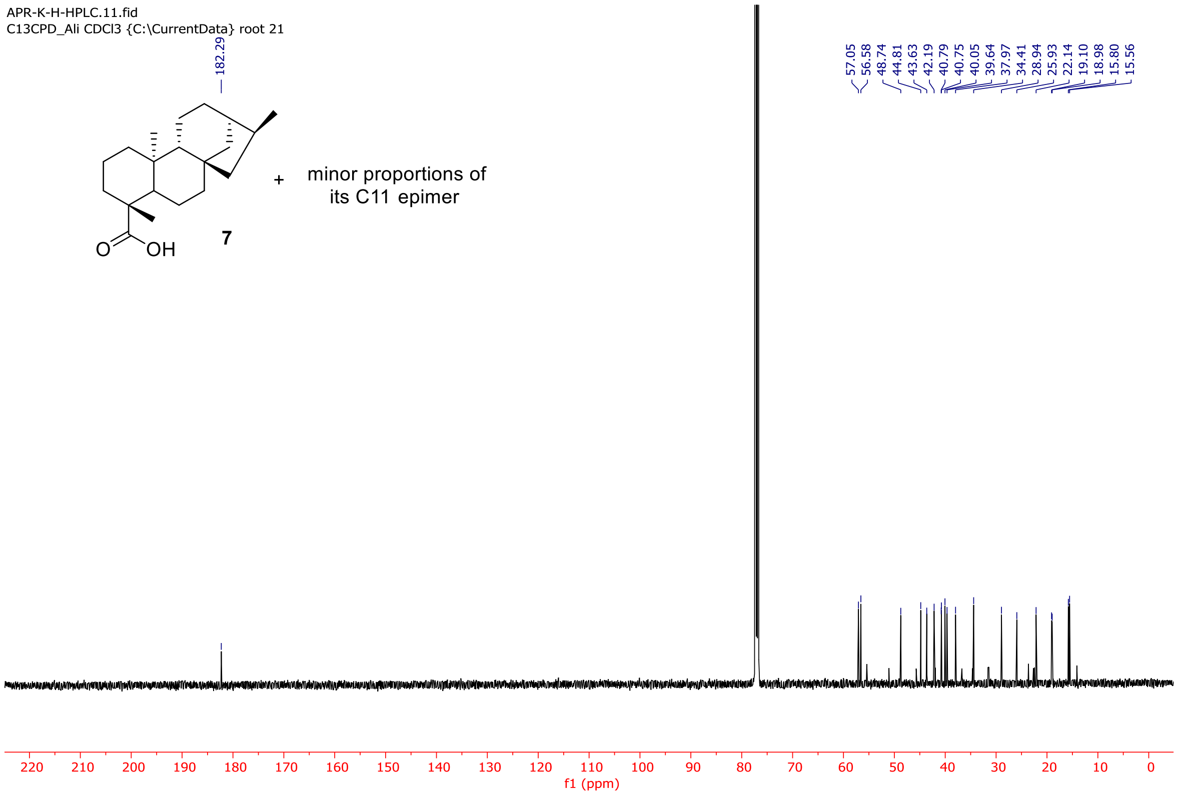

HSQC of 7 (500/125 MHz, $CDCl_3$)

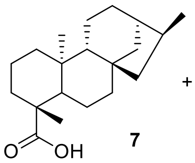

+ minor proportions of its C11 epimer

7

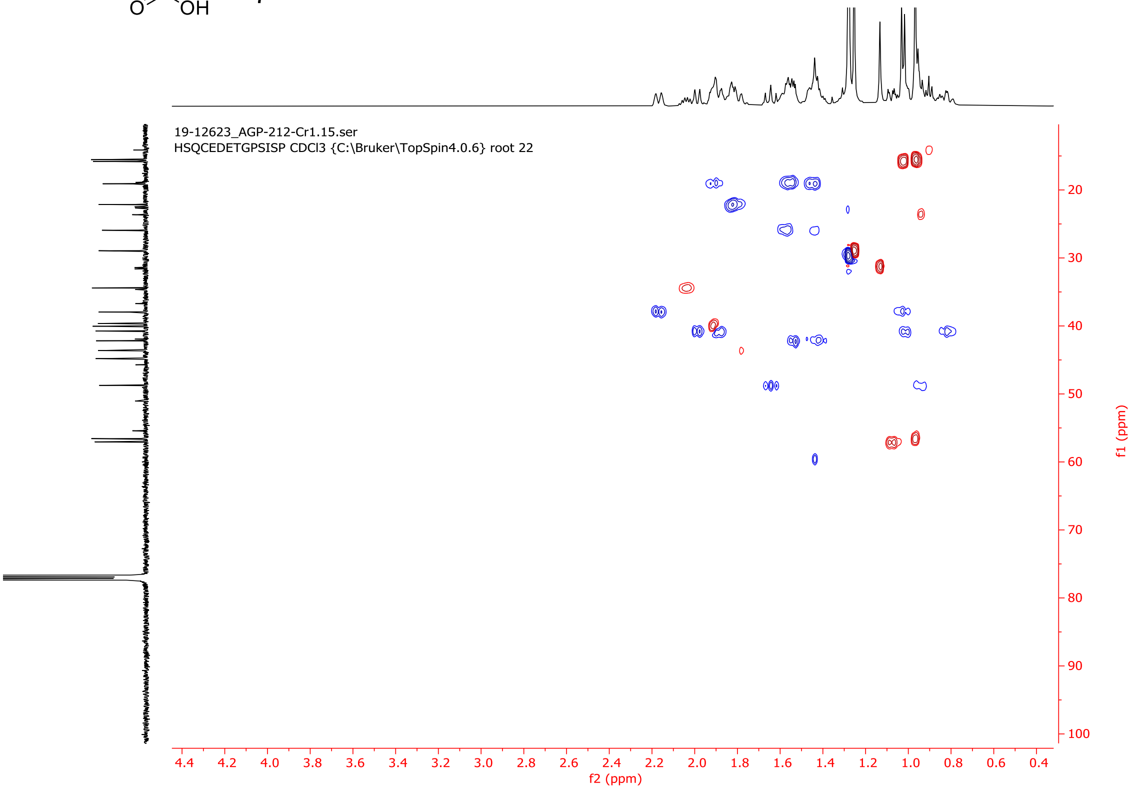

^{1}H NMR of **8** (500 MHz, $CDCl_3$)

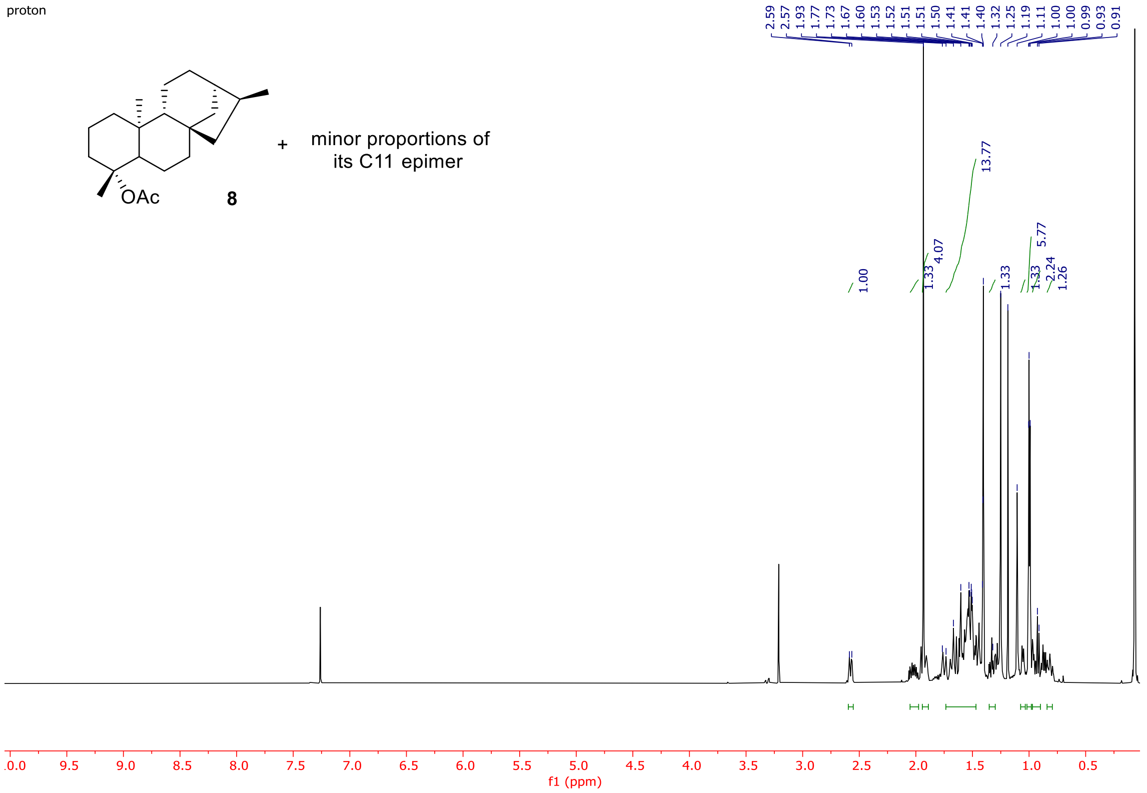

$^{13}C\{^{1}H\}$ NMR of **8** (125 MHz, $CDCl_3$)

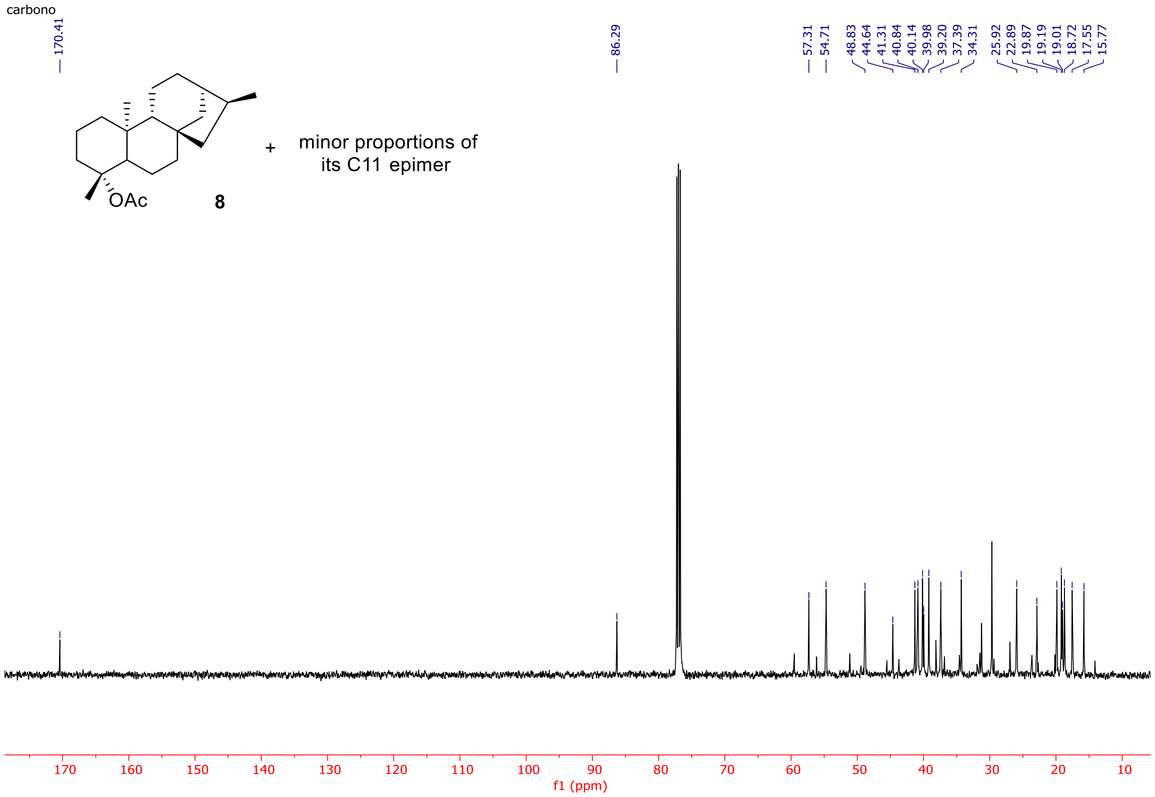

HSQC of 8 (500/125 MHz, $CDCl_3$)

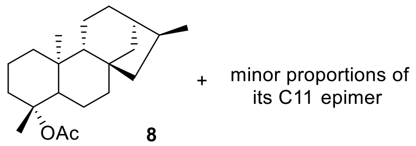

8

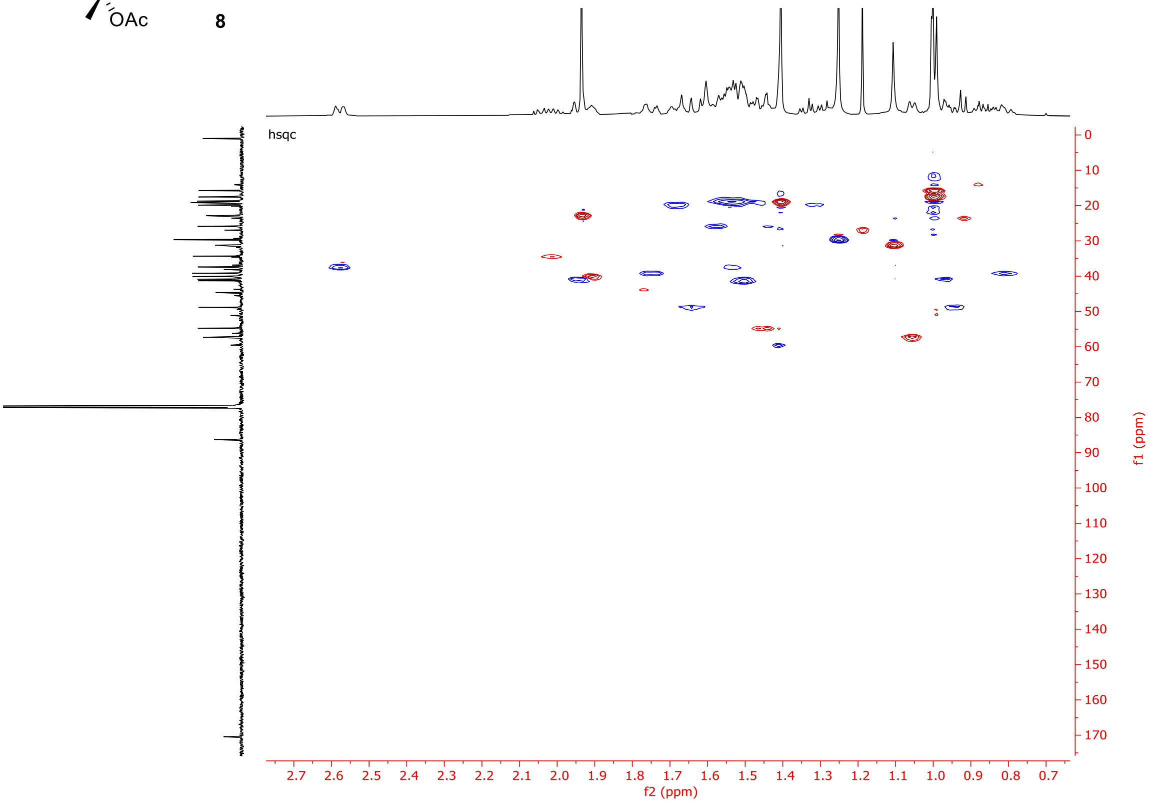

^{1}H NMR of **9c** (500 MHz, $CDCl_3$)

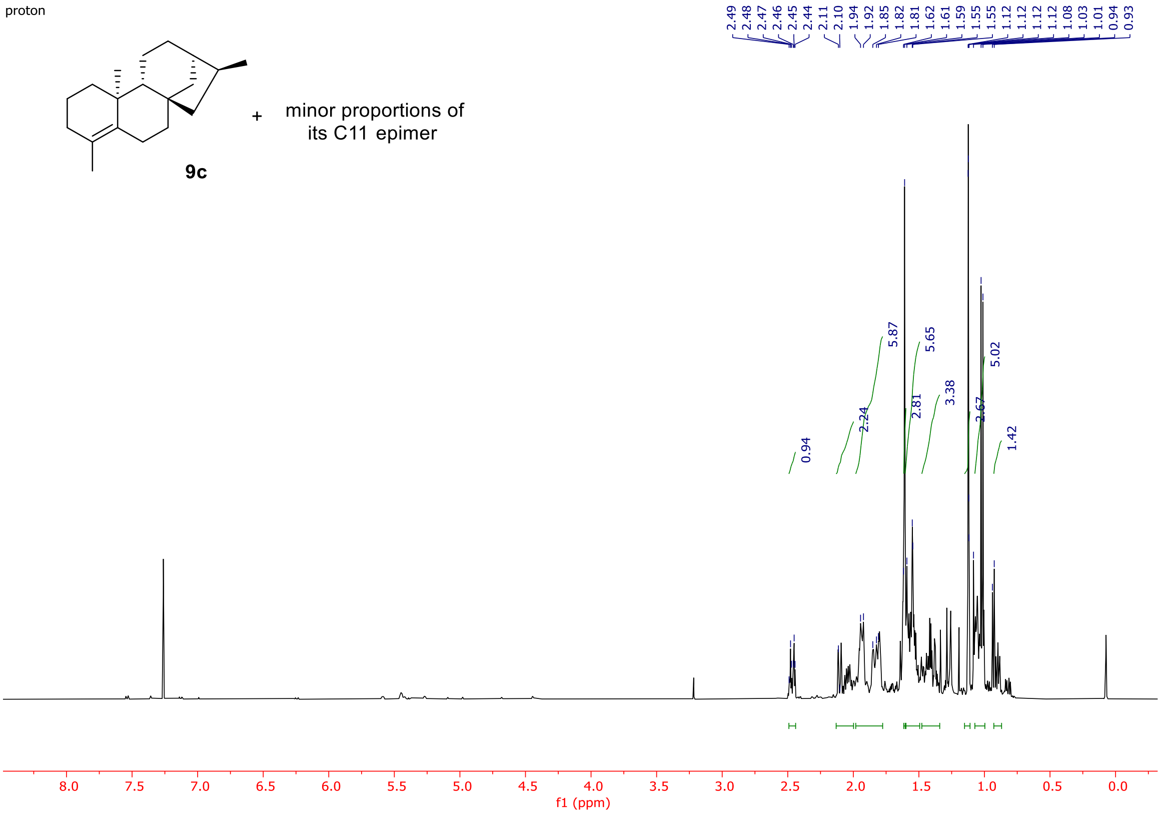

^{13}C{^{1}H} NMR of **9c** (125 MHz, $CDCl_3$)

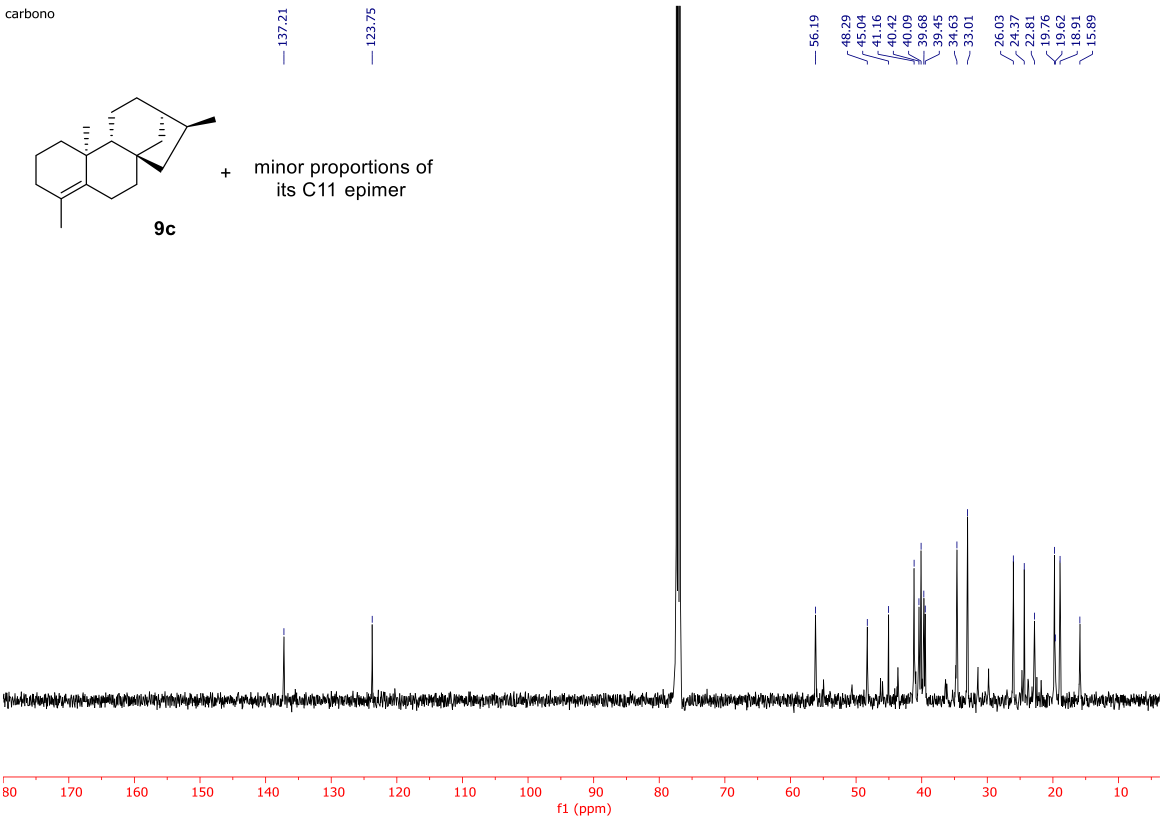

HSQC of 9c (500/125 MHz, $CDCl_3$)

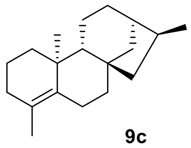

9c

+ minor proportions of its C11 epimer

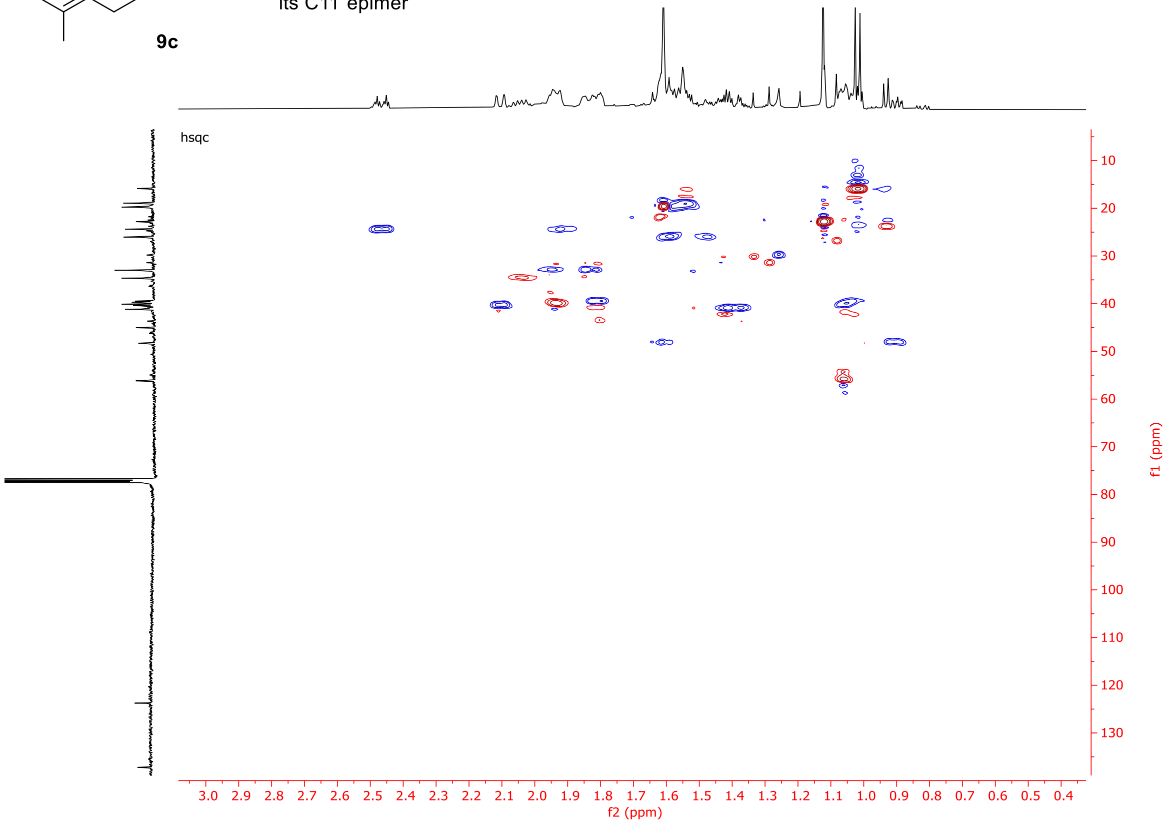

^{1}H NMR of **10** (500 MHz, $CDCl_3$)

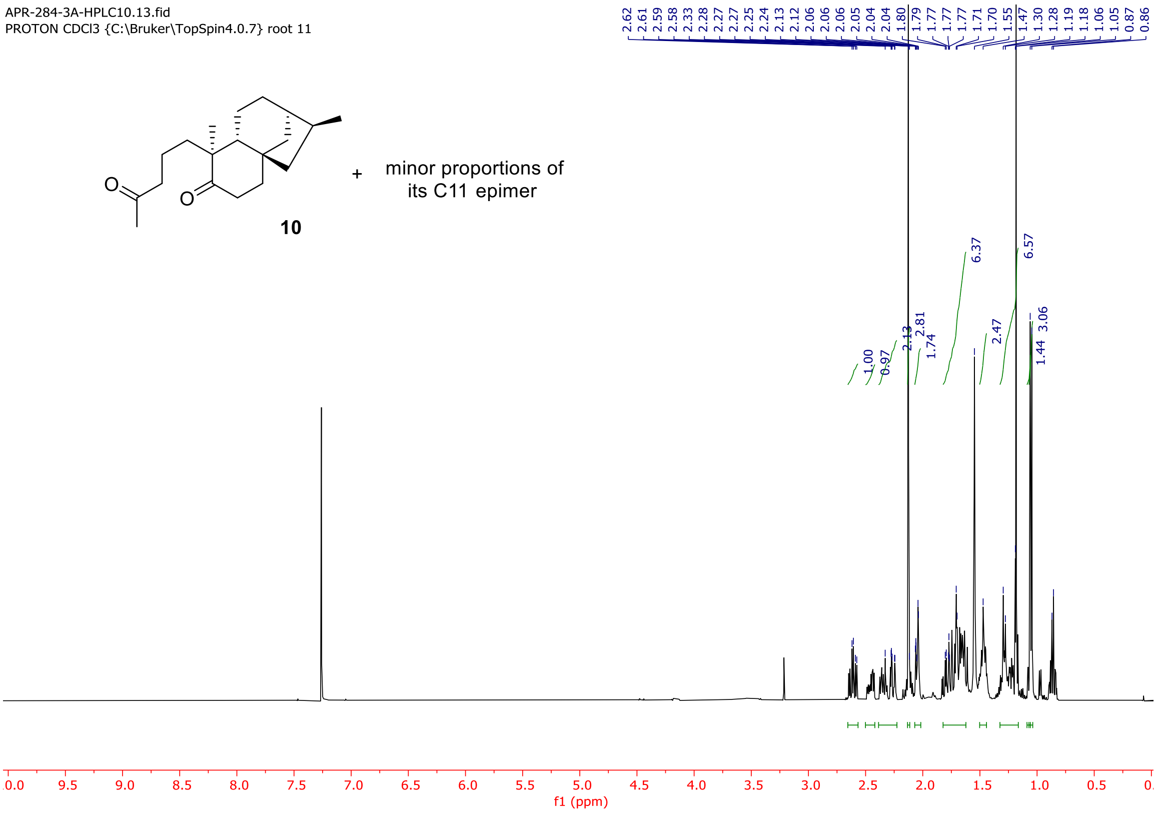

$^{13}C\{^{1}H\}$ NMR of **10** (125 MHz, $CDCl_3$)

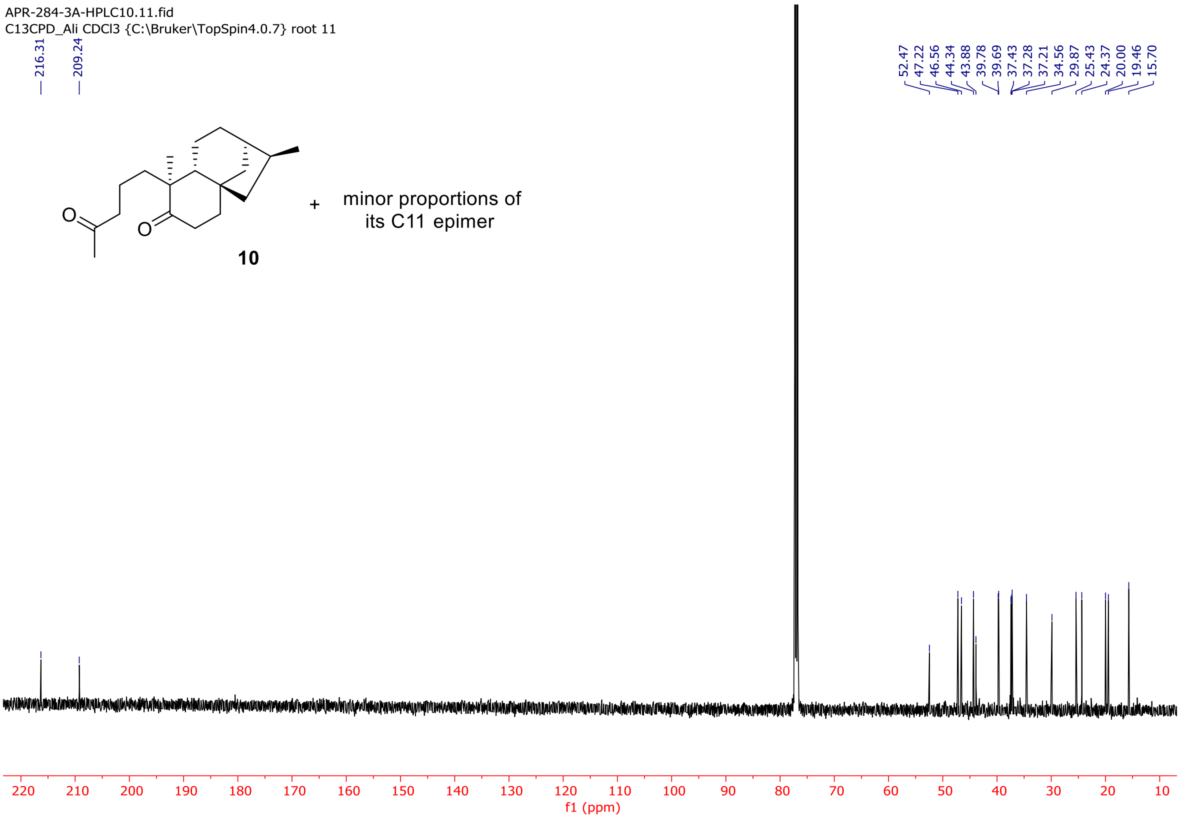

HSQC of 10 (500/125 MHz, $CDCl_3$)

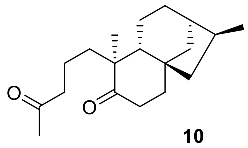

10

+ minor proportions of its C11 epimer

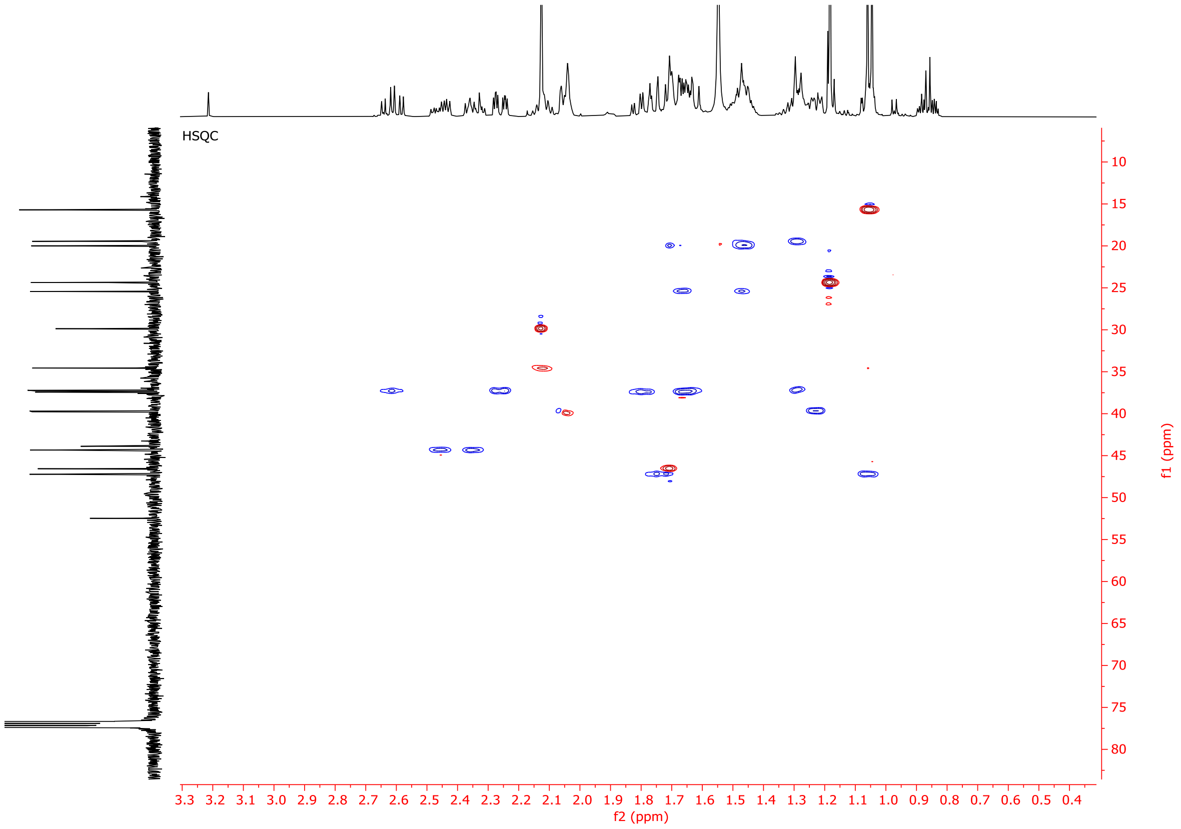

^{1}H NMR of **11** (500 MHz, $CDCl_3$)

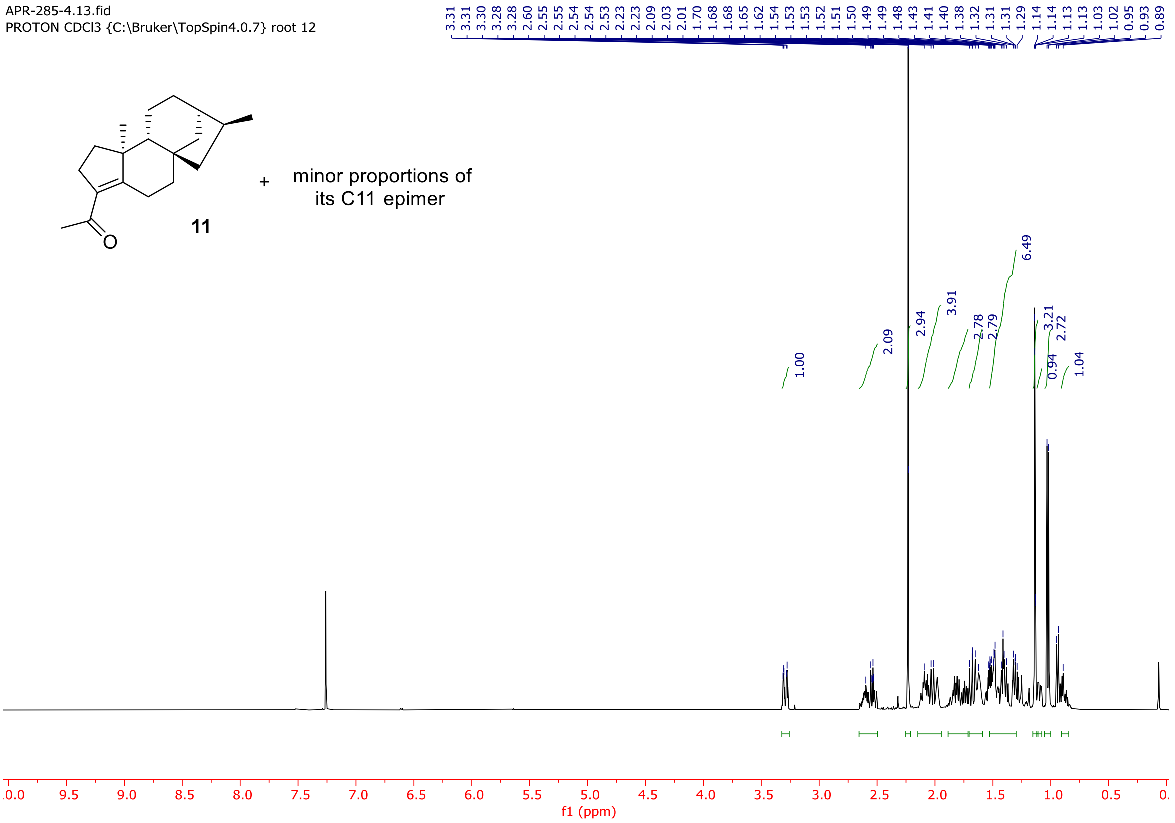

$^{13}C\{^{1}H\}$ NMR of **11** (125 MHz, $CDCl_3$)

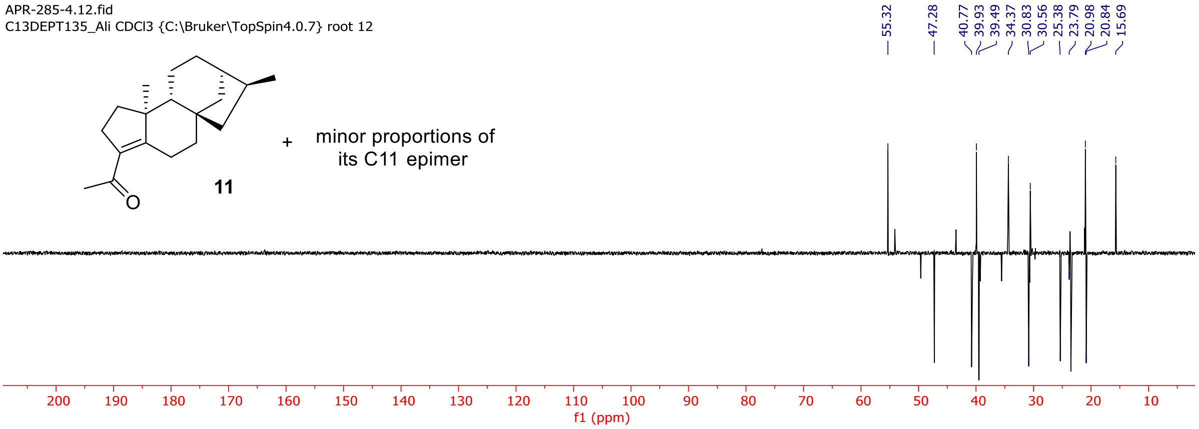

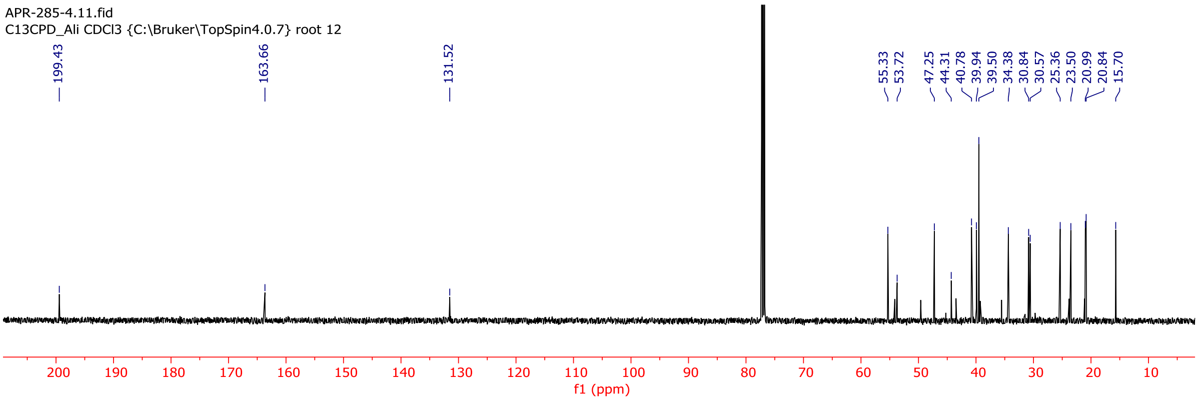

HSQC of 11 (500/125 MHz, $CDCl_3$)

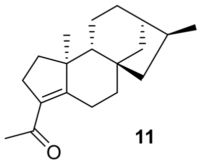

11

+ minor proportions of its C11 epimer

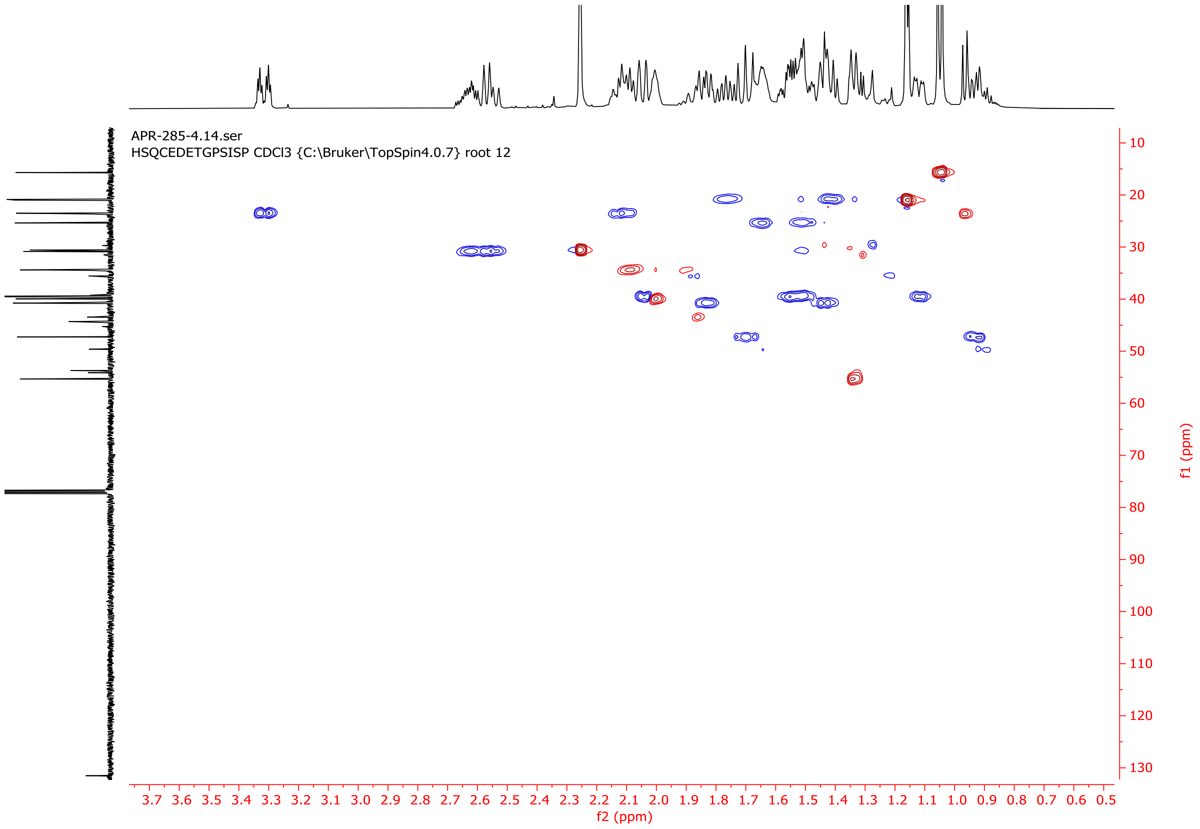

^{1}H NMR of **12** (500 MHz, $CDCl_3$)

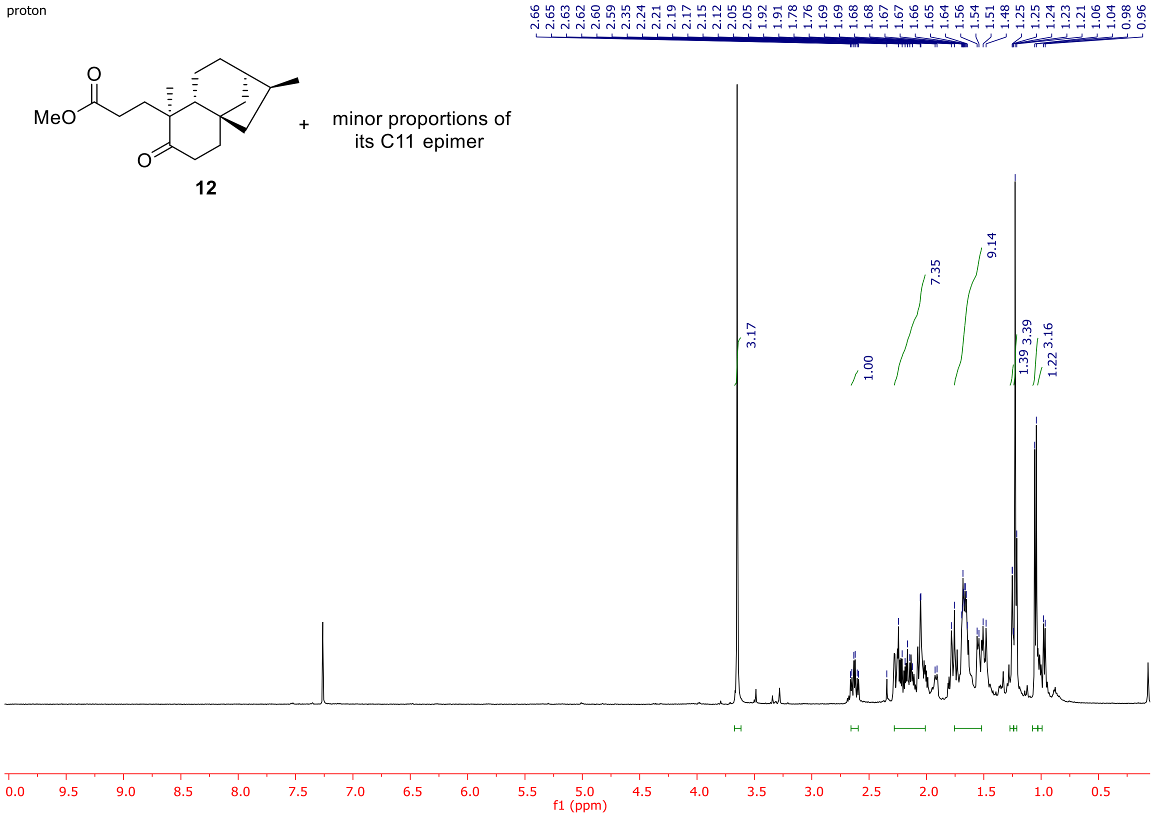

^{13}C{^{1}H} NMR of **12** (125 MHz, $CDCl_3$)

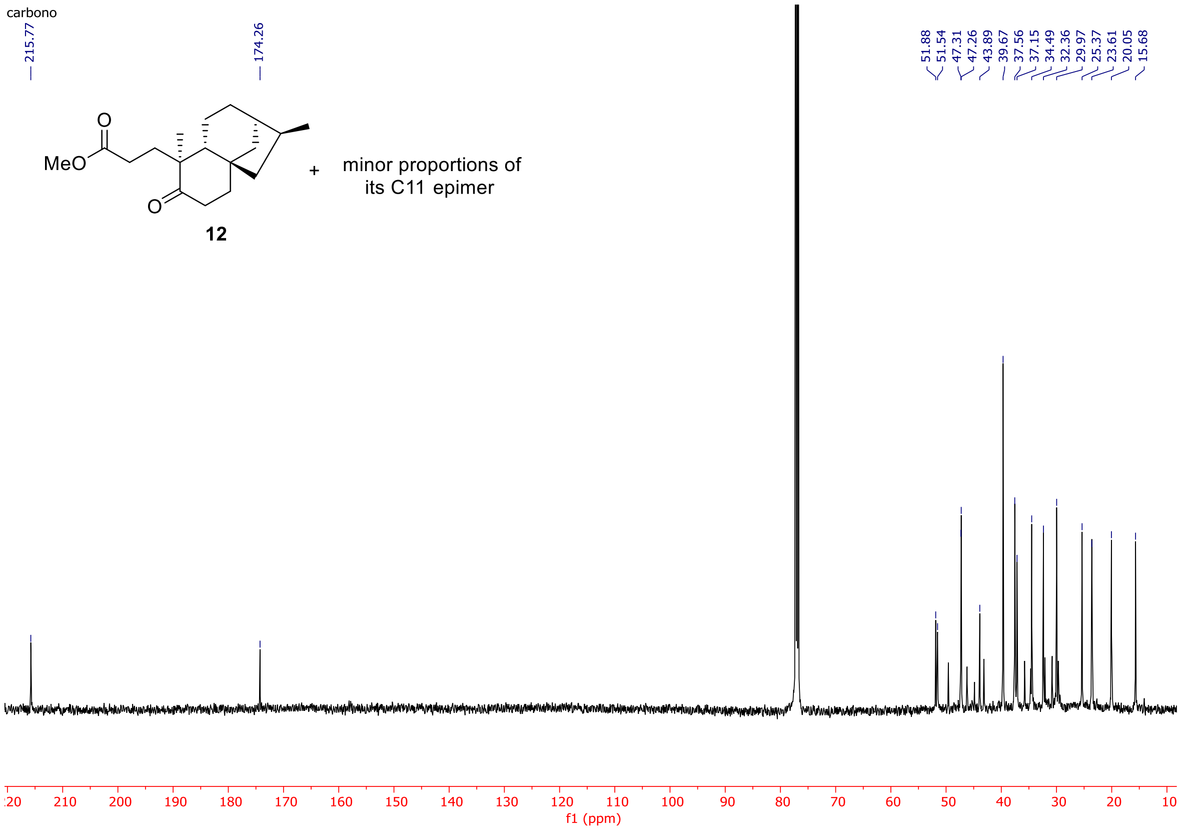

HSQC of 12 (500/125 MHz, $CDCl_3$)

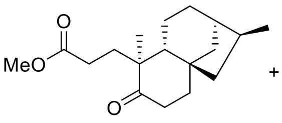

12

+ minor proportions of its C11 epimer

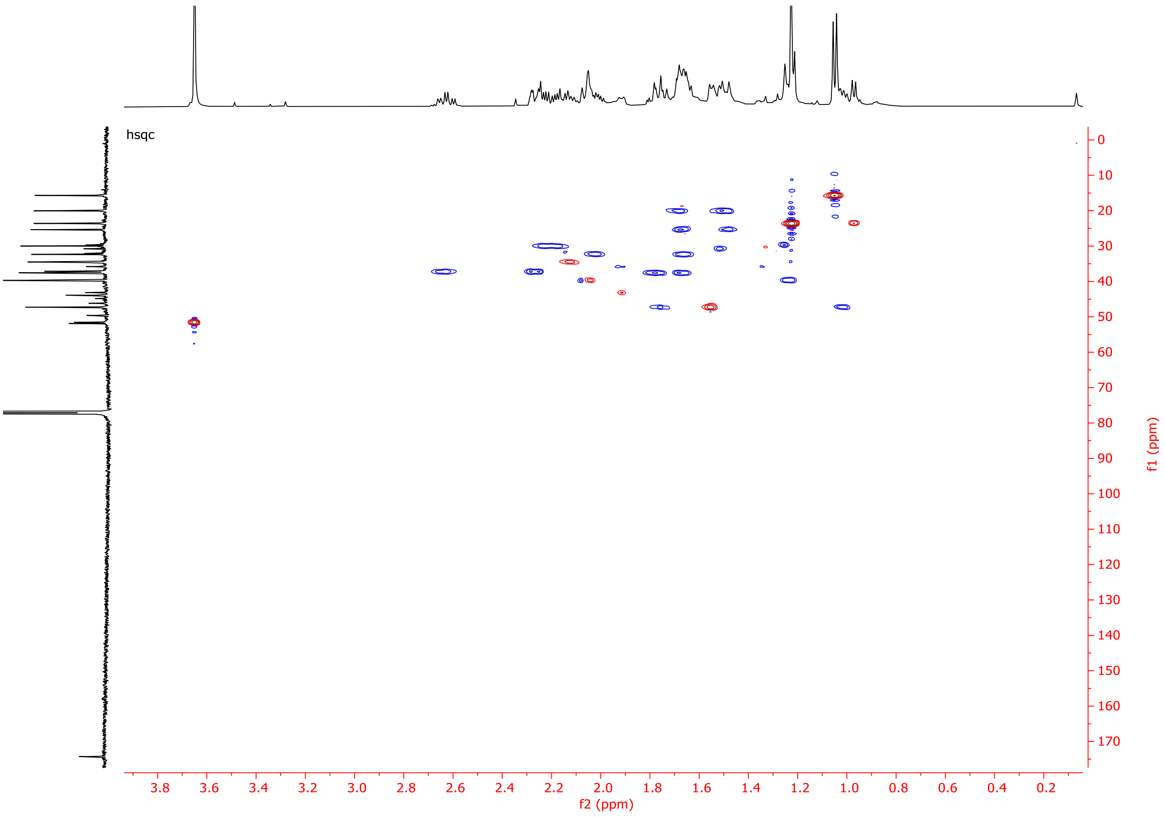

^{1}H NMR of **13** (500 MHz, $CDCl_3$)

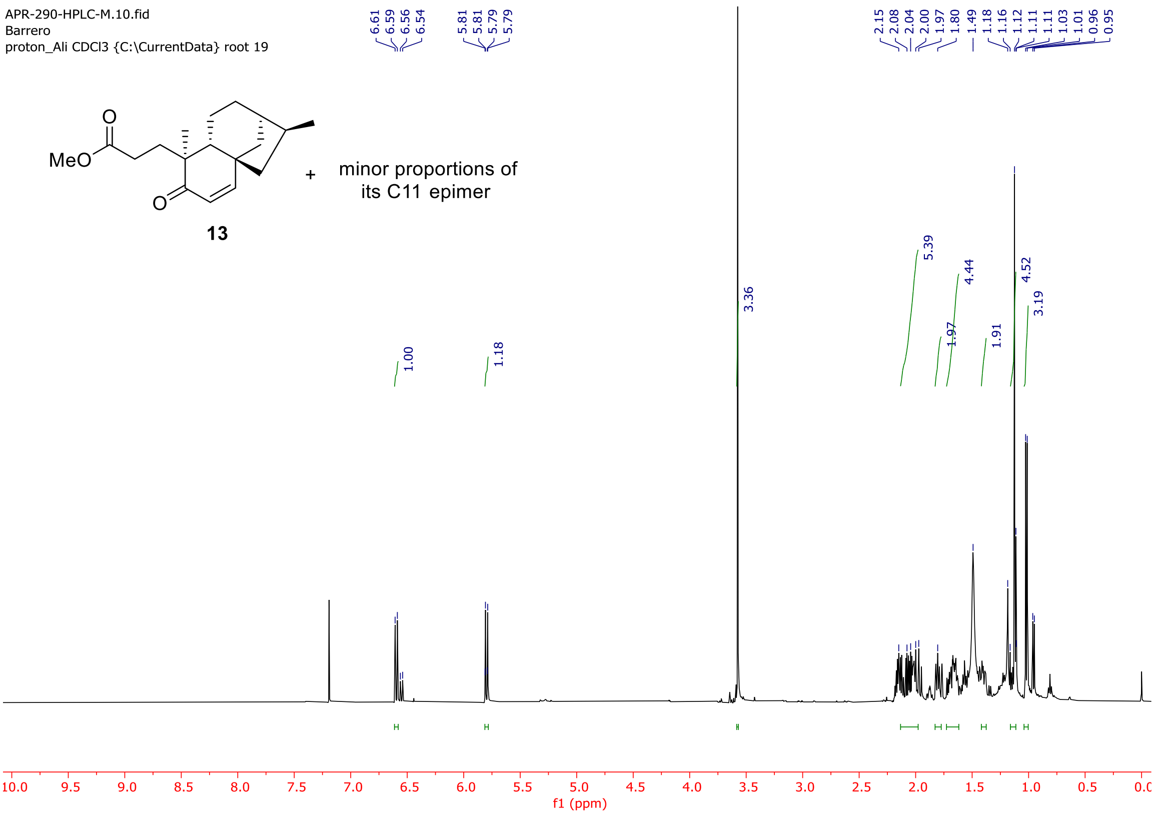

^{13}C{^{1}H} NMR of **13** (125 MHz, $CDCl_3$)

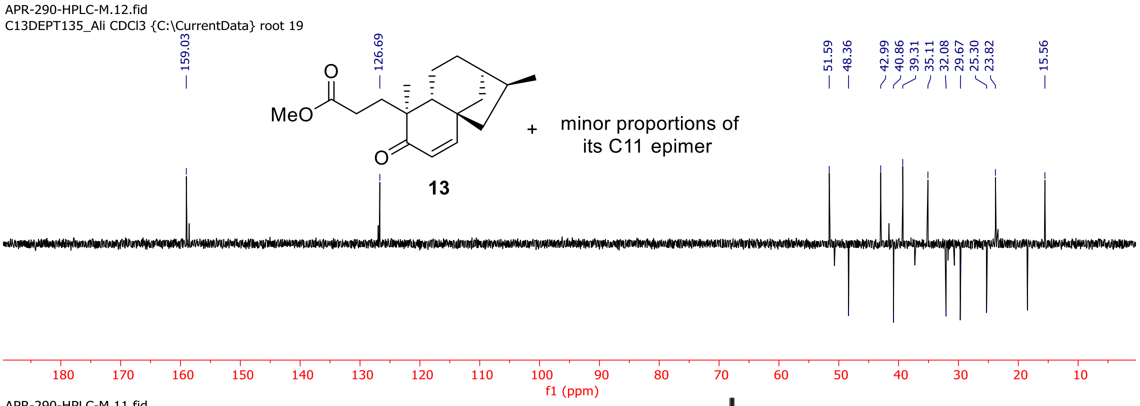

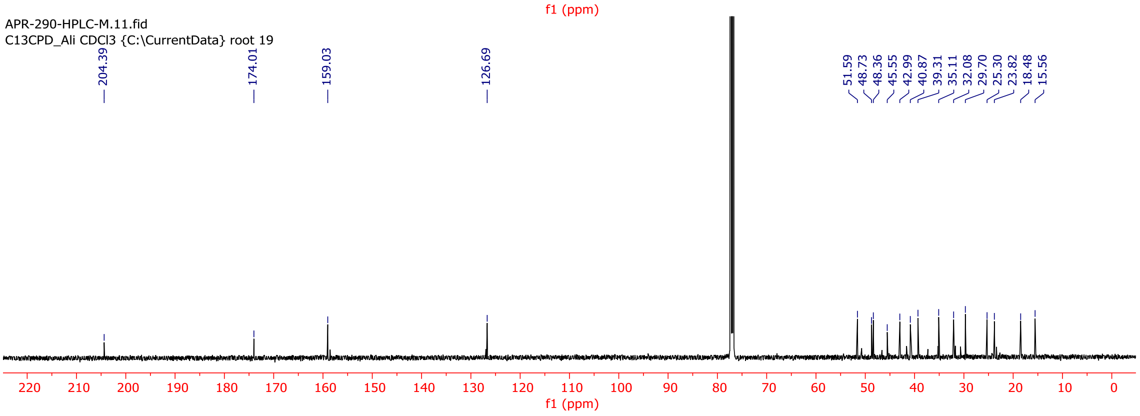

HSQC of 13 (500/125 MHz, $CDCl_3$)

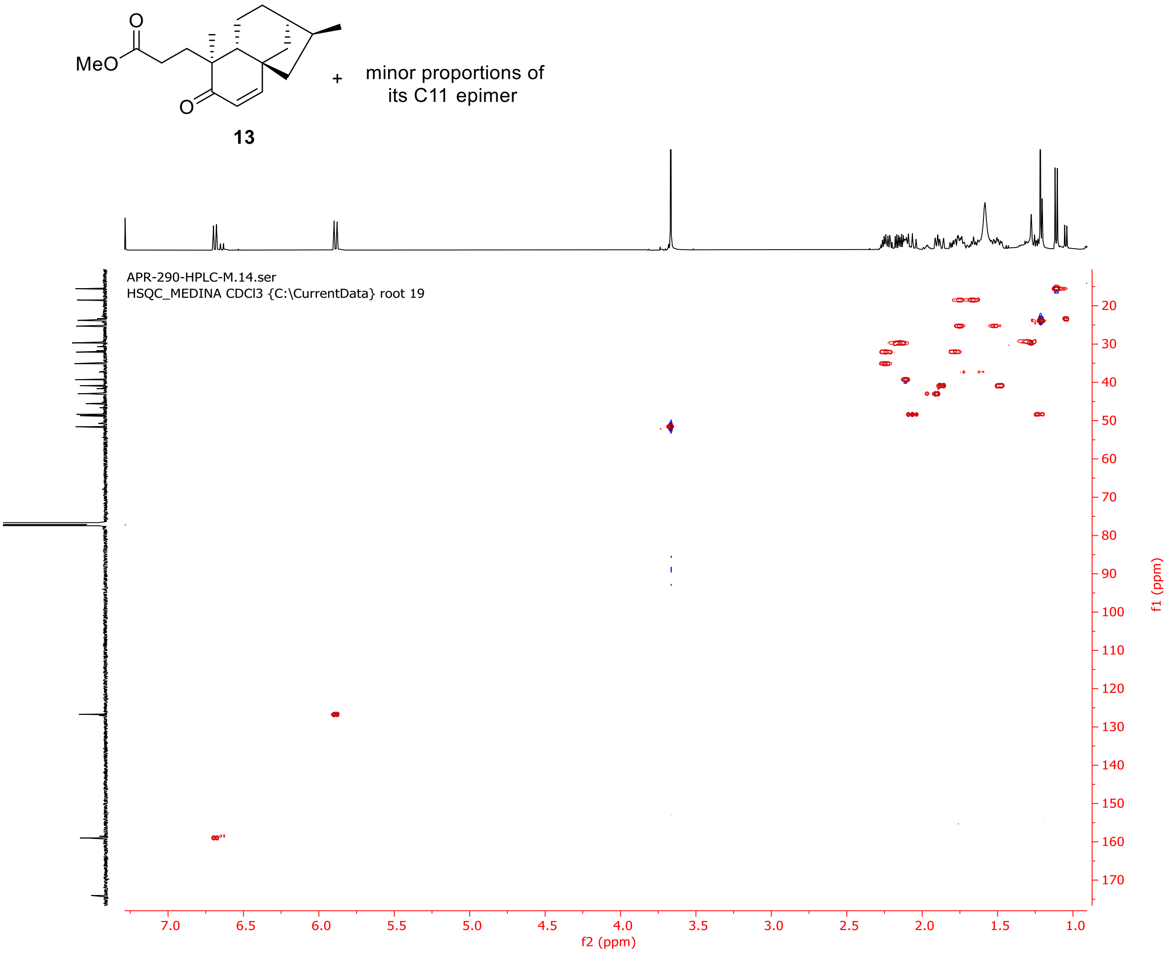

^{1}H NMR of **14** (500 MHz, $CDCl_3$)

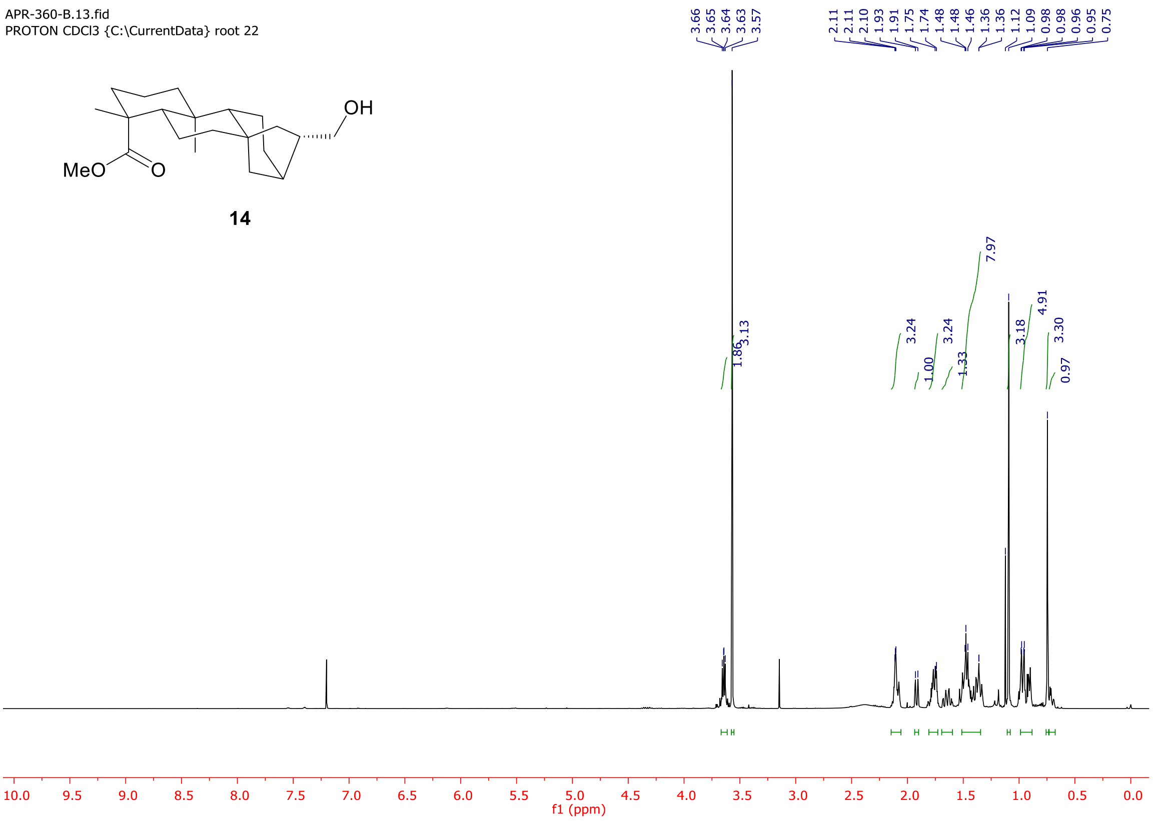

^{13}C{^{1}H} NMR of **14** (125 MHz, $CDCl_3$)

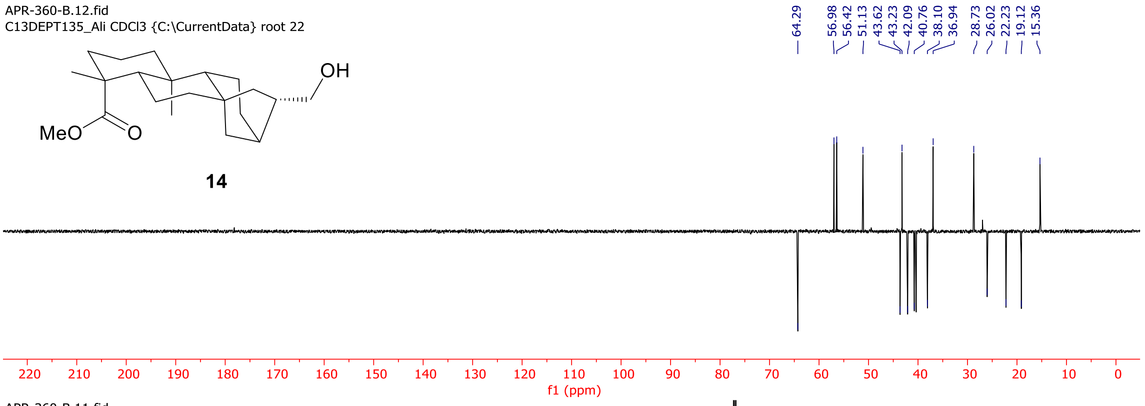

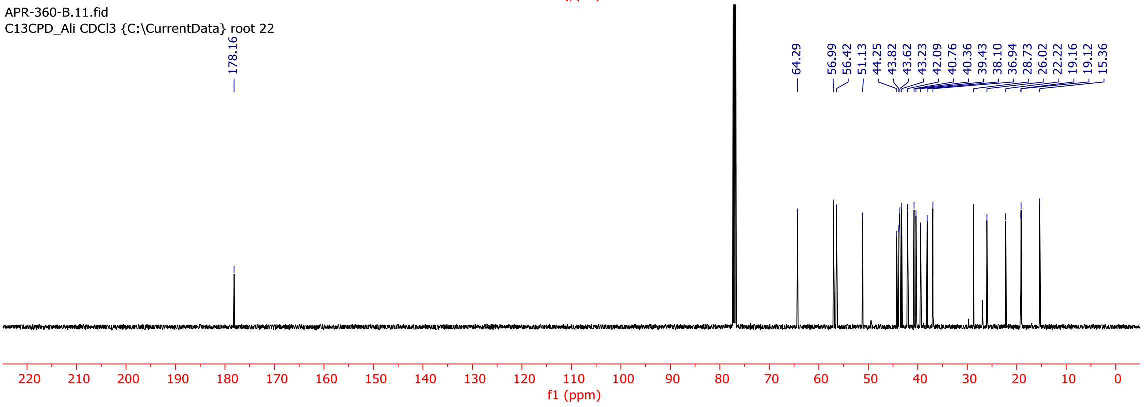

HSQC of 14 (500/125 MHz, $CDCl_3$)

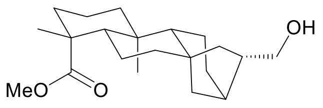

14

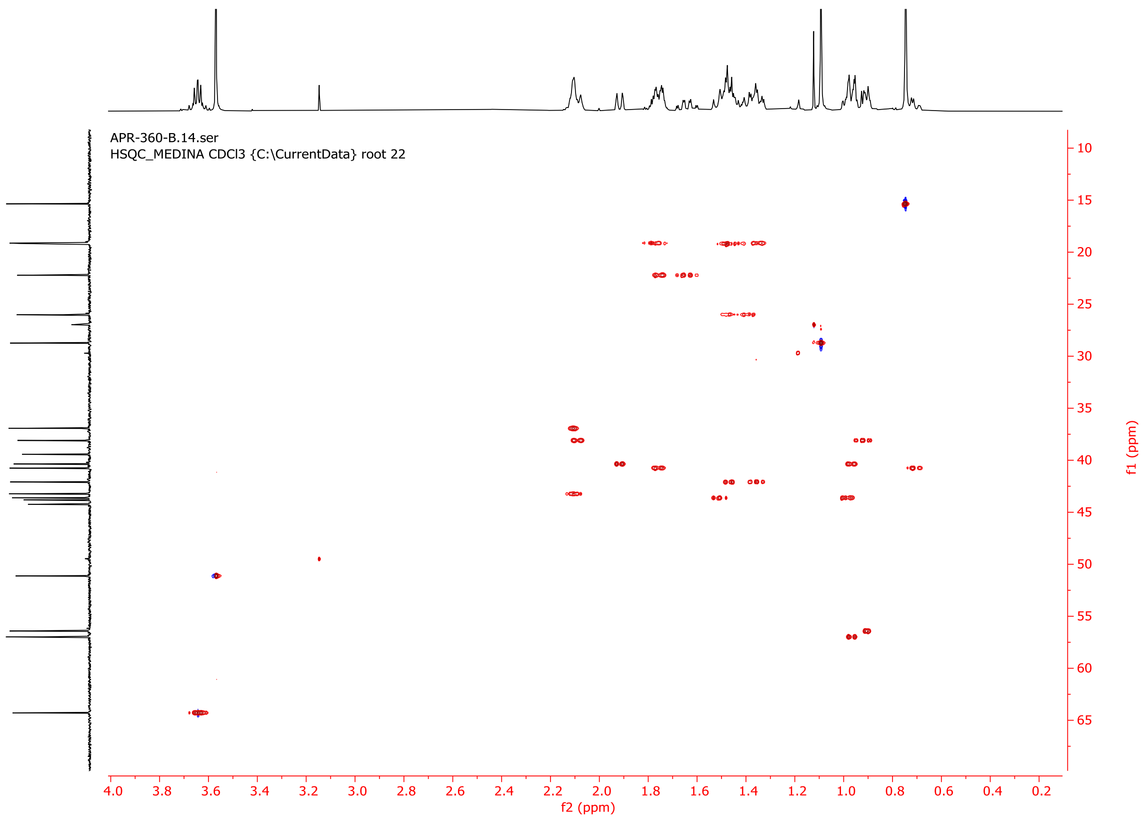

^{1}H NMR of **15** (500 MHz, $CDCl_3$)

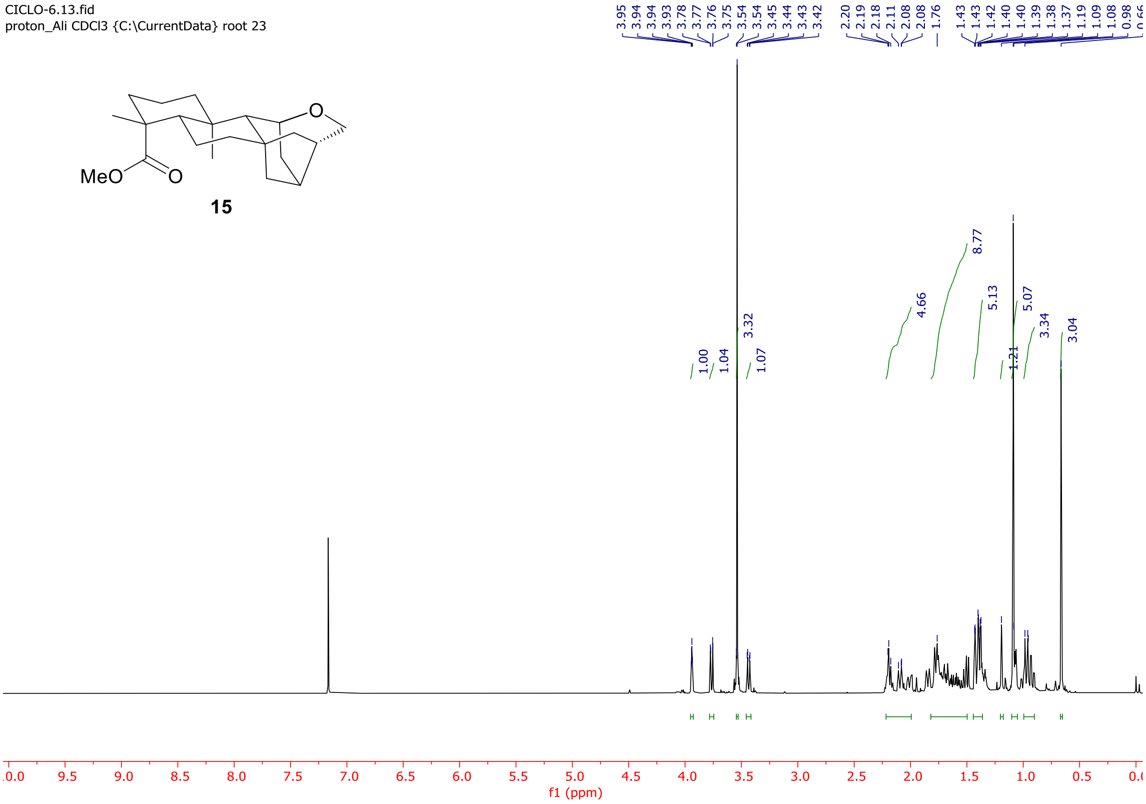

^{13}C{^{1}H} NMR of **15** (125 MHz, $CDCl_3$)

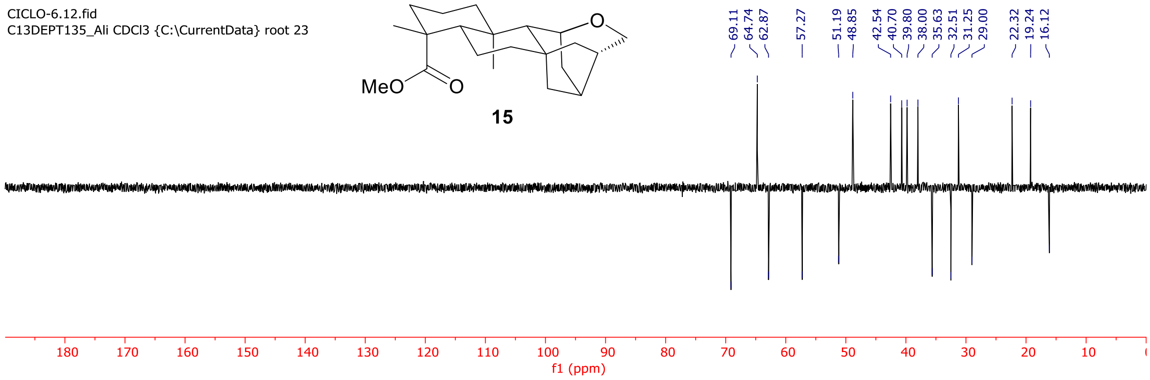

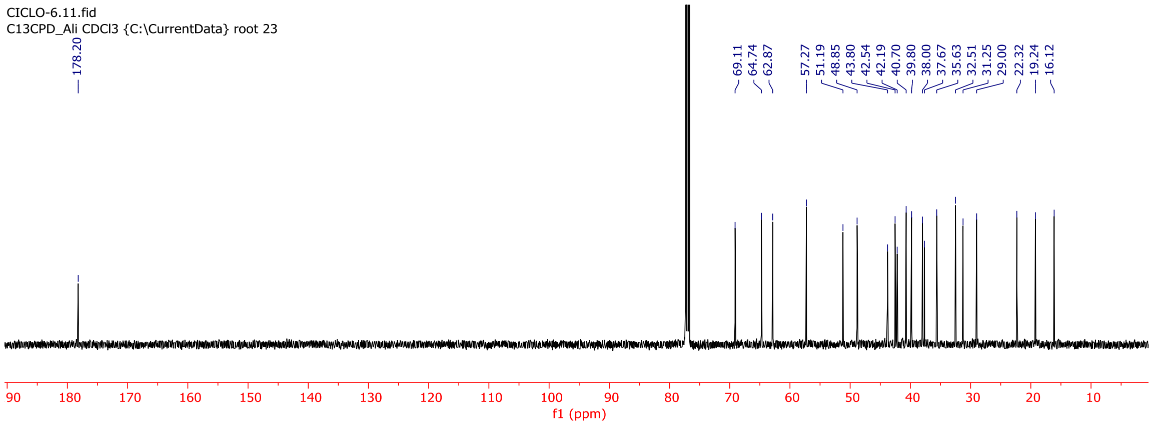

HSQC of 15 (500/125 MHz, $CDCl_3$)

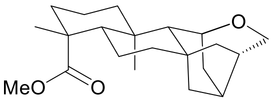

15

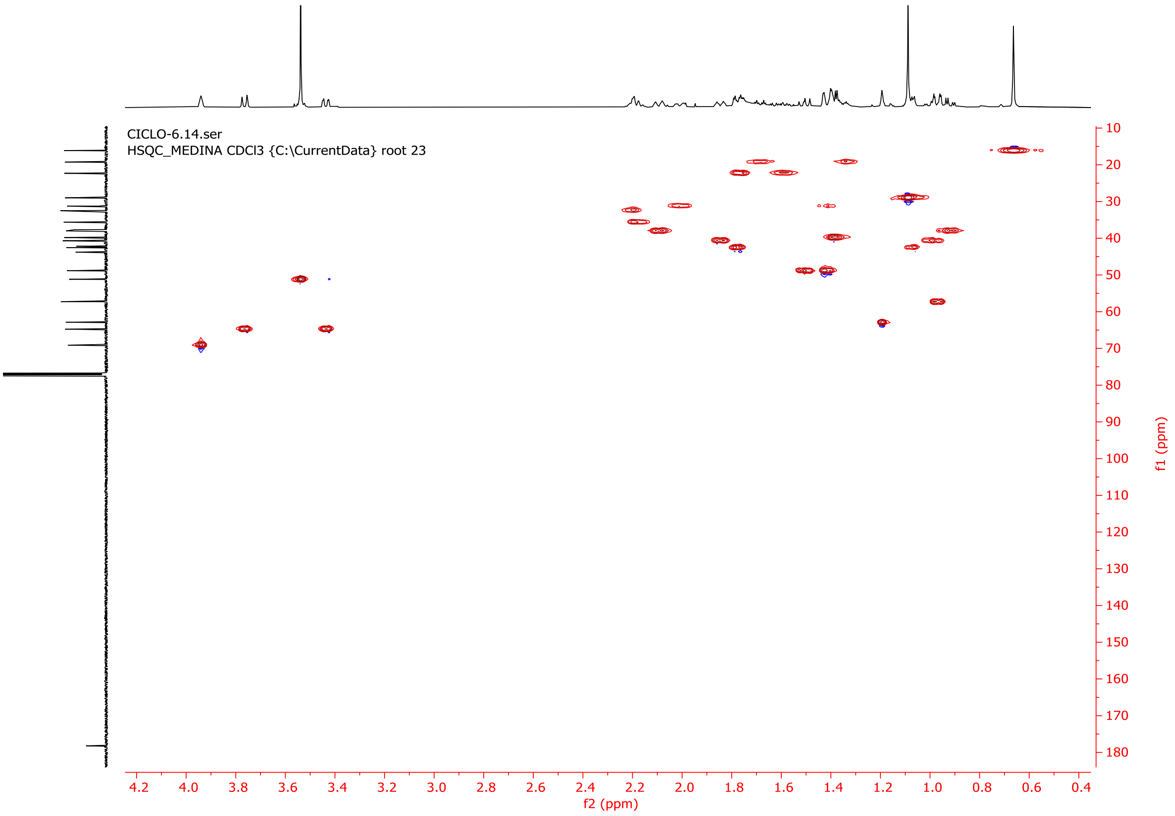

^{1}H NMR of **17** (500 MHz, $CDCl_3$)

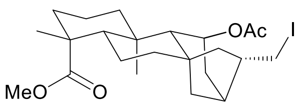

17

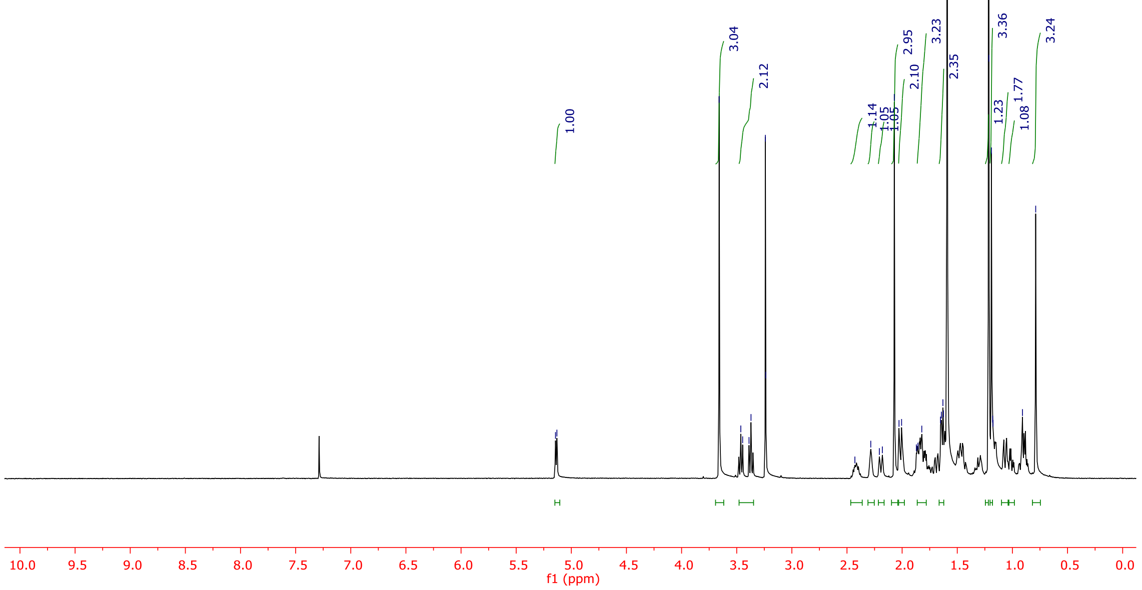

^{13}C{^{1}H} NMR of **17** (125 MHz, $CDCl_3$)

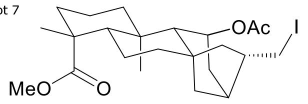

17

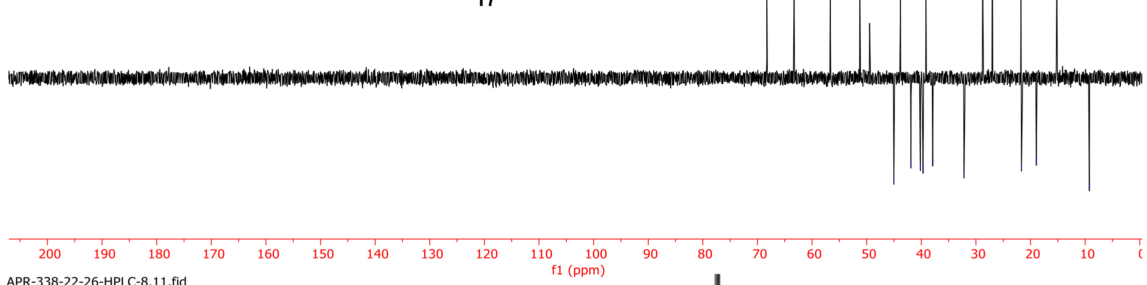

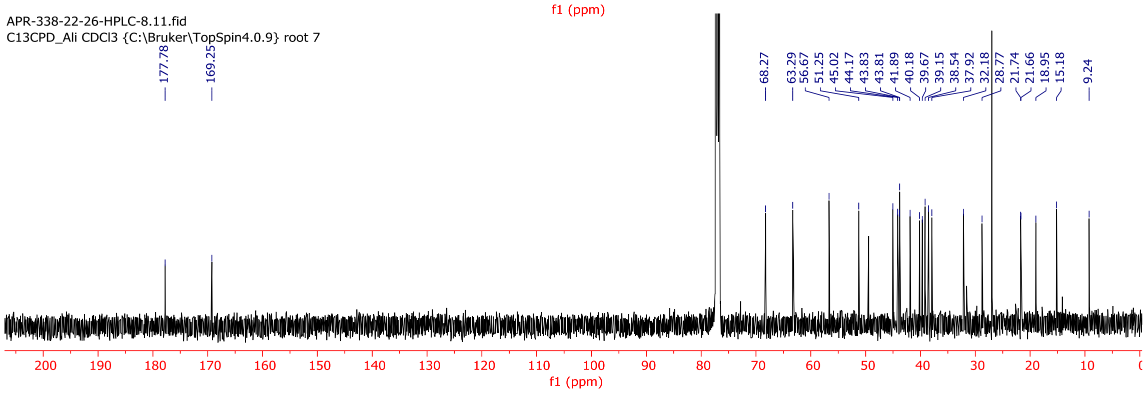

HSQC of 17 (500/125 MHz, $CDCl_3$)

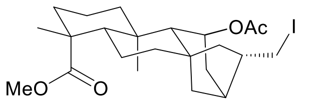

17

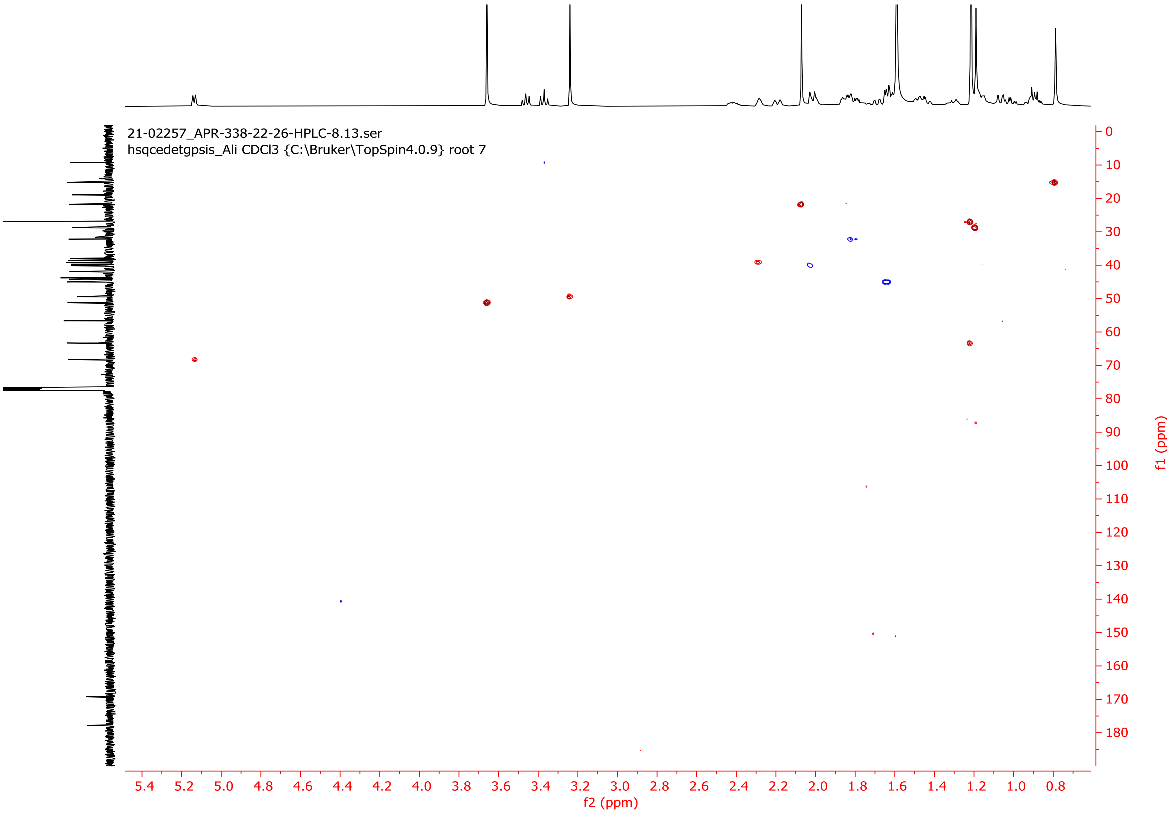

^{1}H NMR of **18** (400 MHz, $CDCl_3$)

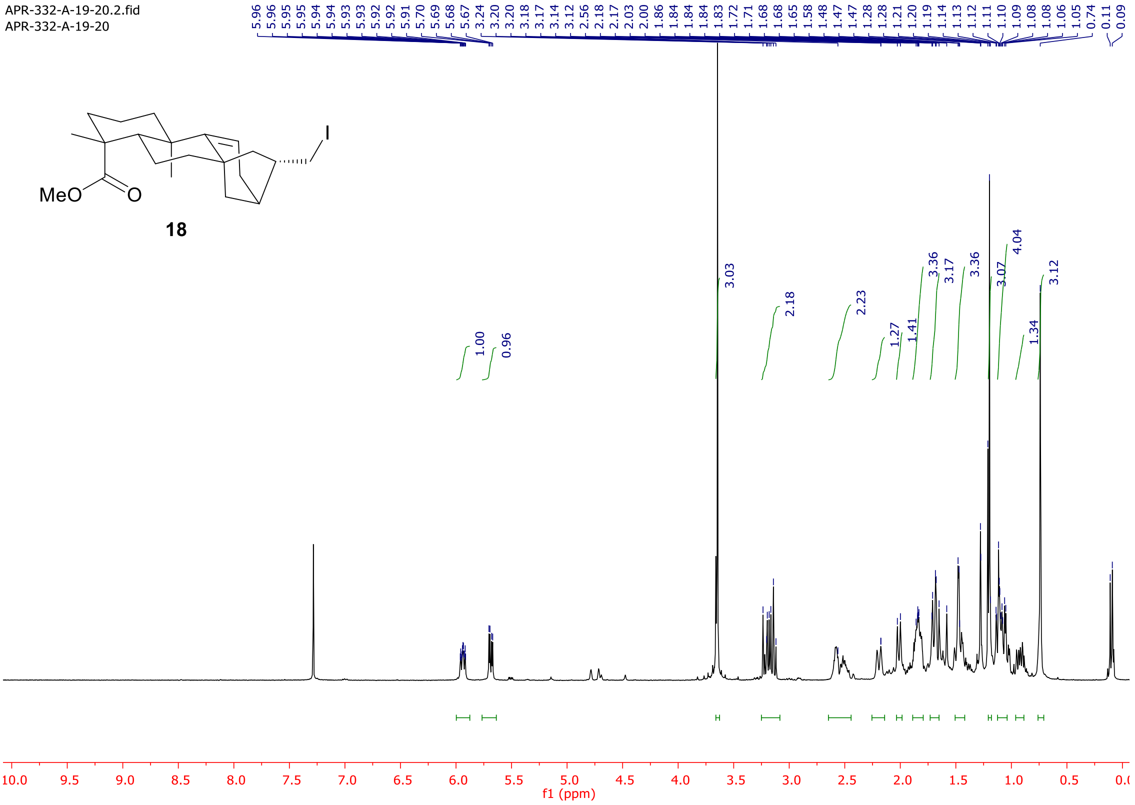

^{13}C{^{1}H} NMR of **18** (100 MHz, $CDCl_3$)

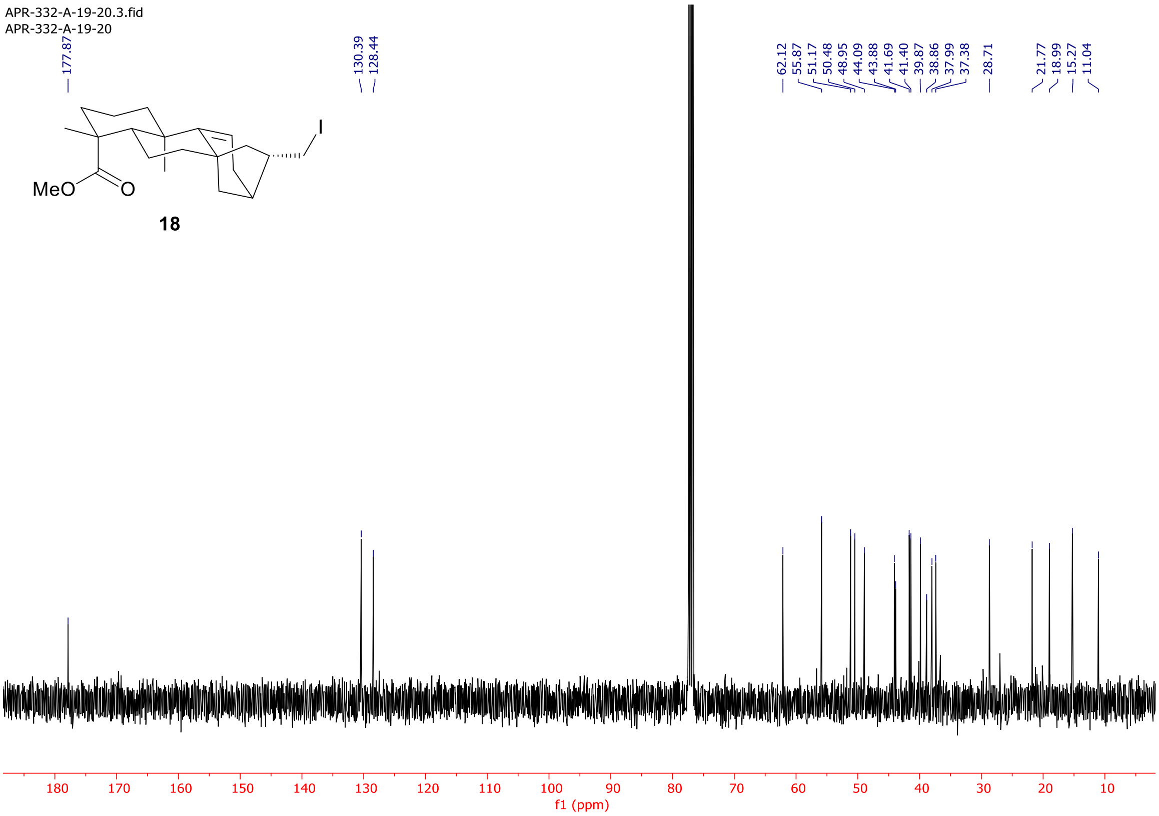

HSQC of 18 (400/100 MHz, $CDCl_3$)

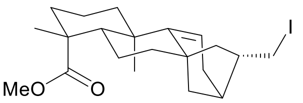

18

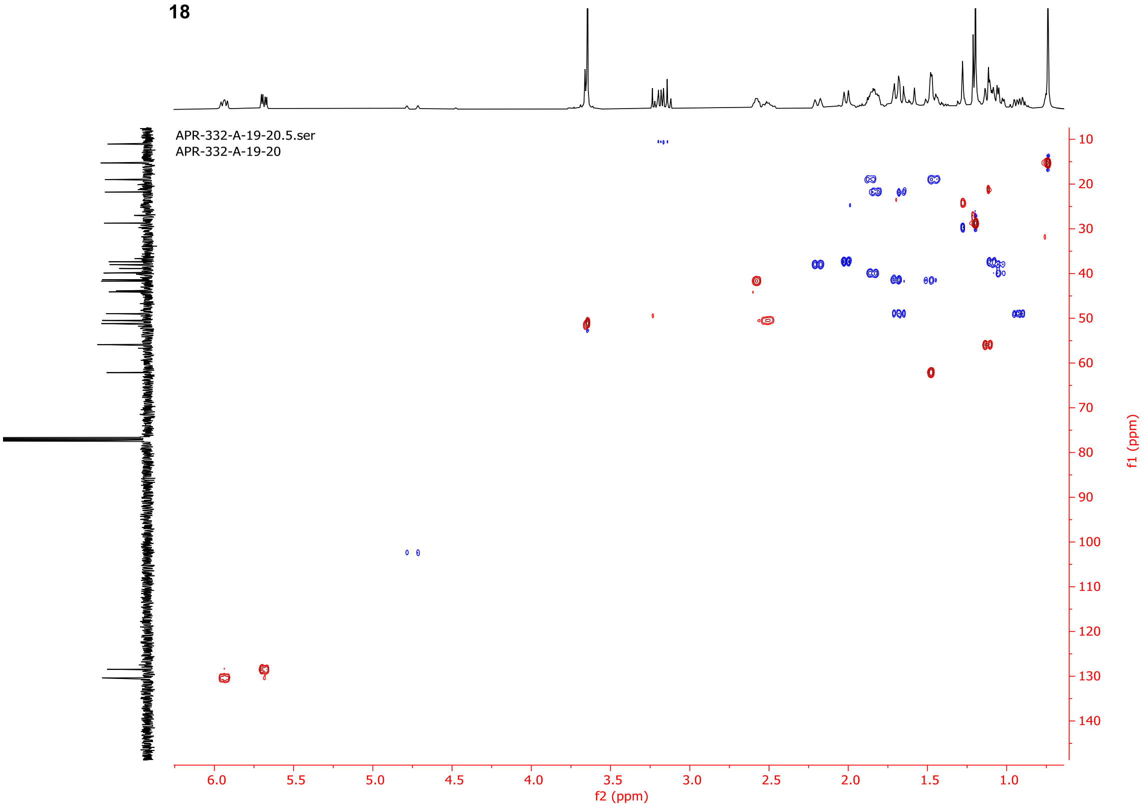

^{1}H NMR of **19** (500 MHz, $CDCl_3$)

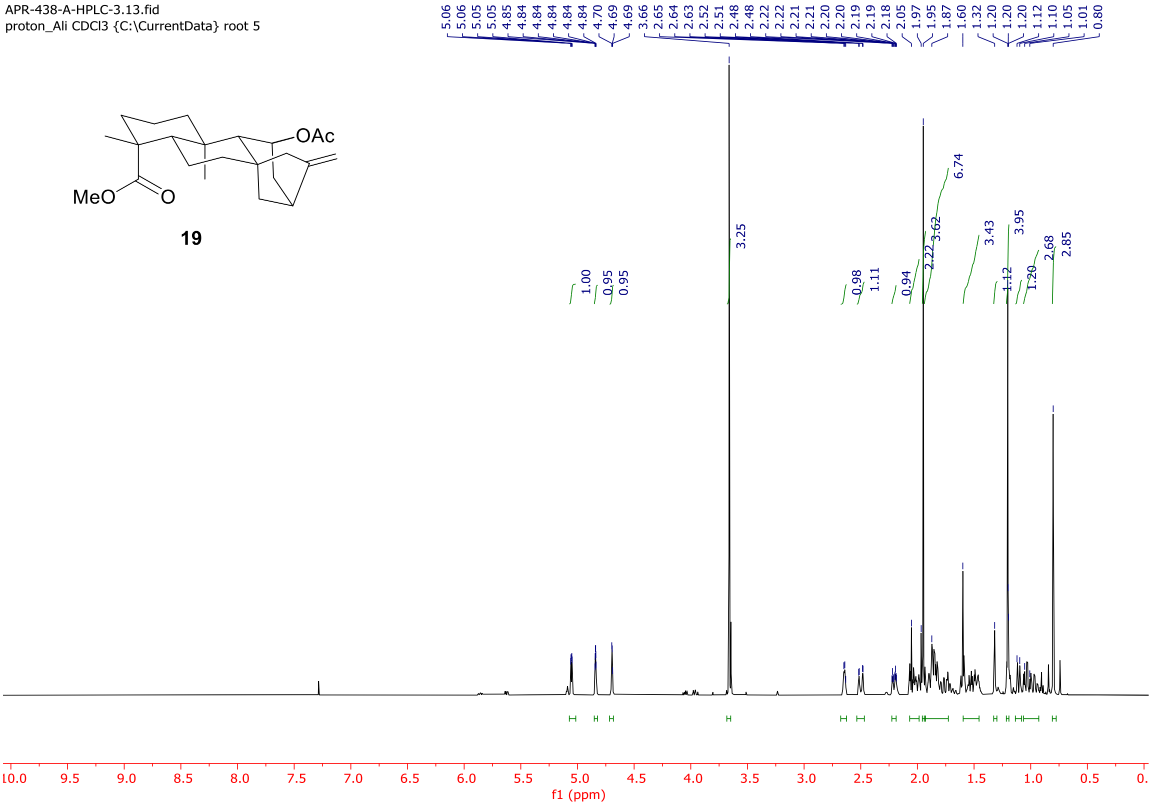

$^{13}C\{^{1}H\}$ NMR of **19** (125 MHz, $CDCl_3$)

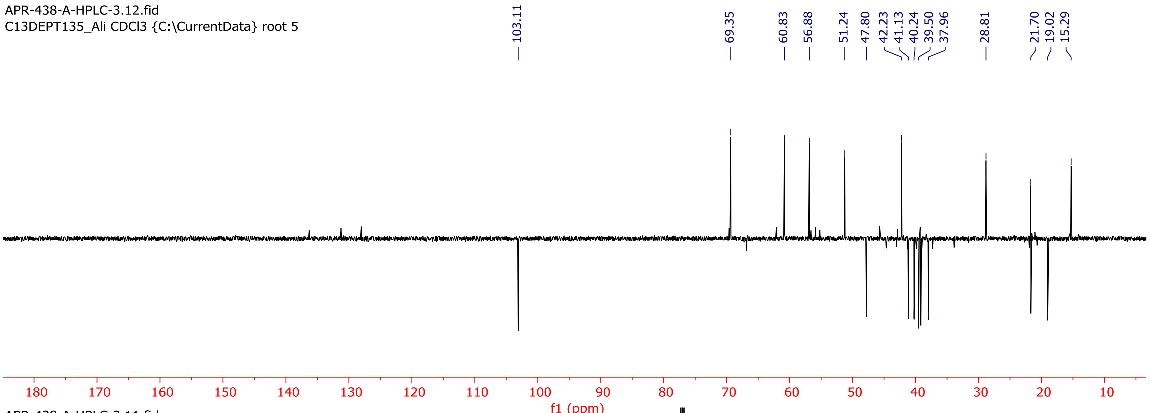

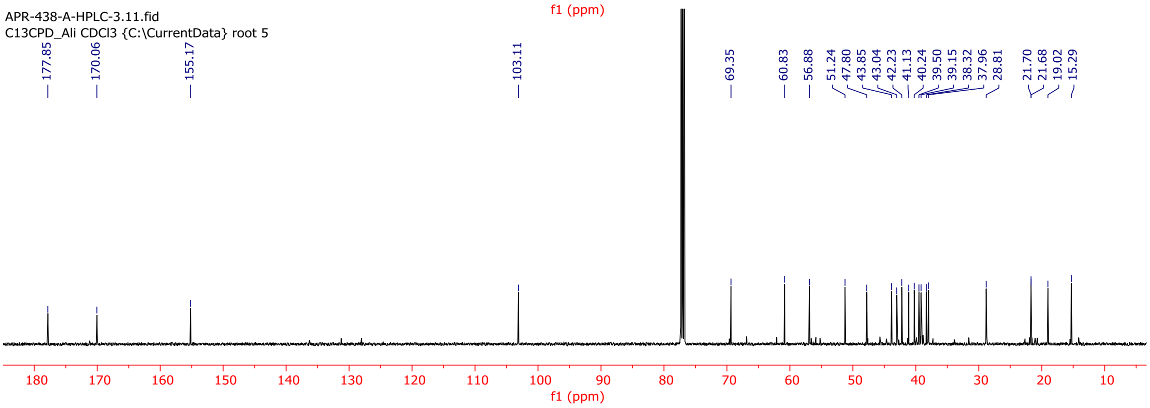

HSQC of 19 (500/125 MHz, $CDCl_3$)

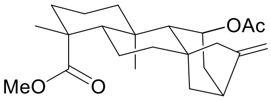

19

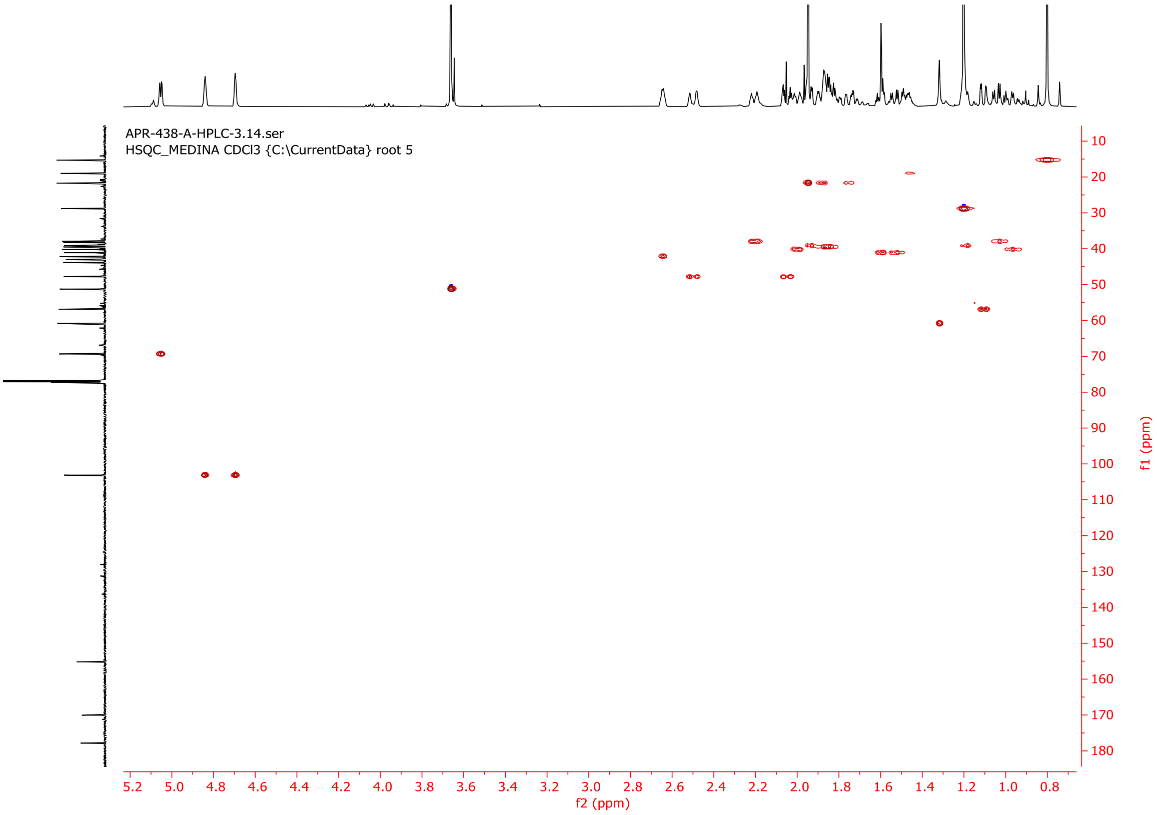

^{1}H NMR of **20** (600 MHz, $CDCl_3$)

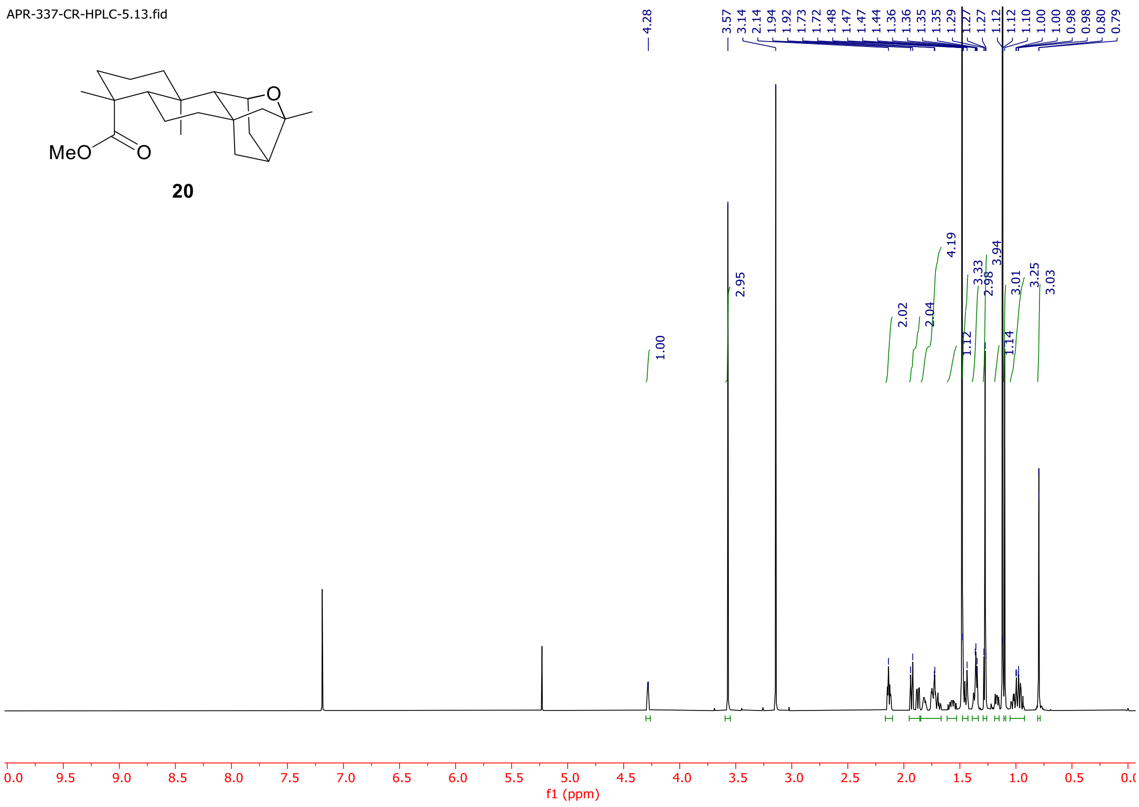

^{13}C{^{1}H} NMR of **20** (150 MHz, $CDCl_3$)

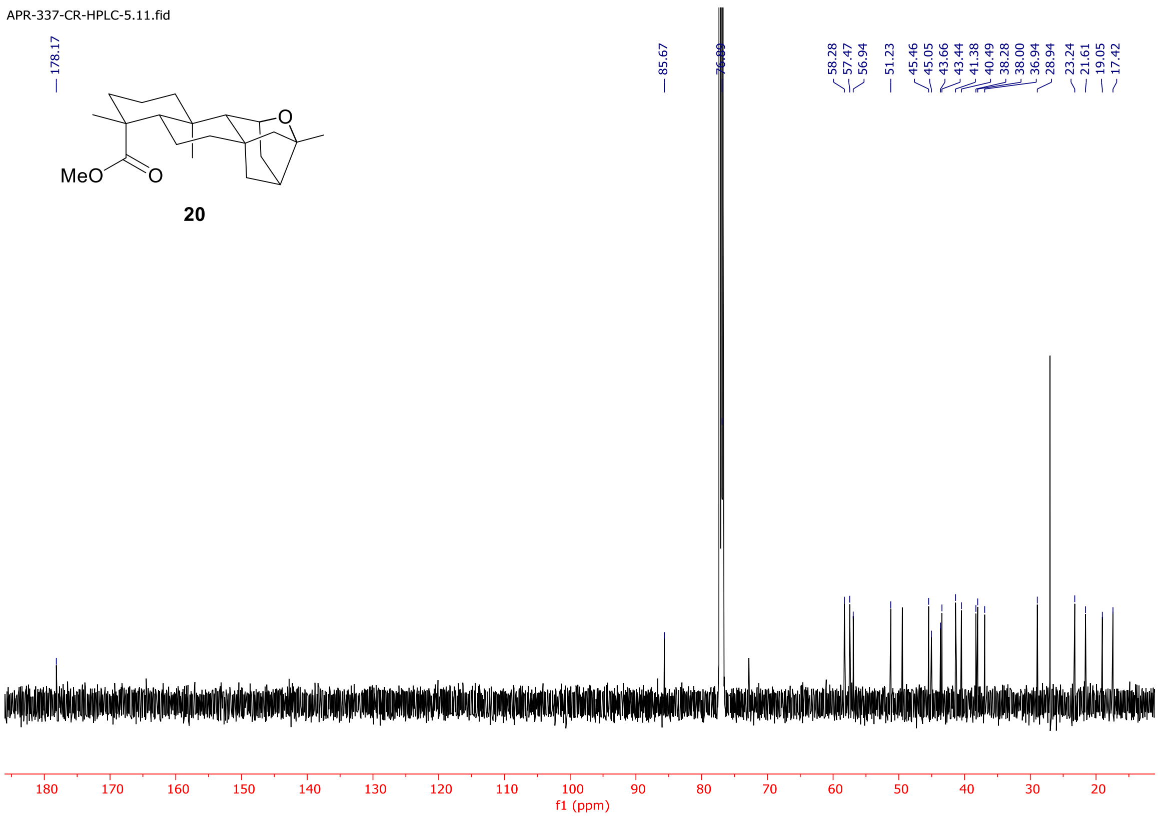

HSQC of 20 (600/150 MHz, $CDCl_3$)

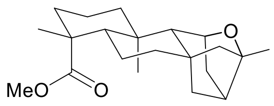

20

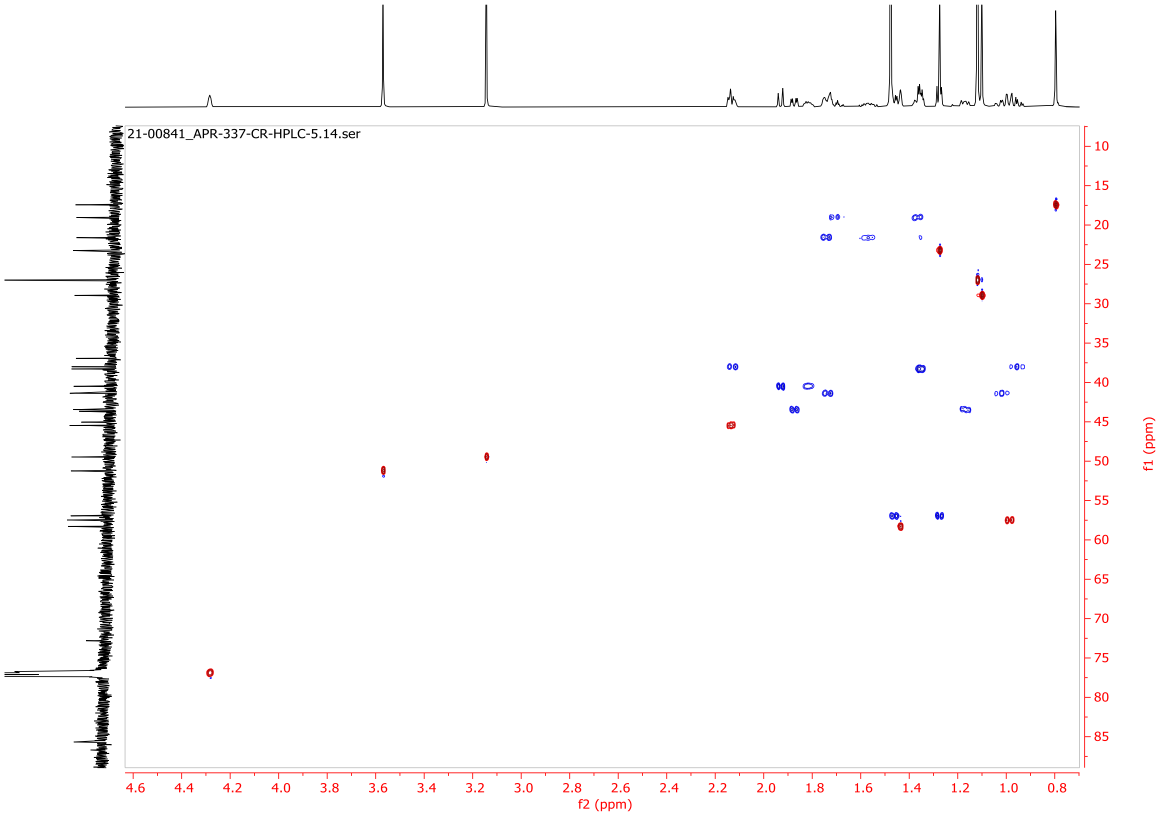

^{1}H NMR of **21** (600 MHz, $CDCl_3$)

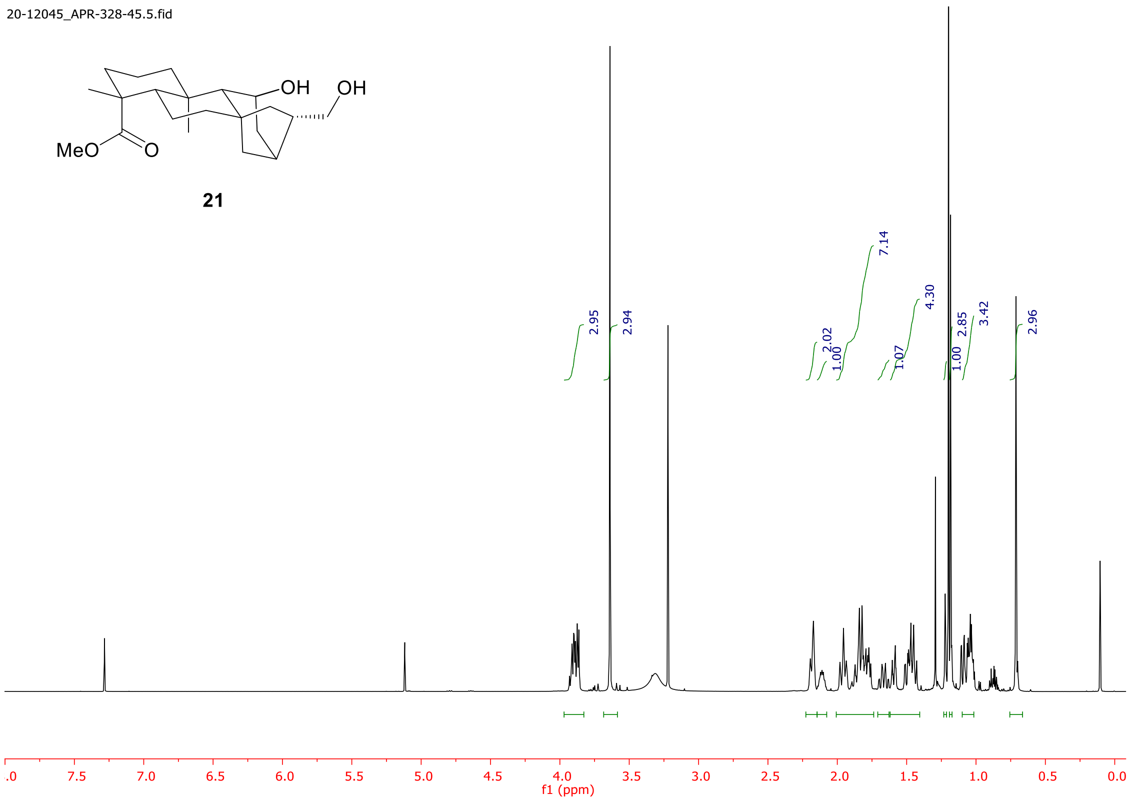

$^{13}C\{^{1}H\}$ NMR of **21** (150 MHz, $CDCl_3$)

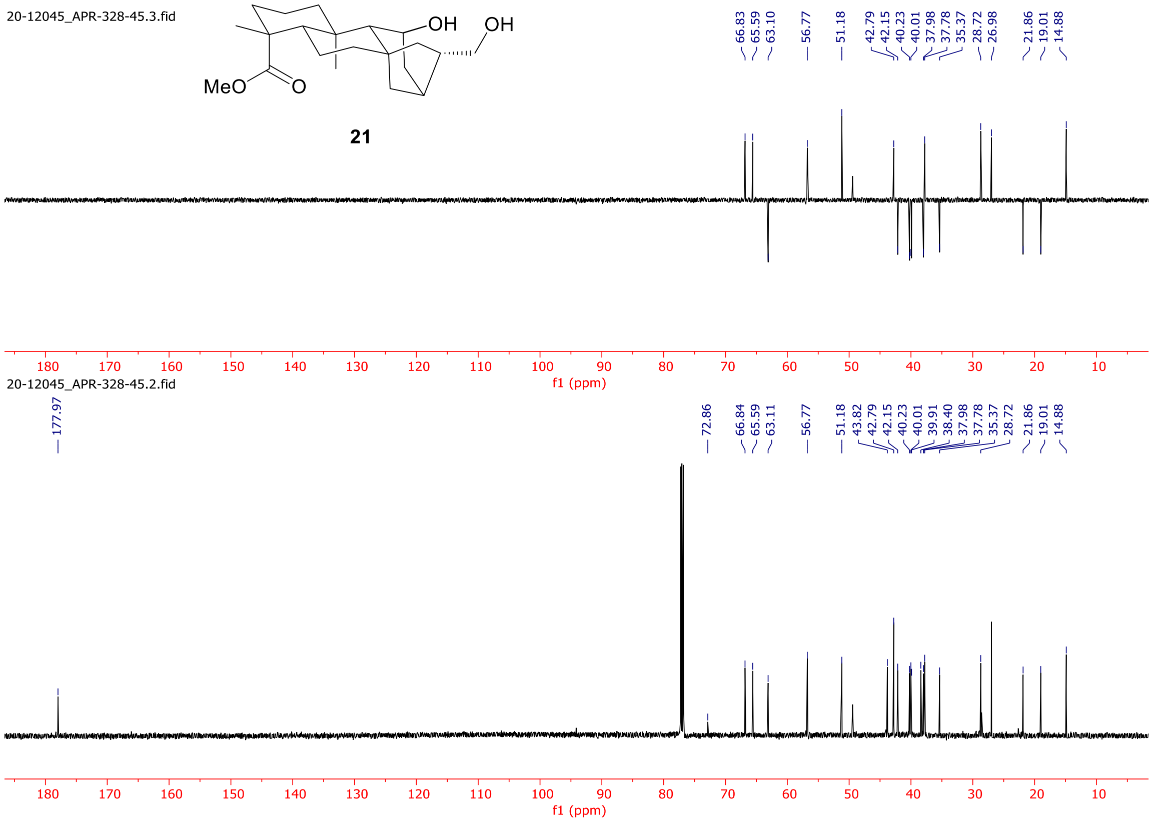

HSQC of 21 (600/150 MHz, $CDCl_3$)

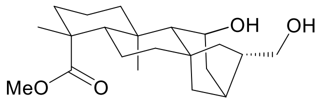

21

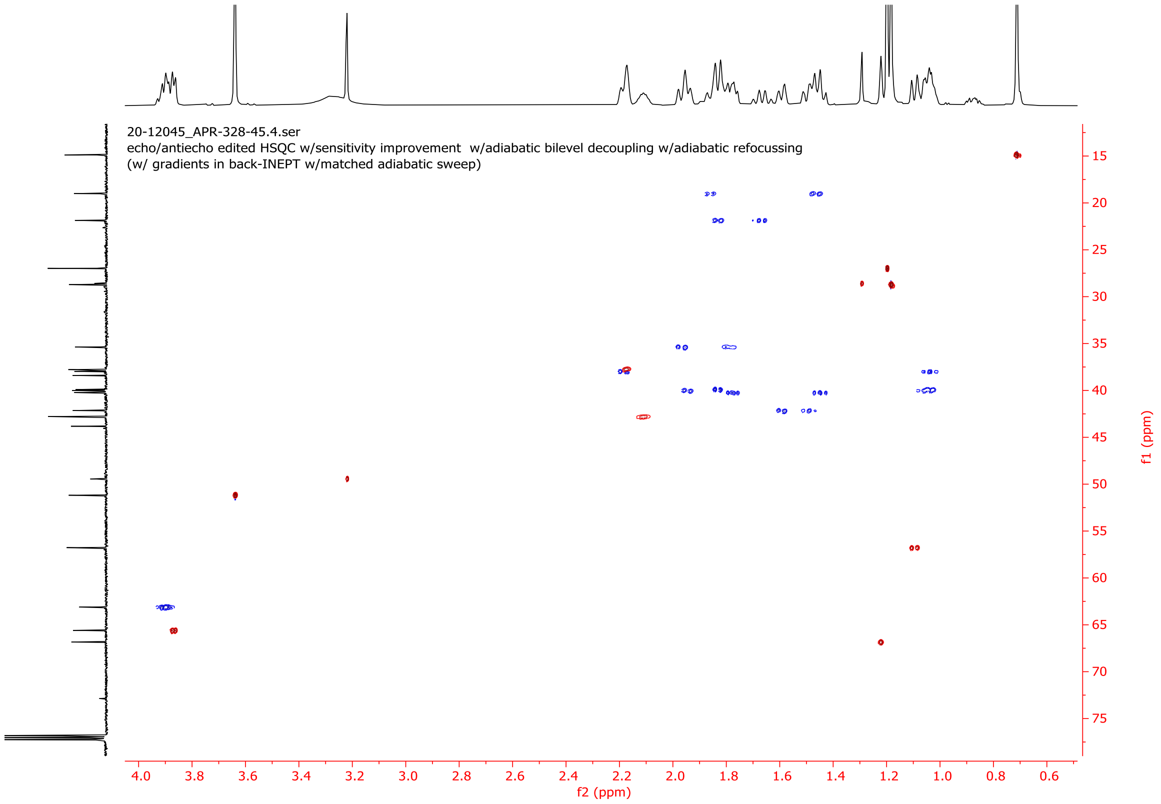

^{1}H NMR of **22** (500 MHz, $CDCl_3$)

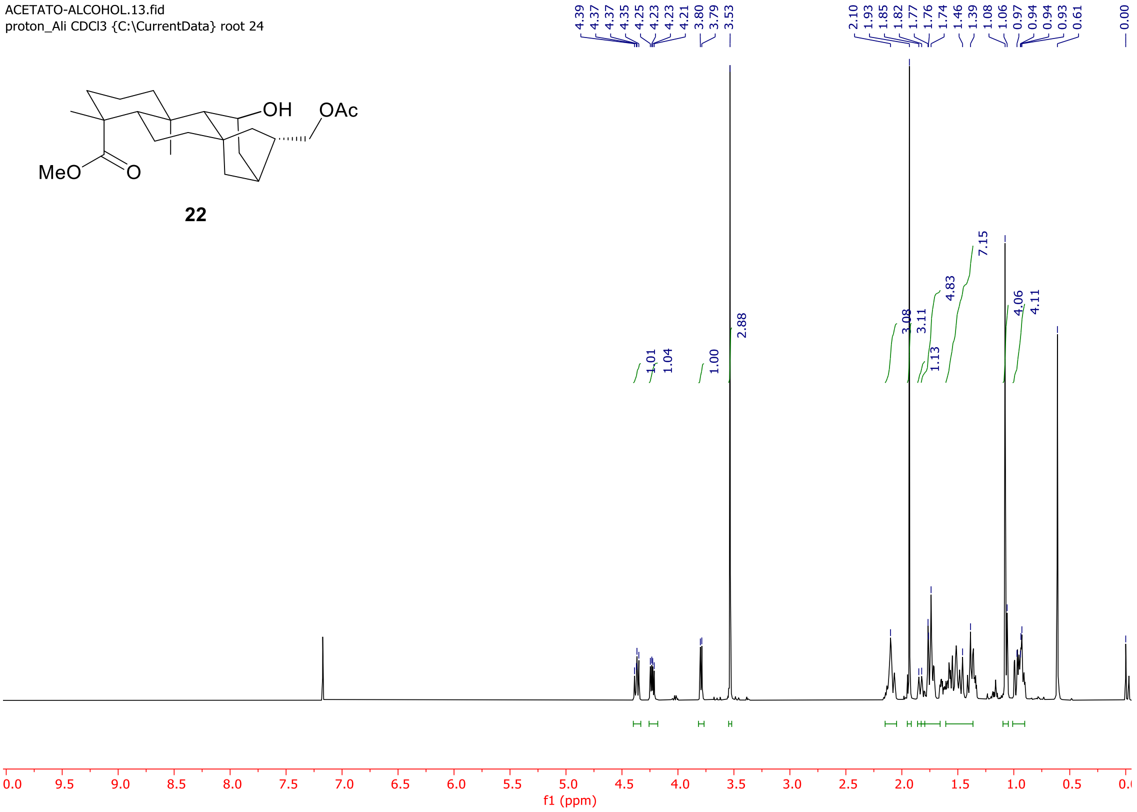

$^{13}C\{^{1}H\}$ NMR of **22** (125 MHz, $CDCl_3$)

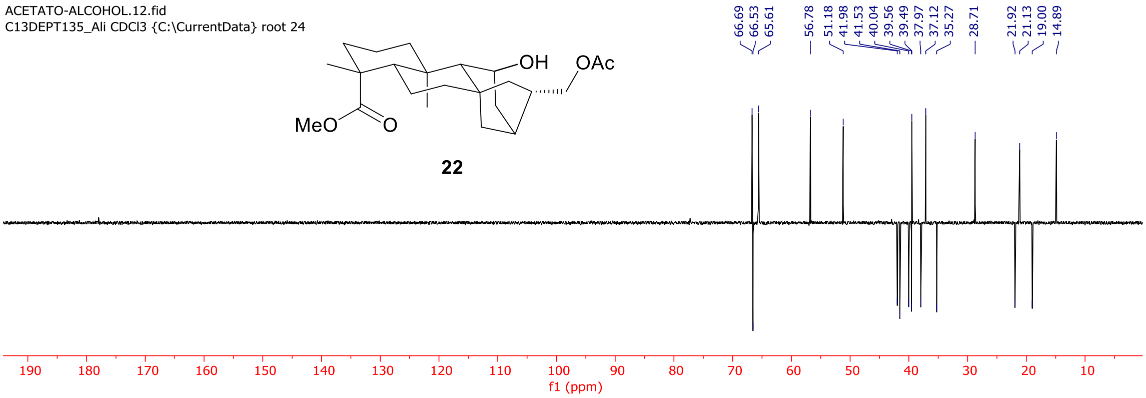

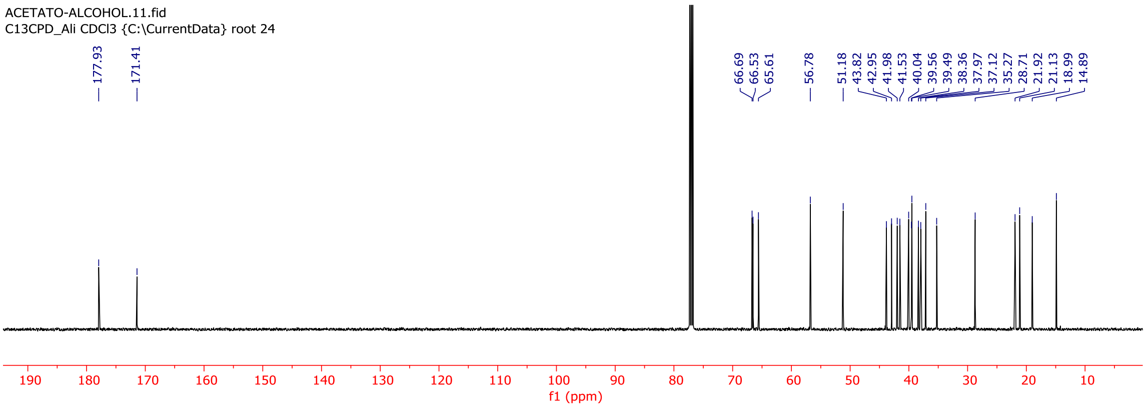

HSQC of 22 (500/125 MHz, $CDCl_3$)

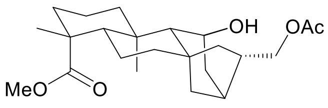

22

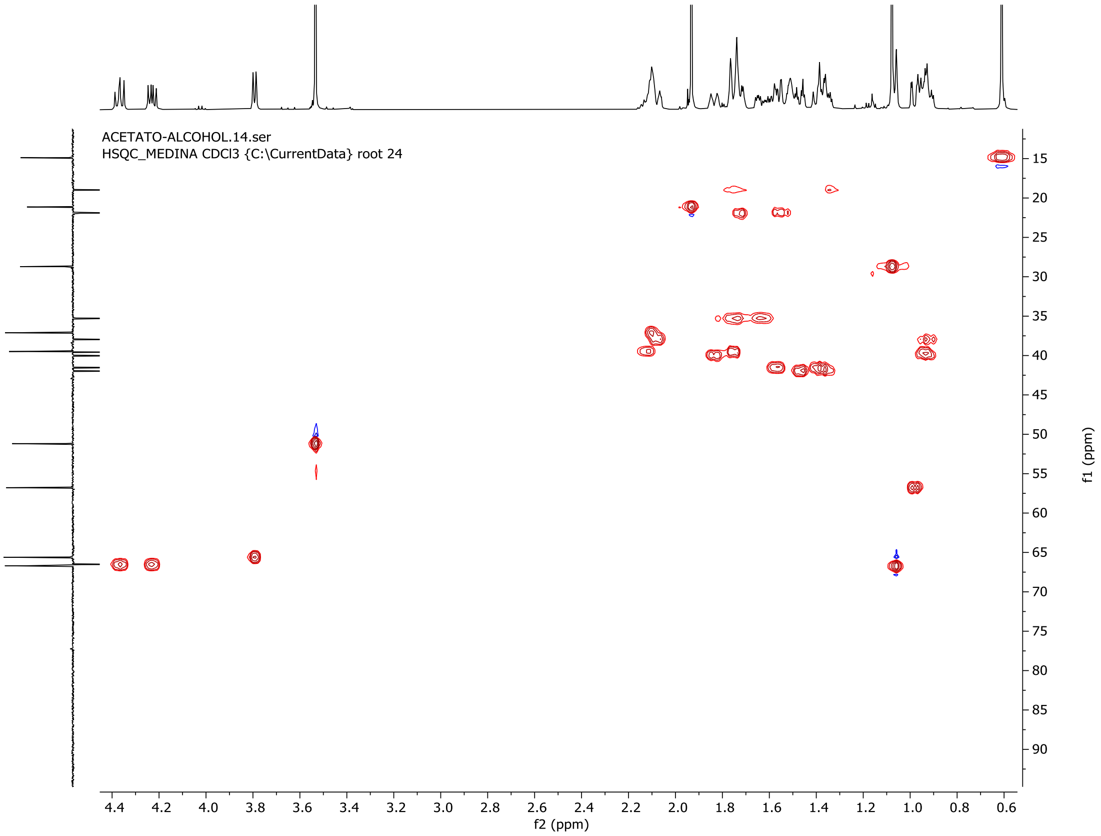

^{1}H NMR of **23** (400 MHz, $CDCl_3$)

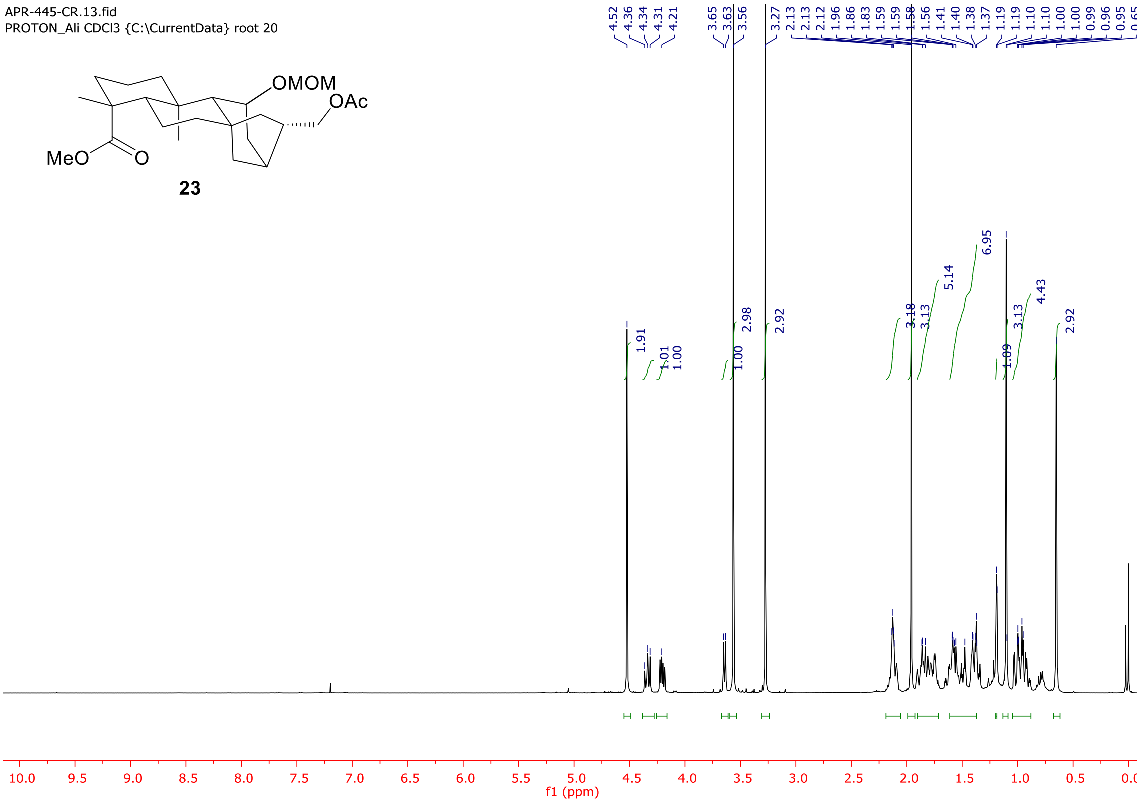

$^{13}C\{^{1}H\}$ NMR of **23** (100 MHz, $CDCl_3$)

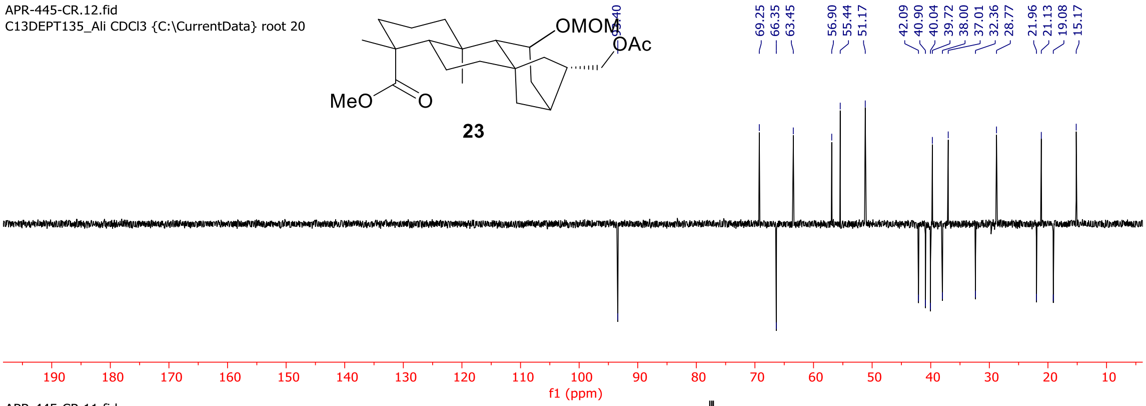

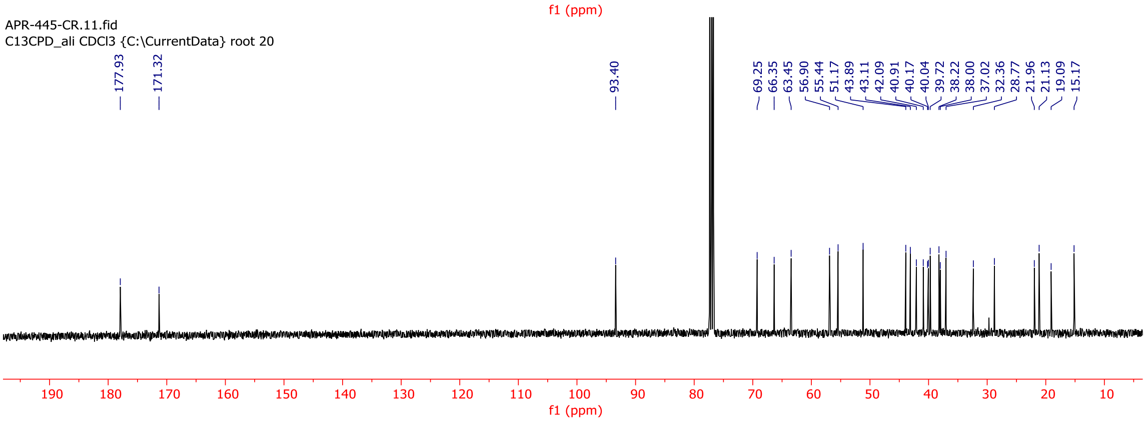

HSQC of 23 (400/100 MHz, $CDCl_3$)

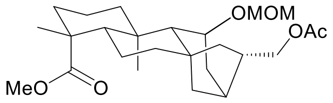

23

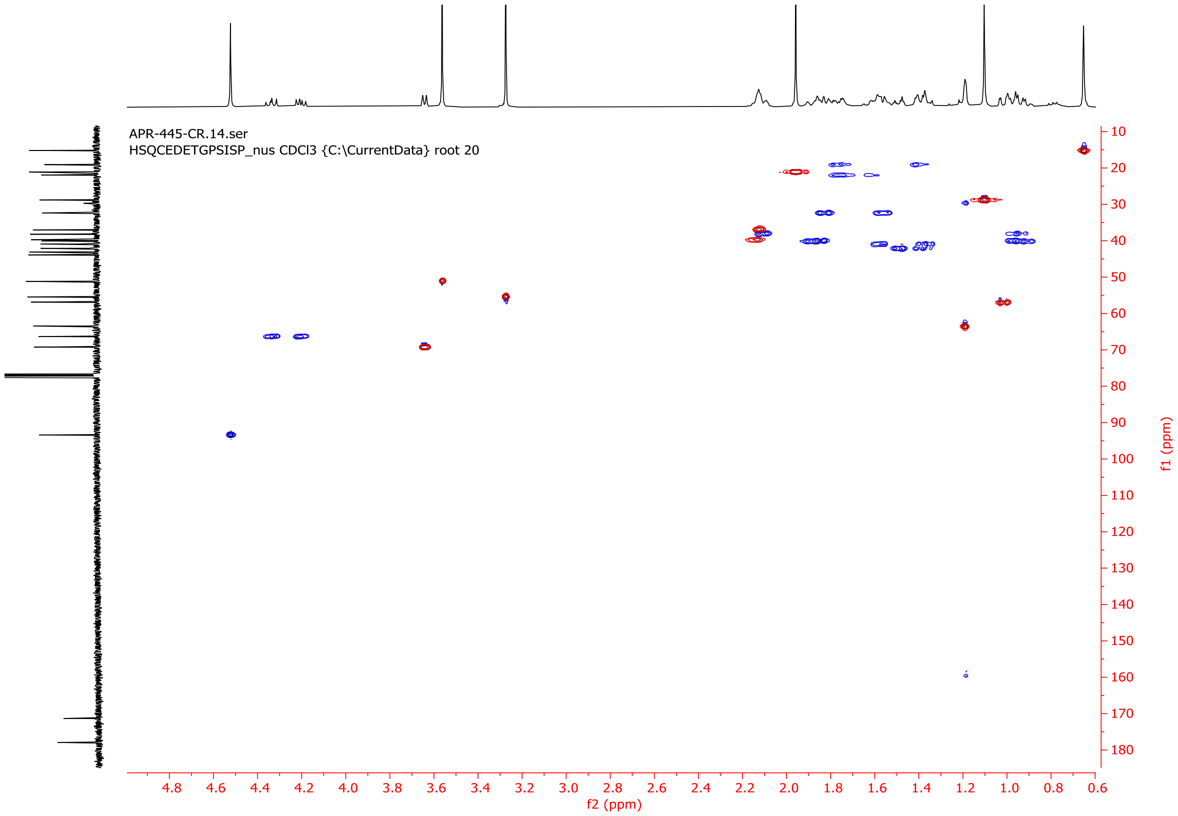

^{1}H NMR of **24** (400 MHz, $CDCl_3$)

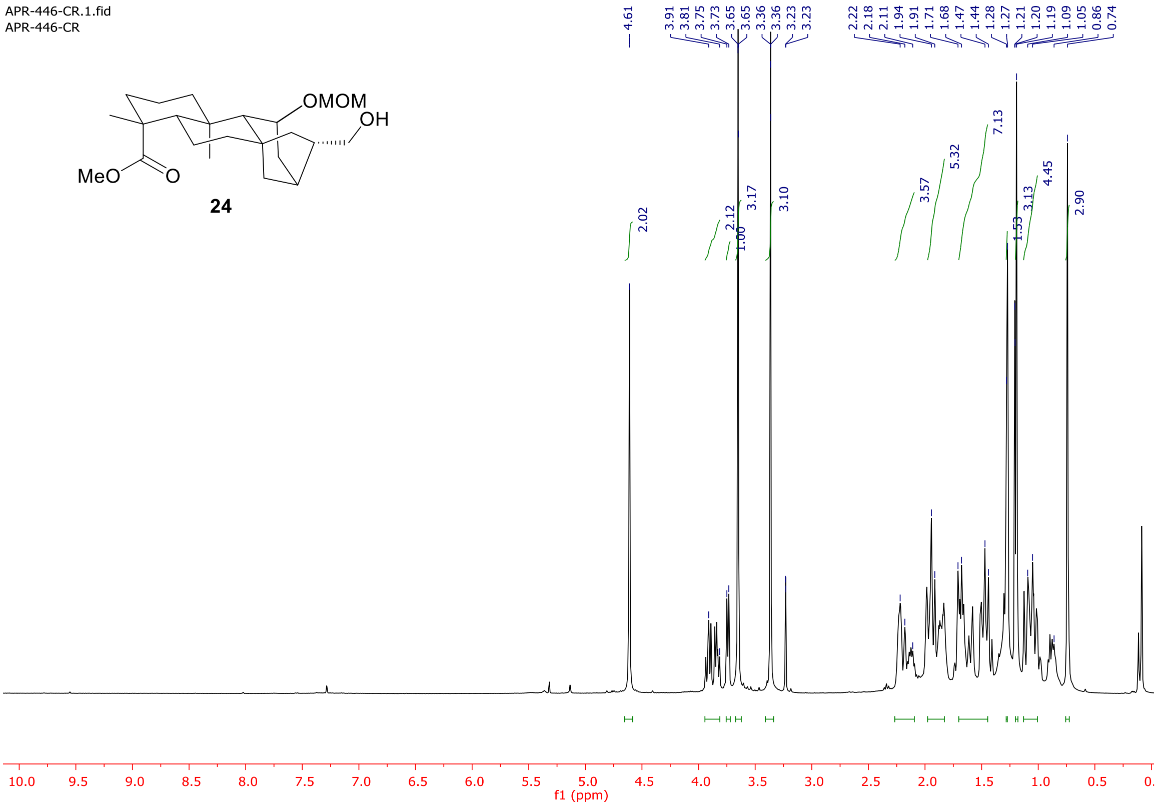

^{13}C{^{1}H} NMR of **24** (100 MHz, $CDCl_3$)

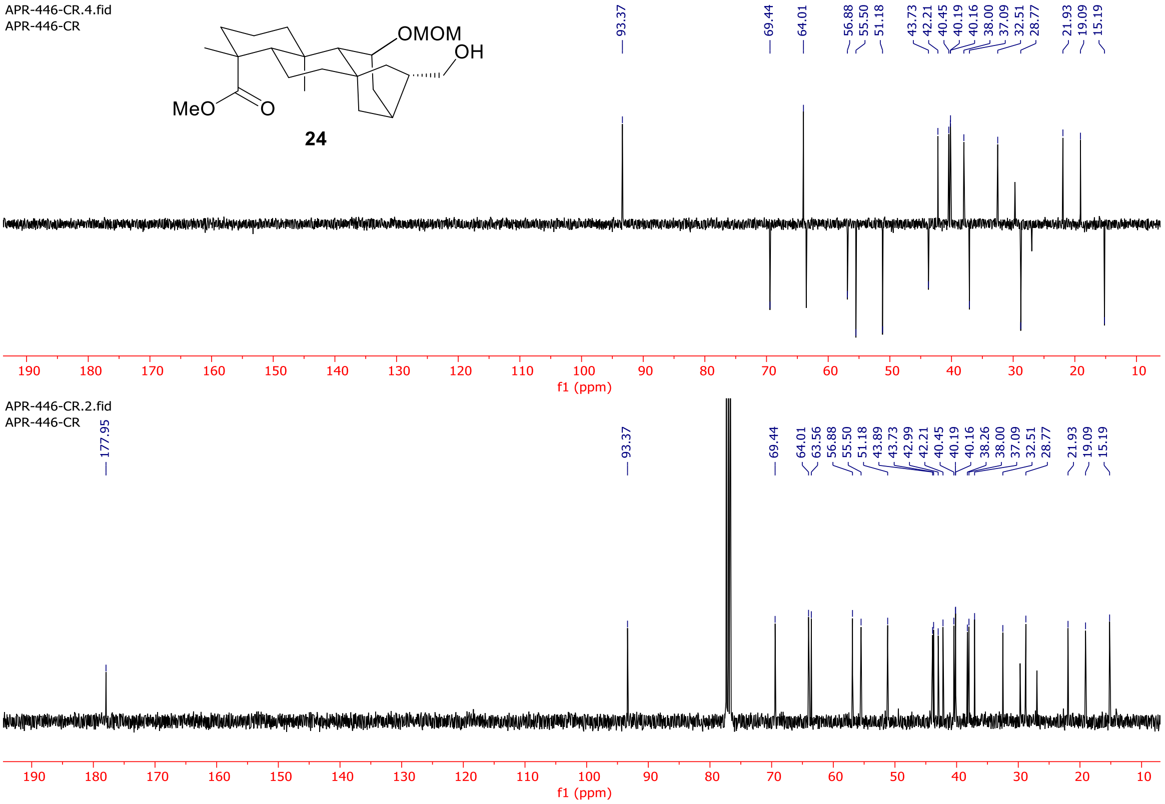

HSQC of 24 (400/100 MHz, $CDCl_3$)

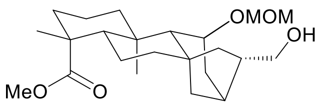

24

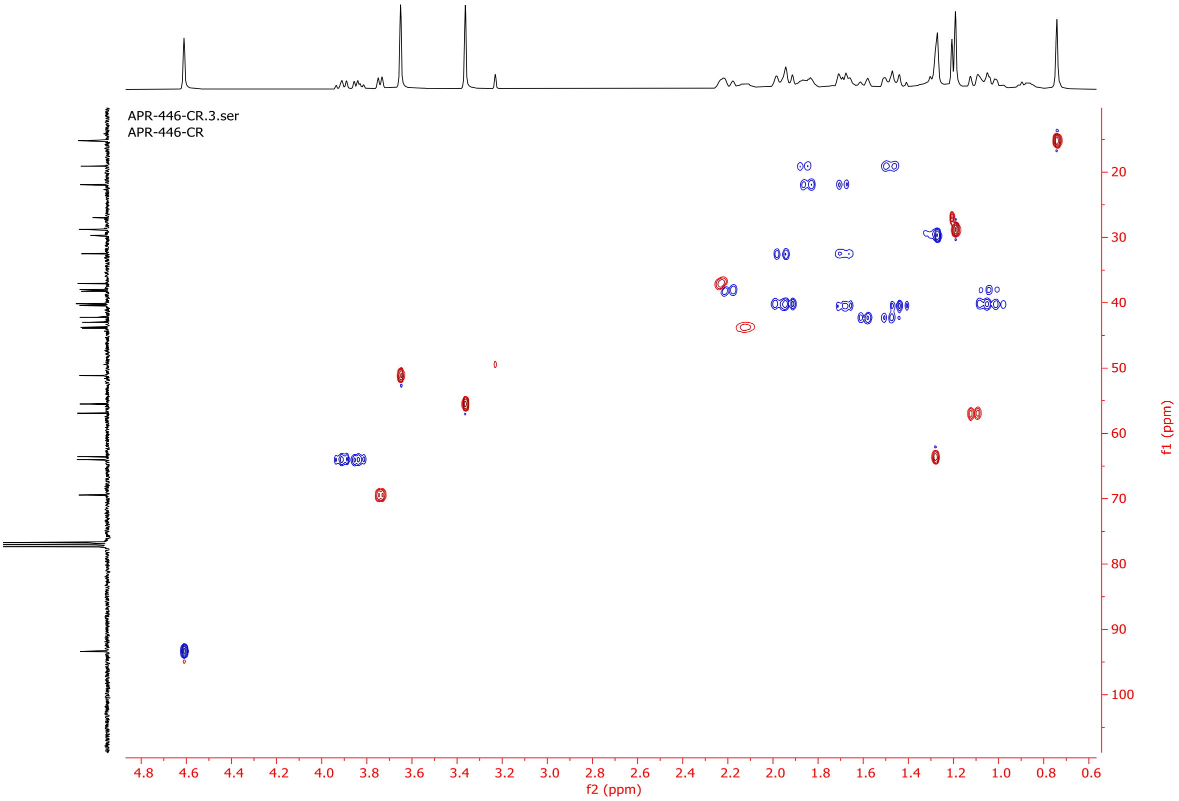

^1H NMR of **25** (500 MHz, $CDCl_3$)

MOMO-DOBLEENLACE.10.fid
Barrero10mg
proton_Ali CDCl3 {C:\CurrentData} root 1

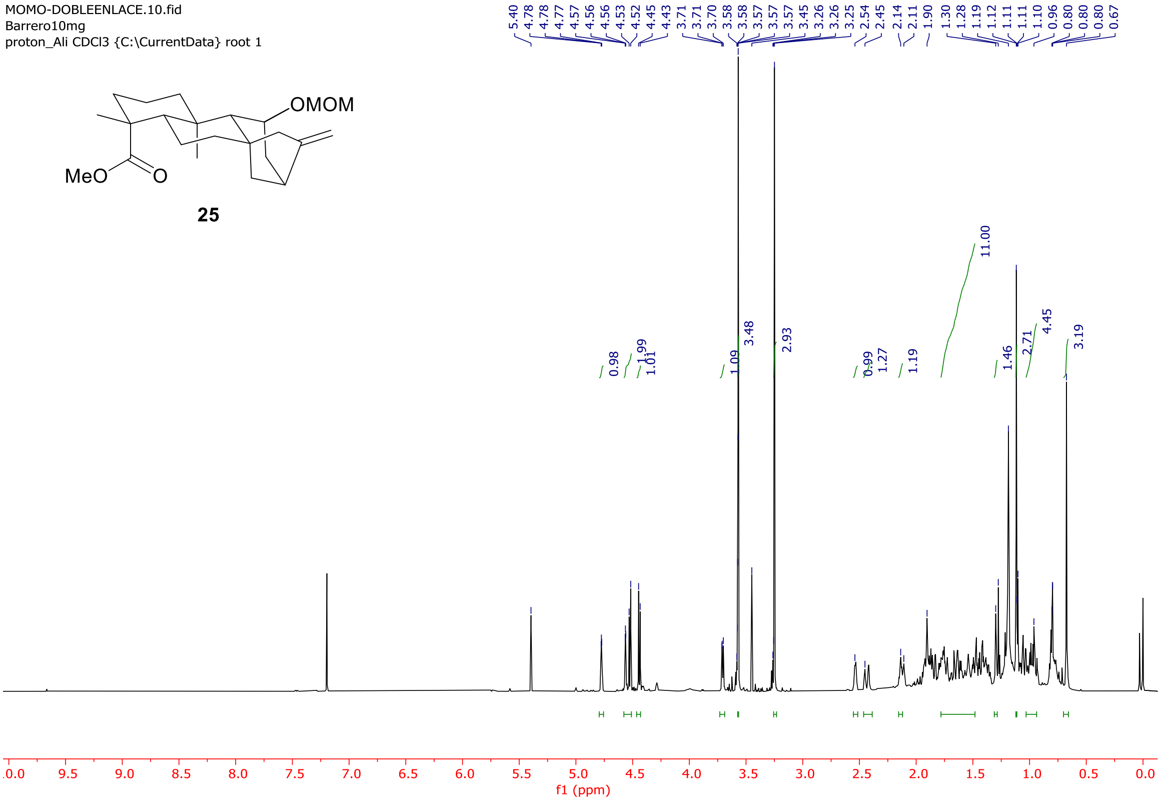

$^{13}C\{^1H\}$ NMR of **25** (125 MHz, $CDCl_3$)

MOMO-DOBLEENLACE.12.fid
C13DEPT135_Ali CDCl3 {C:\CurrentData} root 1

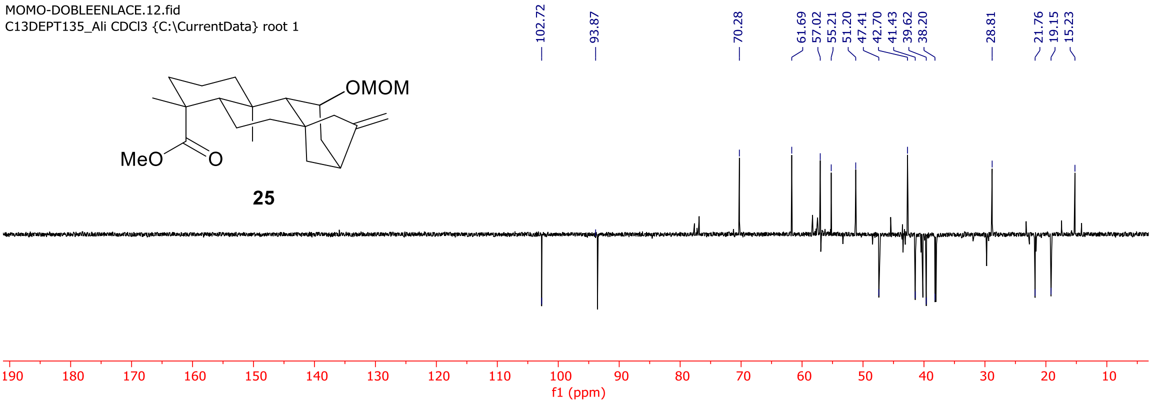

MOMO-DOBLEENLACE.11.fid
C13CPD_Ali CDCl3 {C:\CurrentData} root 1

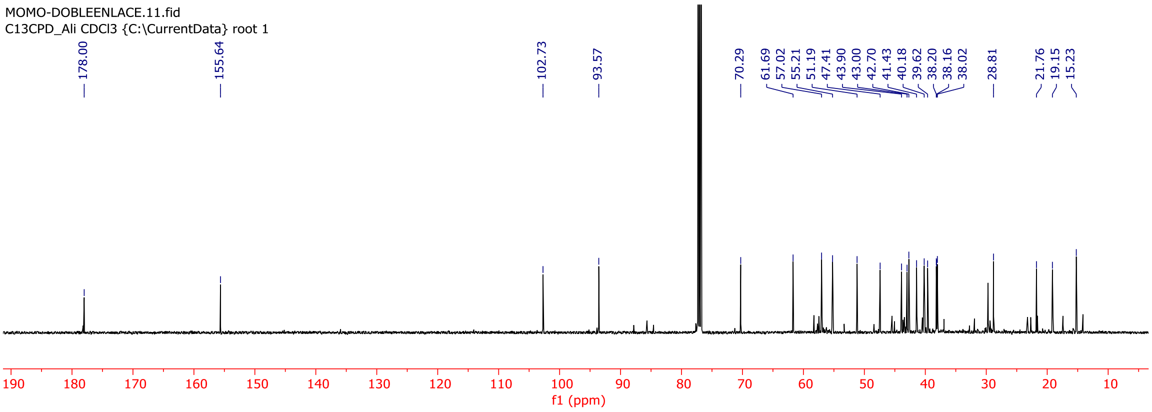

HSQC of 25 (500/125 MHz, $CDCl_3$)

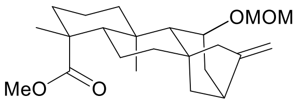

25

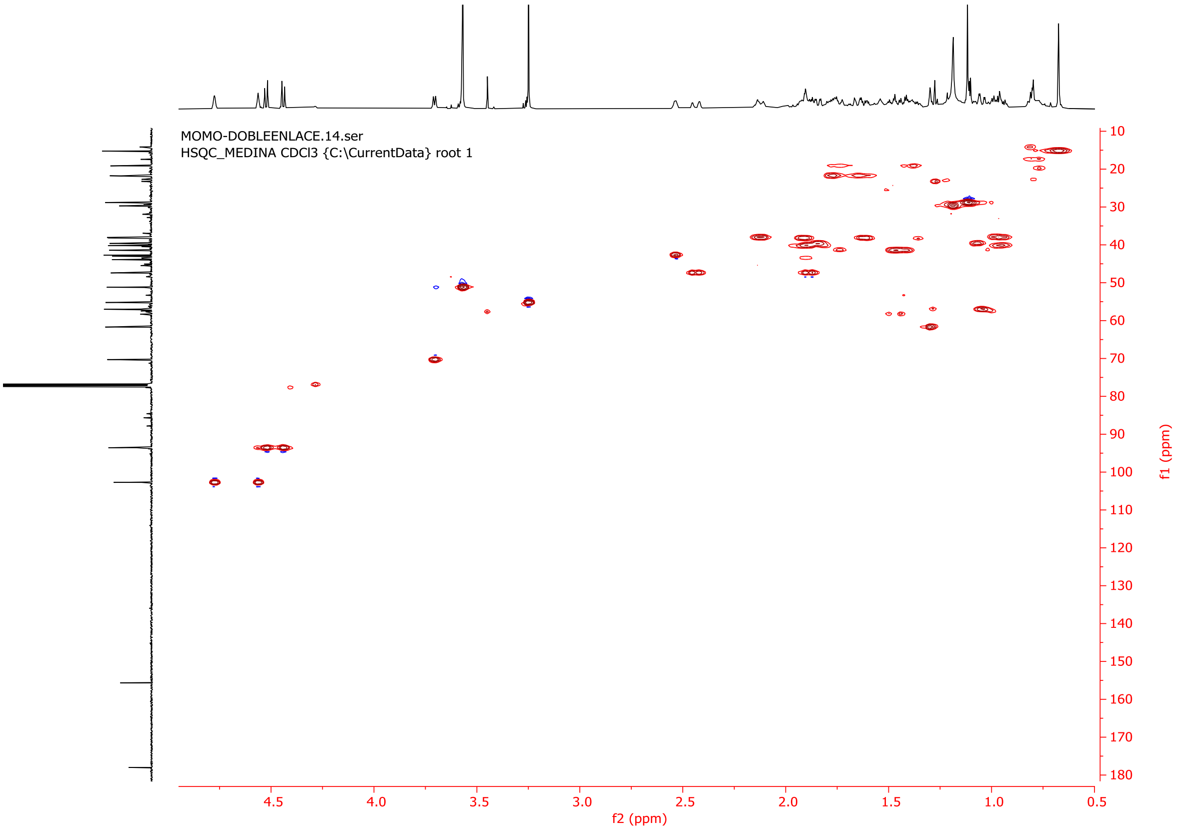

^{1}H NMR of **6** (500 MHz, $CDCl_3$)

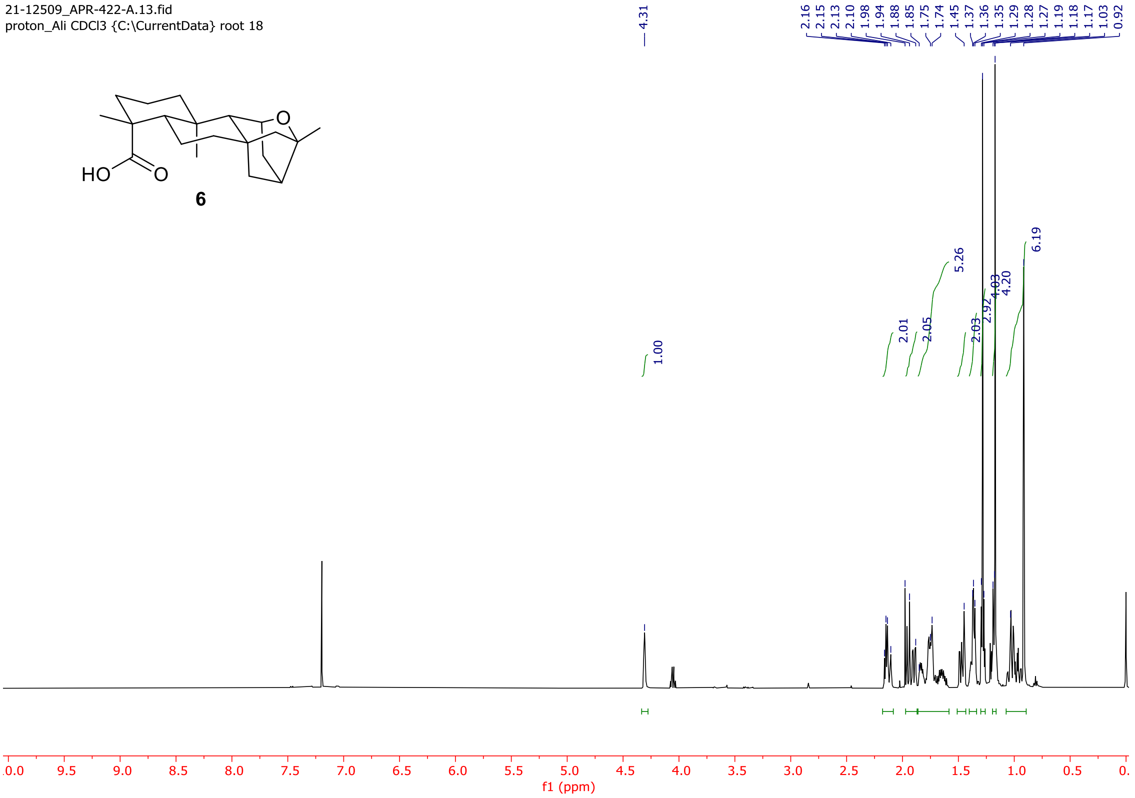

^{13}C{^{1}H} NMR of **6** (125 MHz, $CDCl_3$)

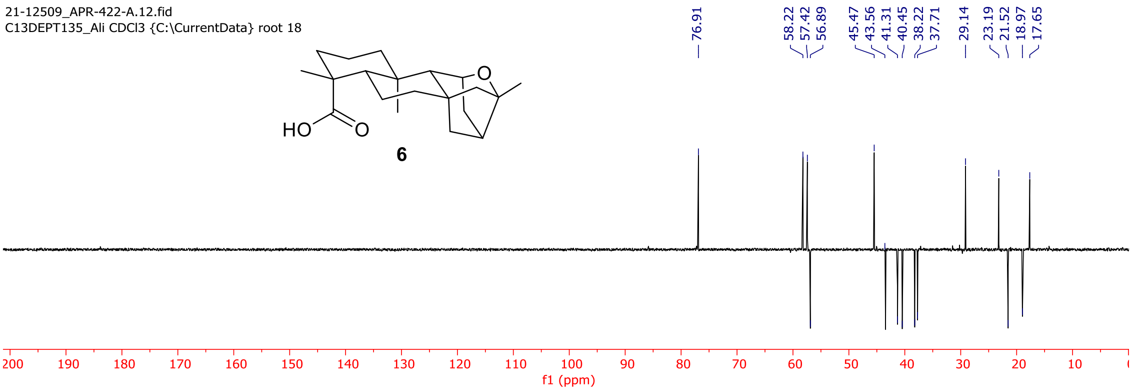

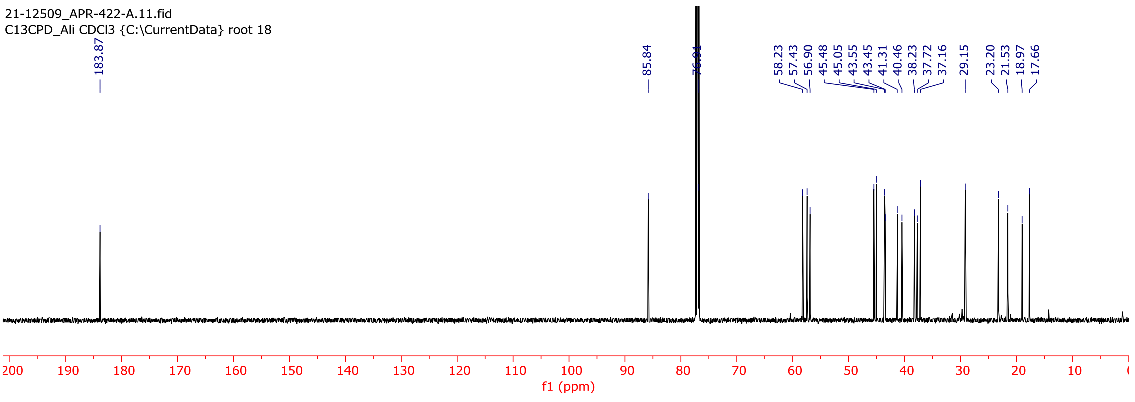

HSQC of 6 (500/125 MHz, $CDCl_3$)

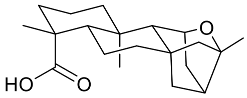

6

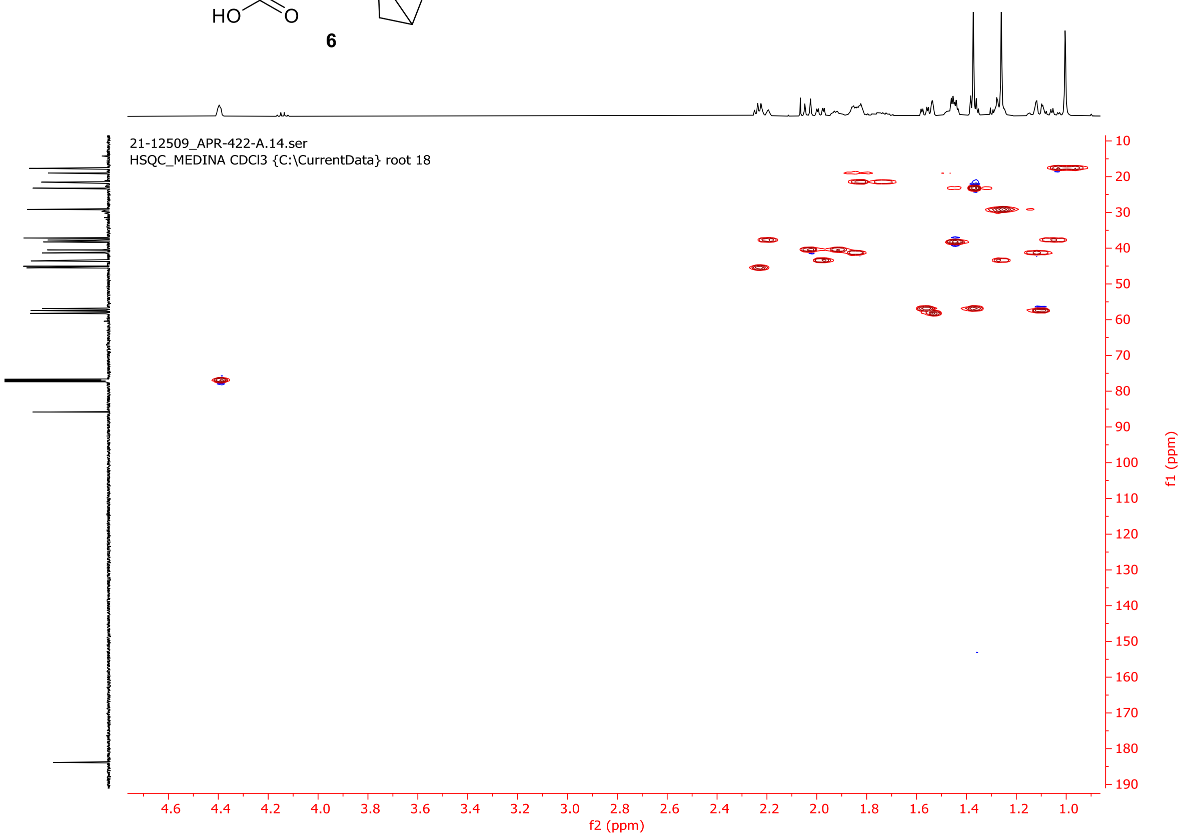

^{1}H NMR of **5** (500 MHz, $CDCl_3$)

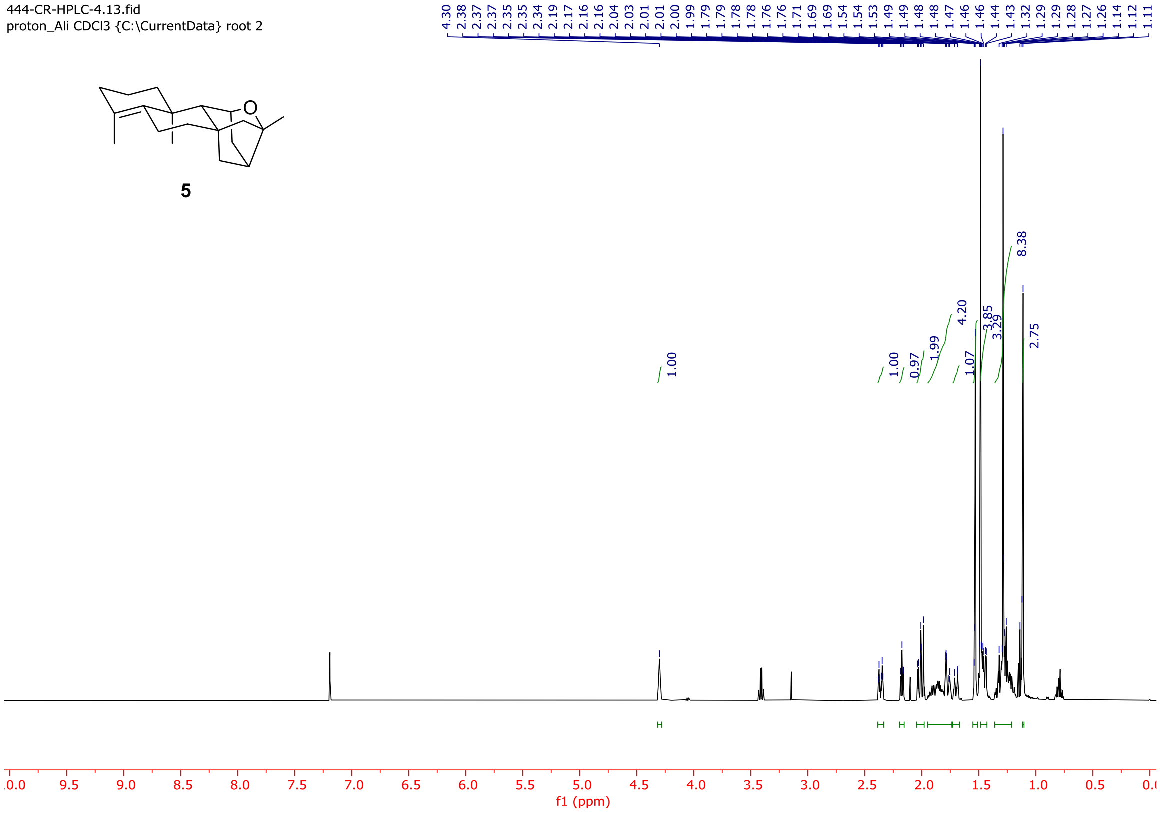

^{13}C{^{1}H} NMR of **5** (125 MHz, $CDCl_3$)

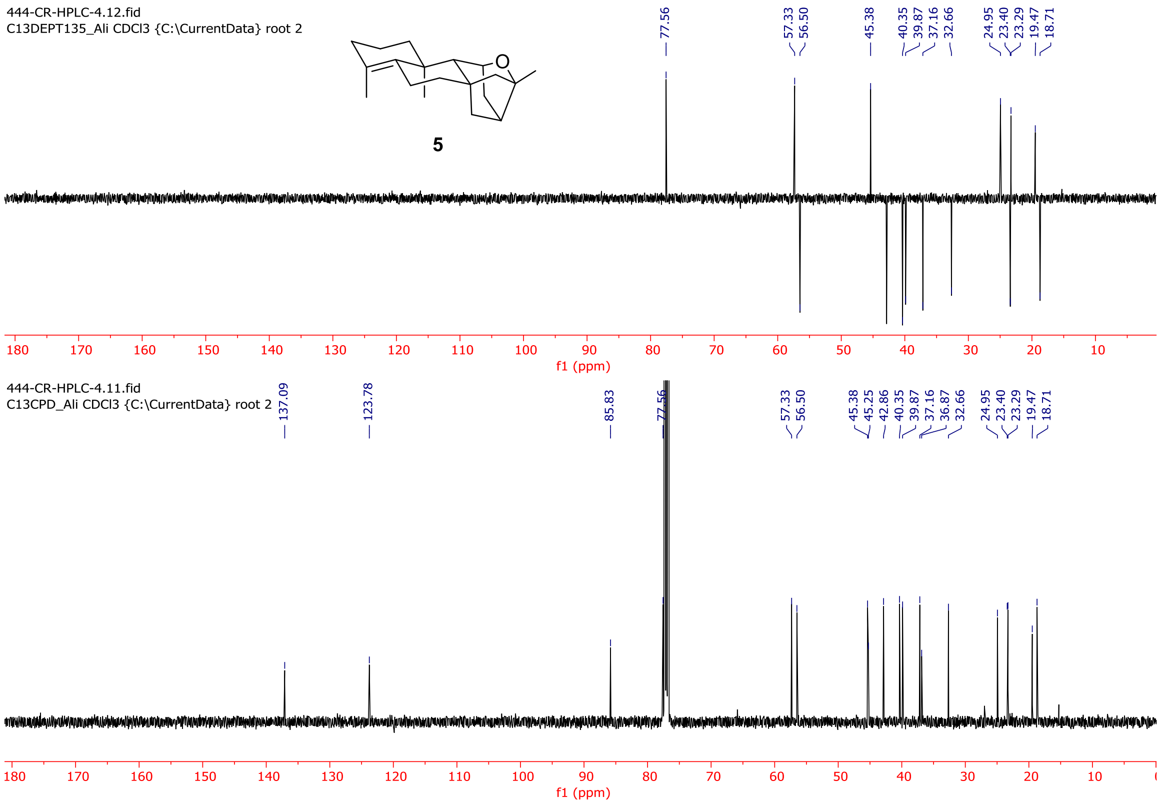

HSQC of 5 (500/125 MHz, $CDCl_3$)

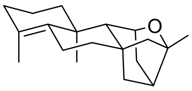

5

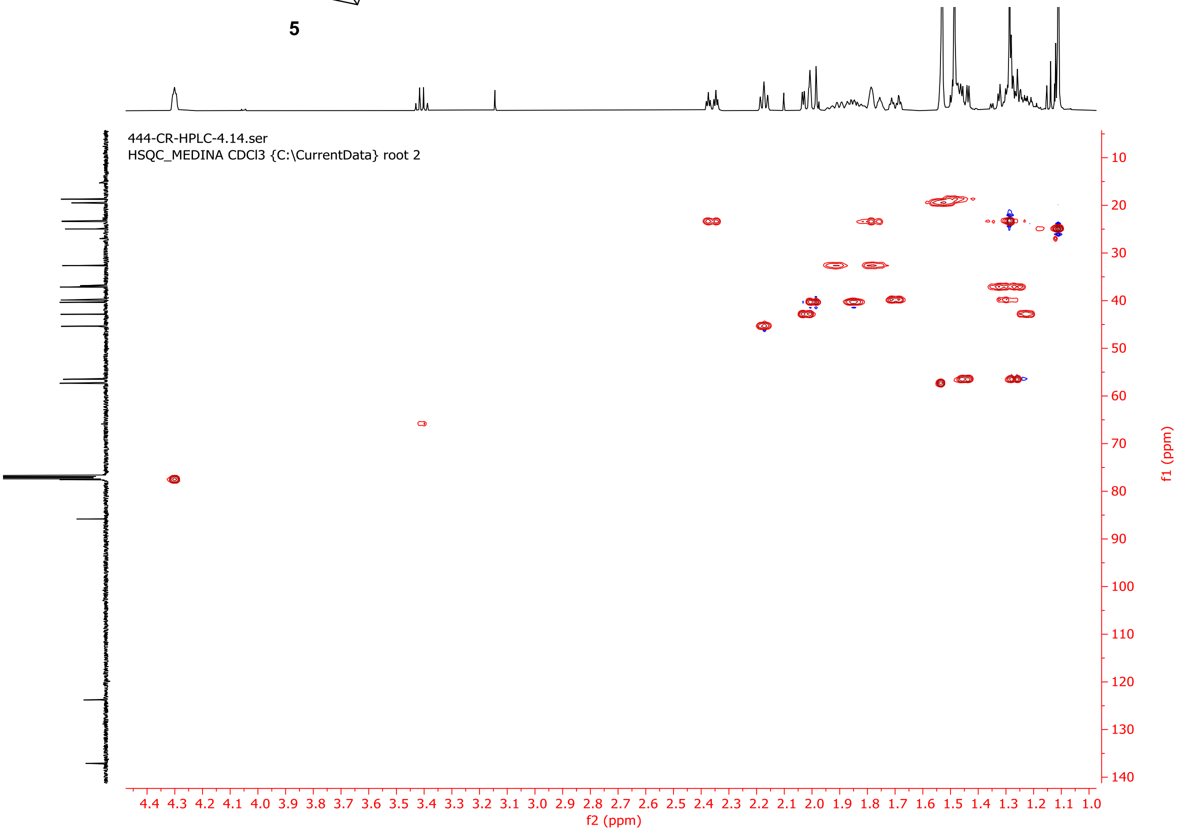

^{1}H NMR of **26** (400 MHz, $CDCl_3$)

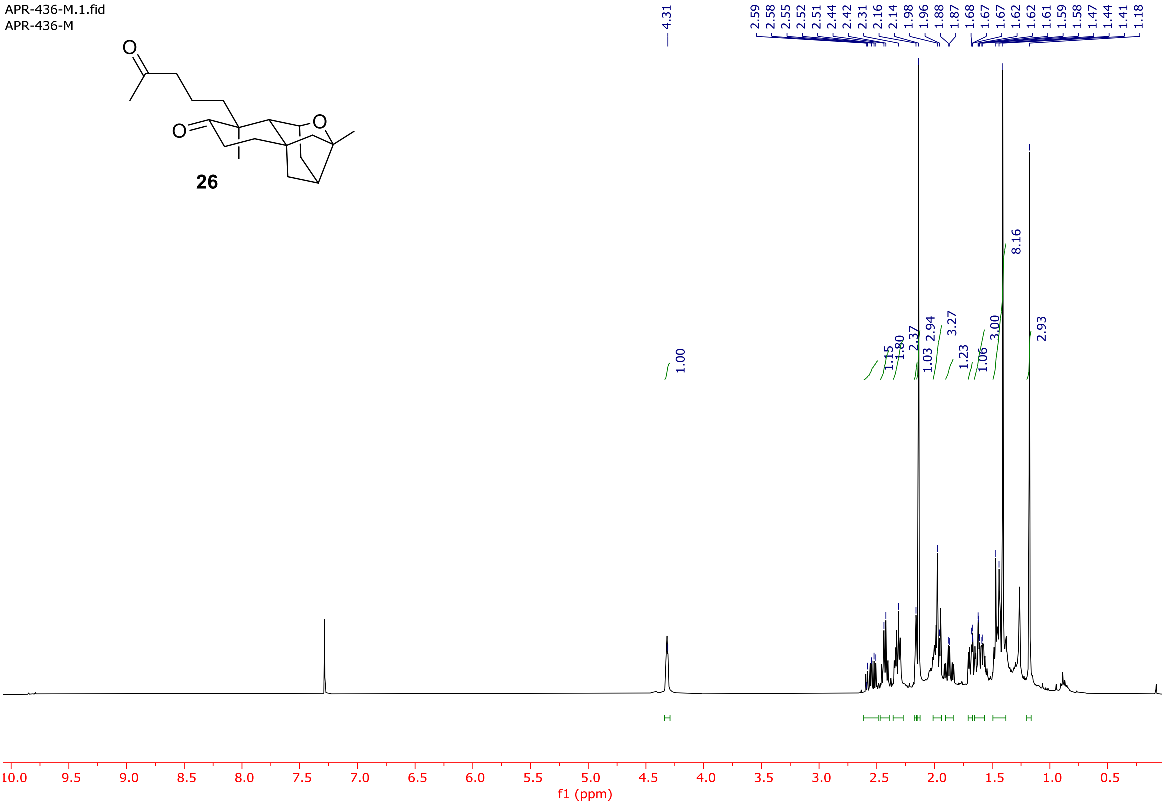

$^{13}C\{^{1}H\}$ NMR of **26** (100 MHz, $CDCl_3$)

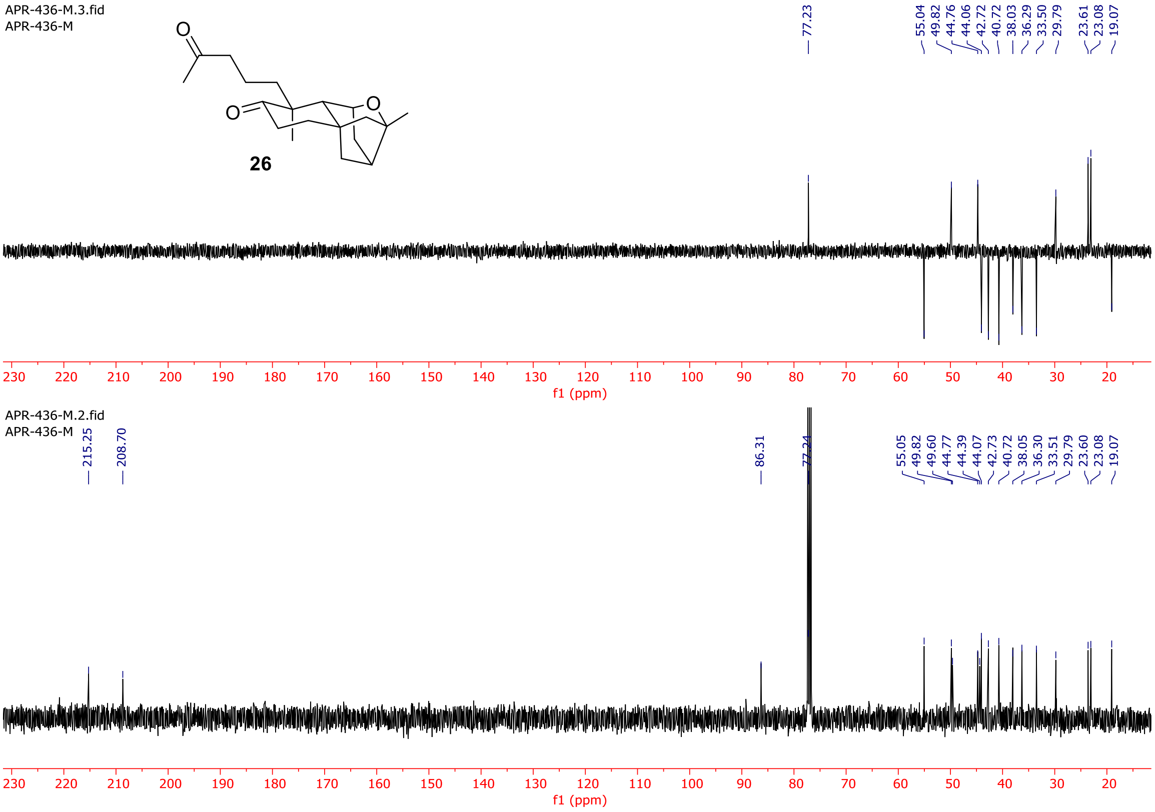

HSQC of 26 (400/100 MHz, $CDCl_3$)

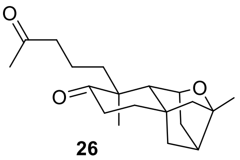

26

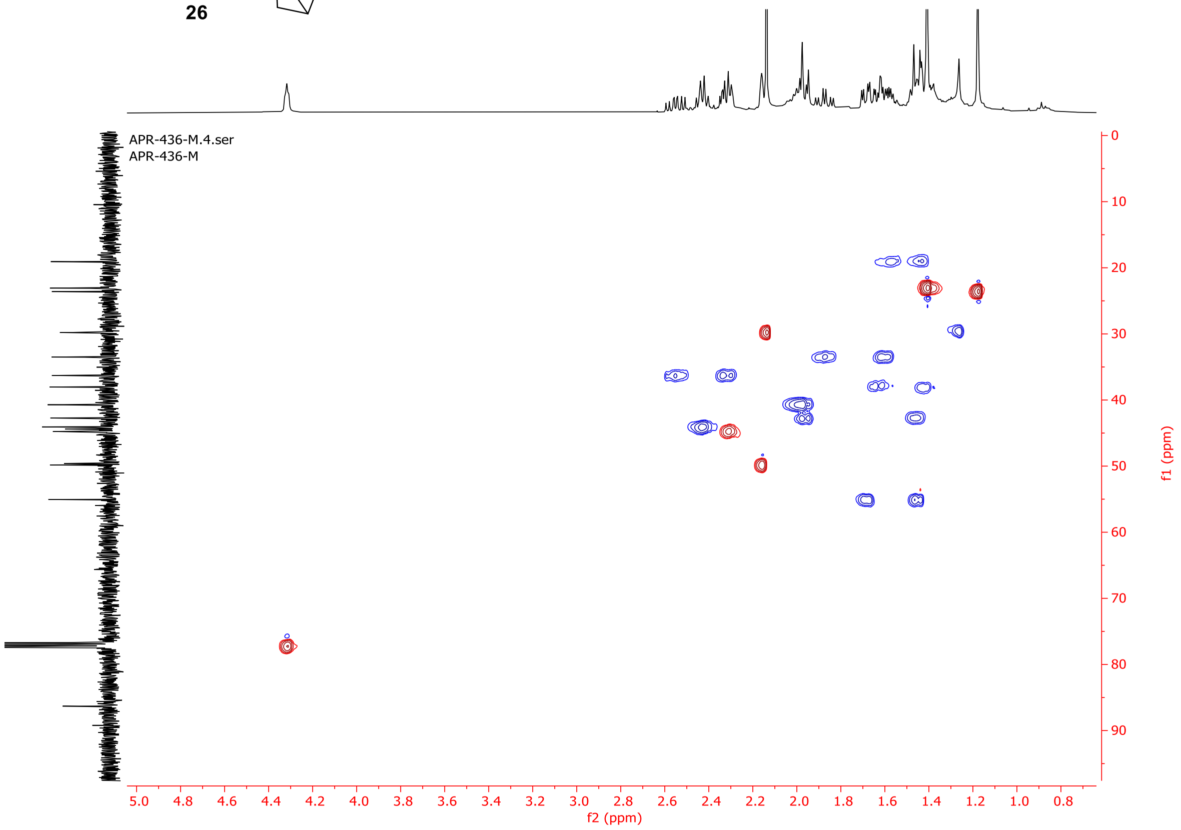

^{1}H NMR of **27** (500 MHz, $CDCl_3$)

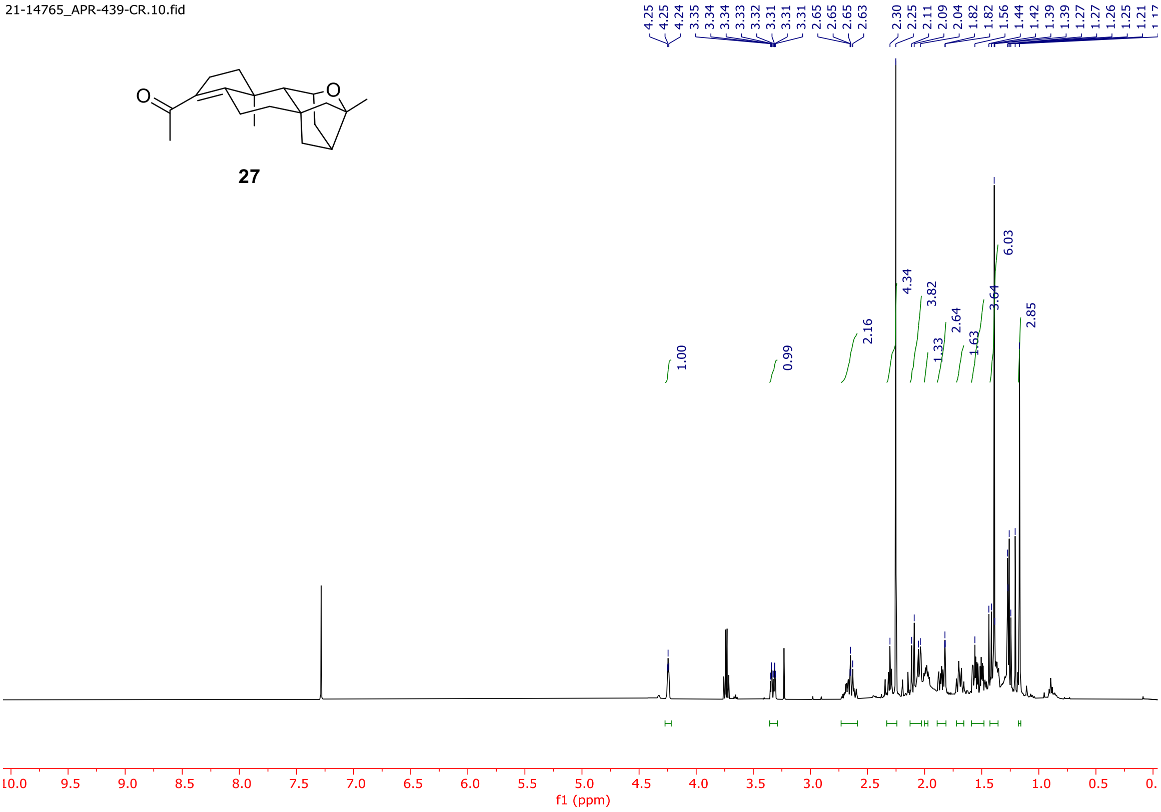

^{13}C{^{1}H} NMR of **27** (125 MHz, $CDCl_3$)

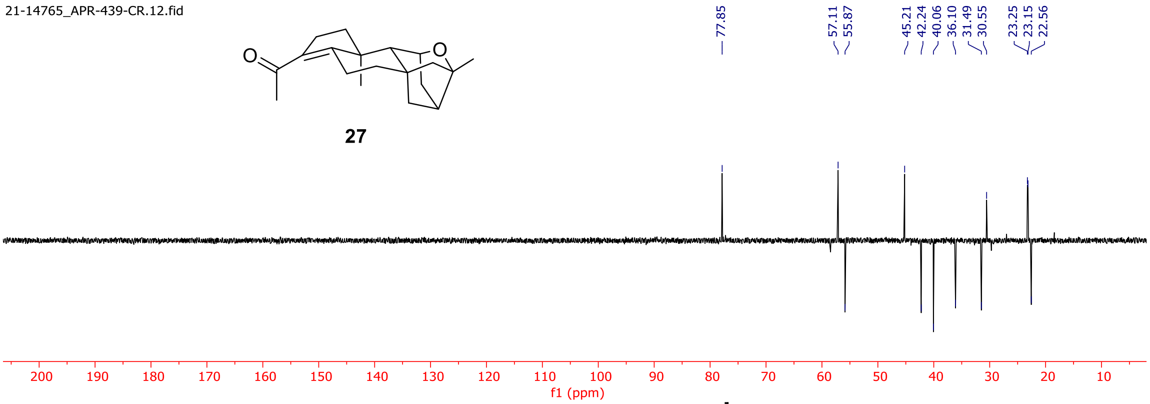

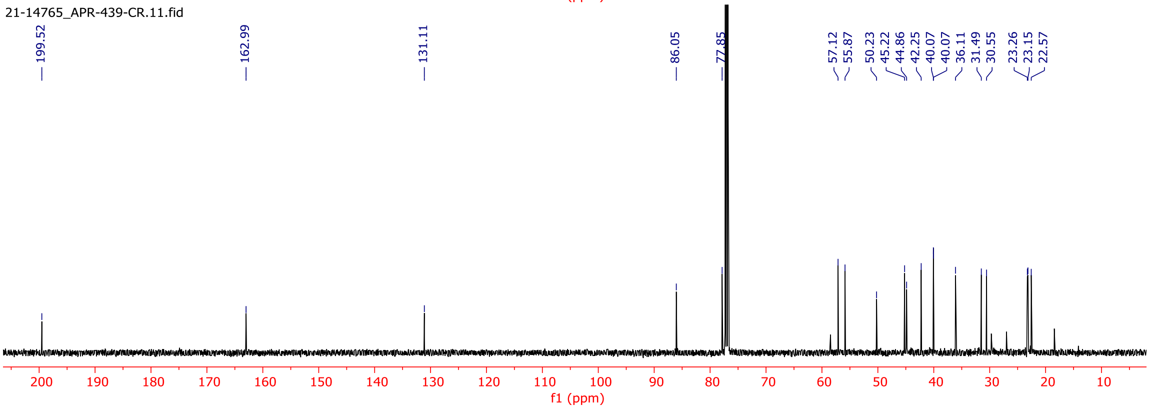

HSQC of 27 (500/125 MHz, $CDCl_3$)

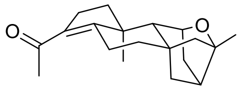

27

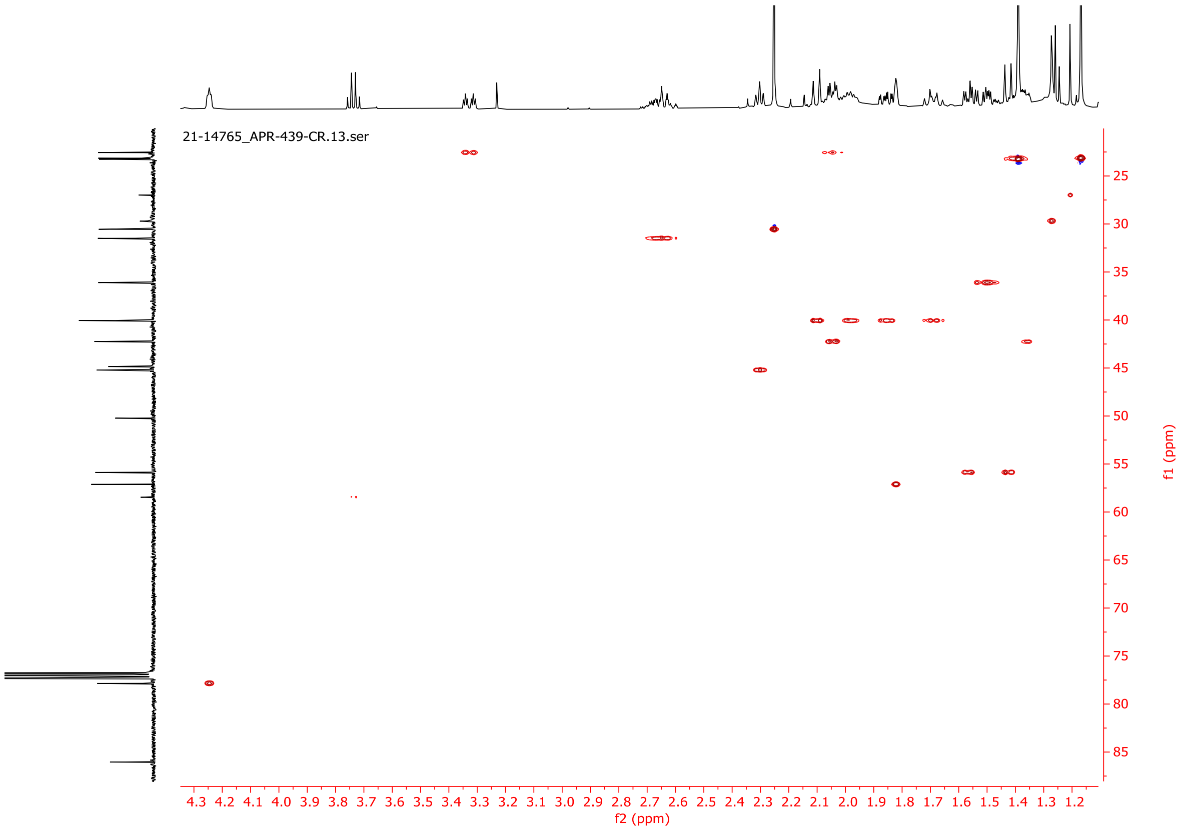

^{1}H NMR of **28** (400 MHz, $CDCl_3$)

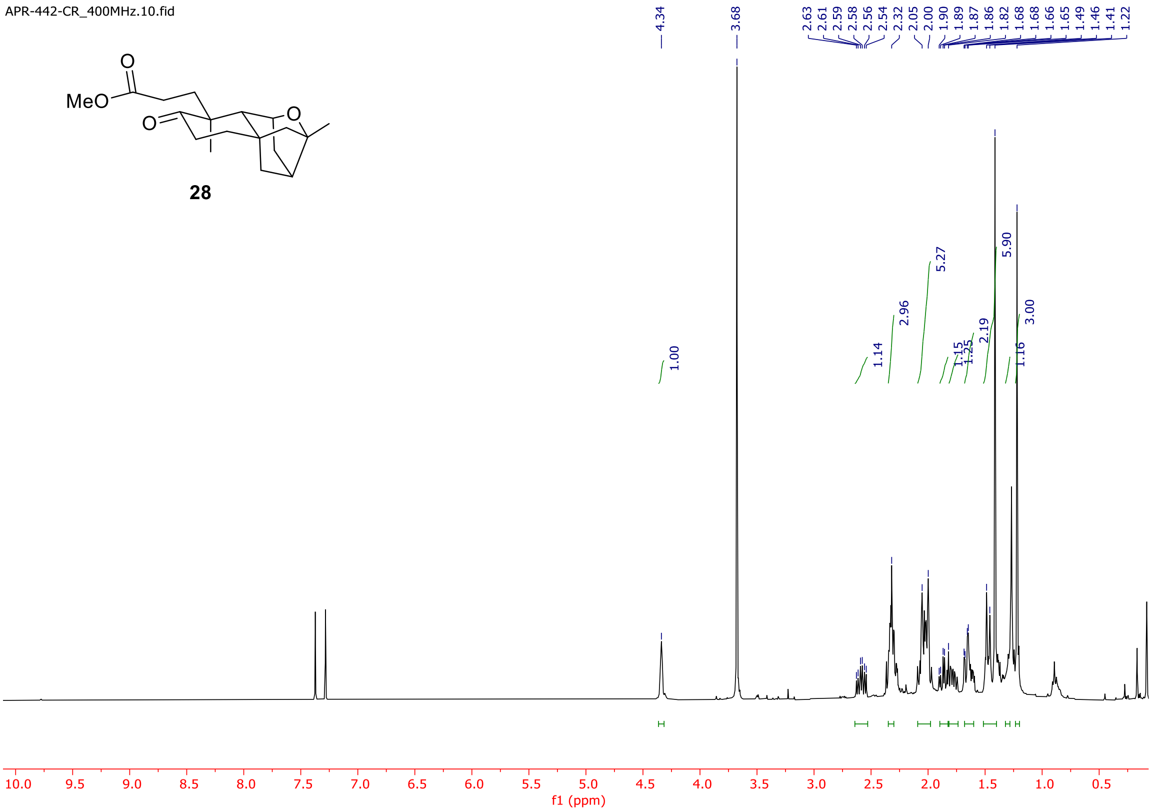

^{13}C{^{1}H} NMR of **28** (100 MHz, $CDCl_3$)

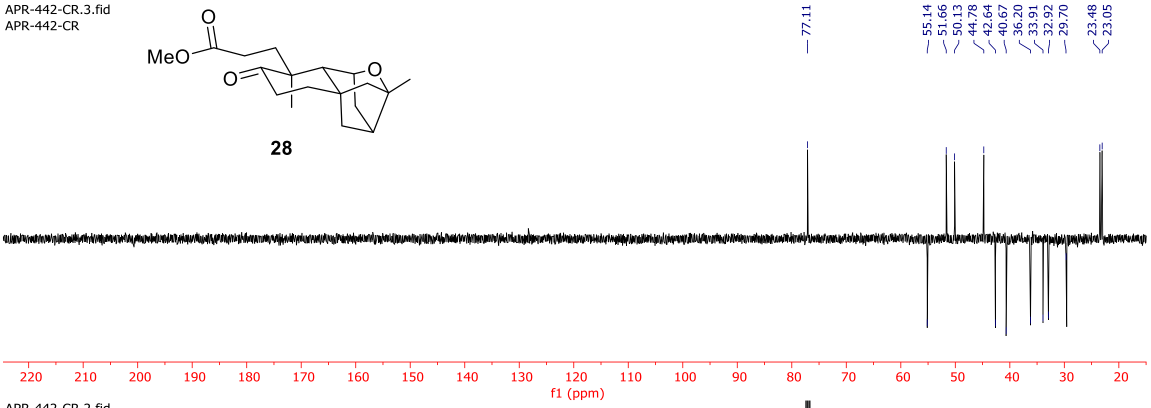

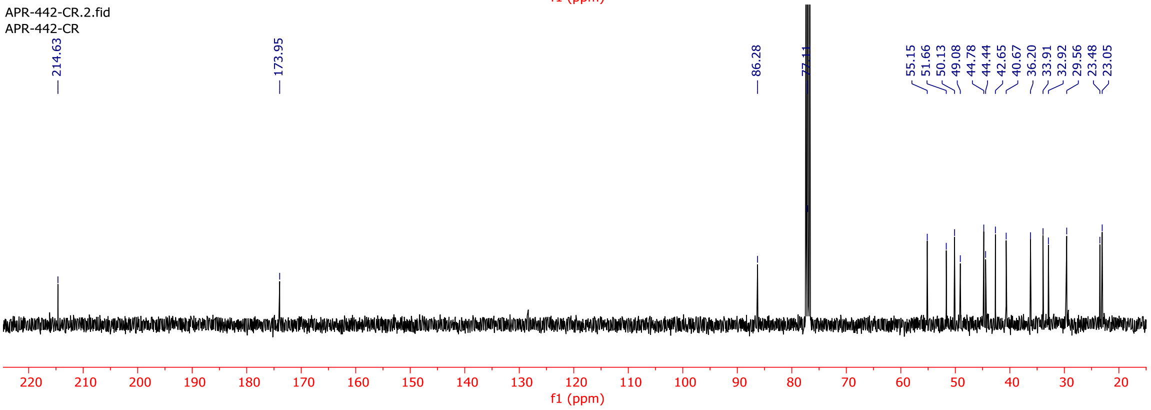

HSQC of 28 (400/100 MHz, $CDCl_3$)

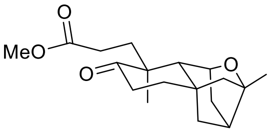

28

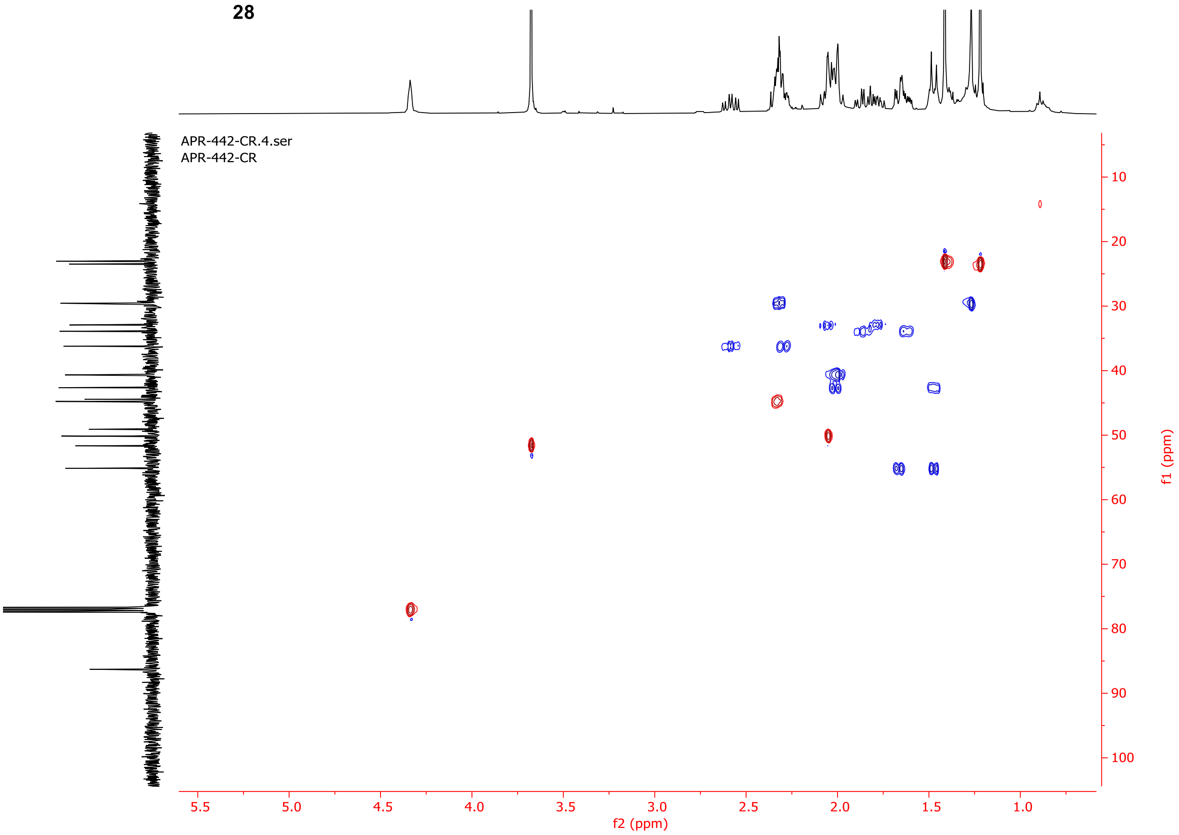

^{1}H NMR of **4a** (400 MHz, $CDCl_3$)

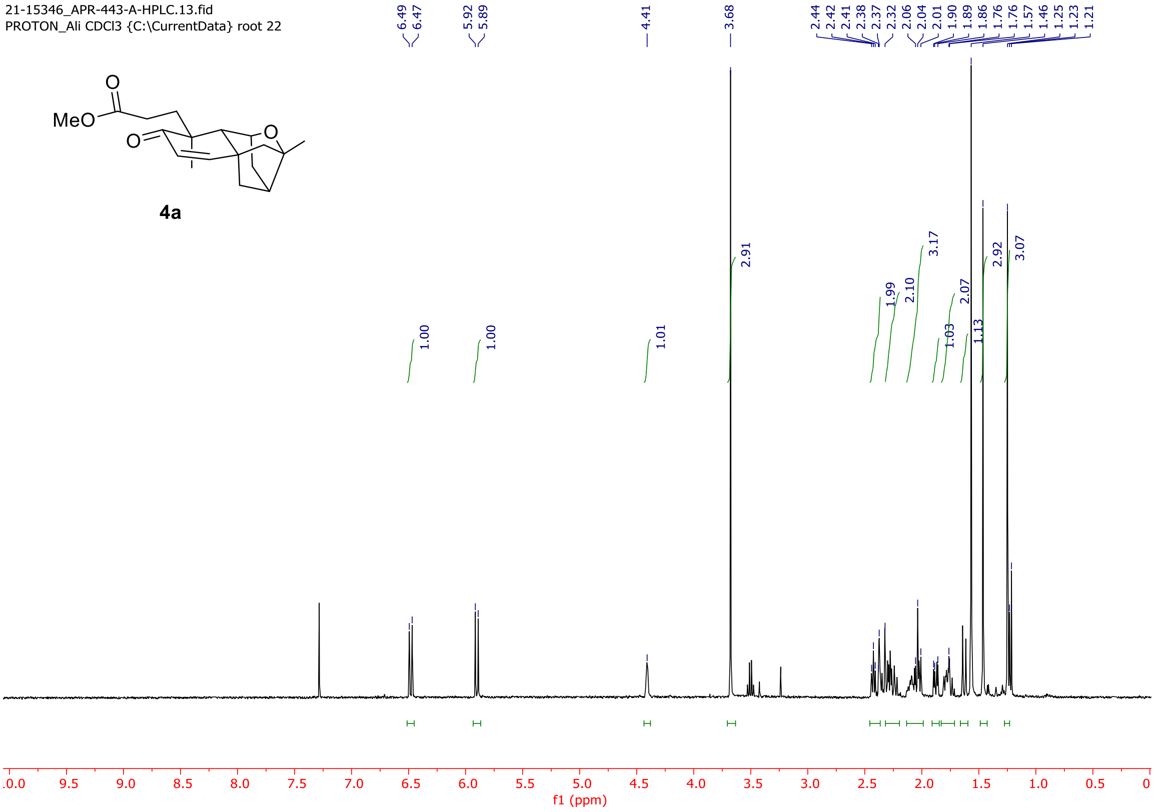

^{13}C{^{1}H} NMR of **4a** (100 MHz, $CDCl_3$)

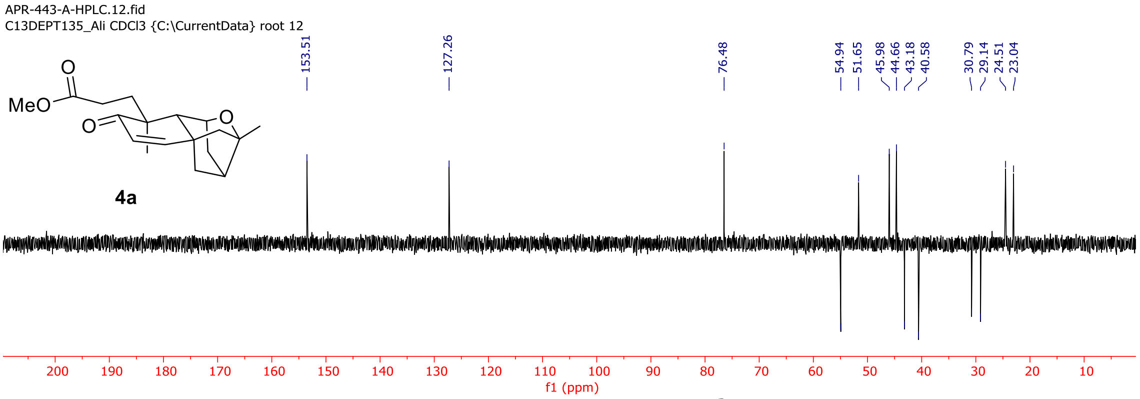

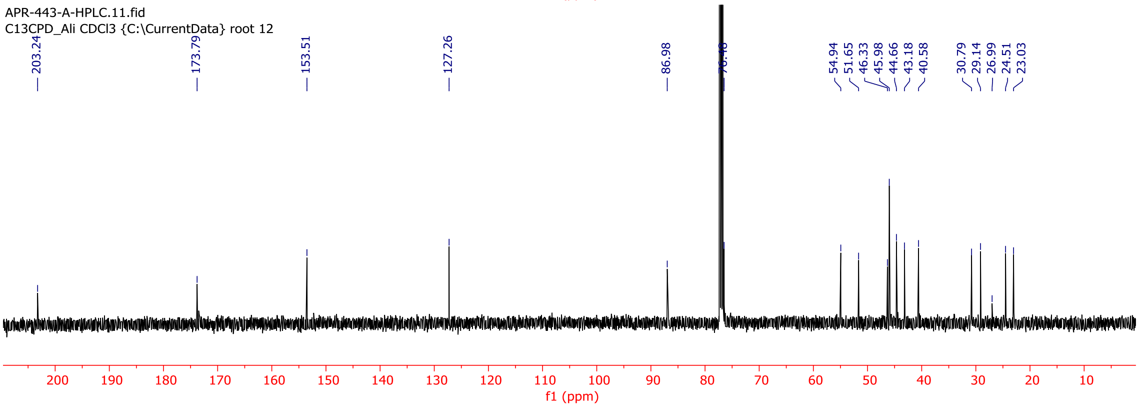

HSQC of 4a (400/100 MHz, $CDCl_3$)

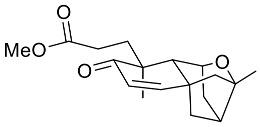

4a

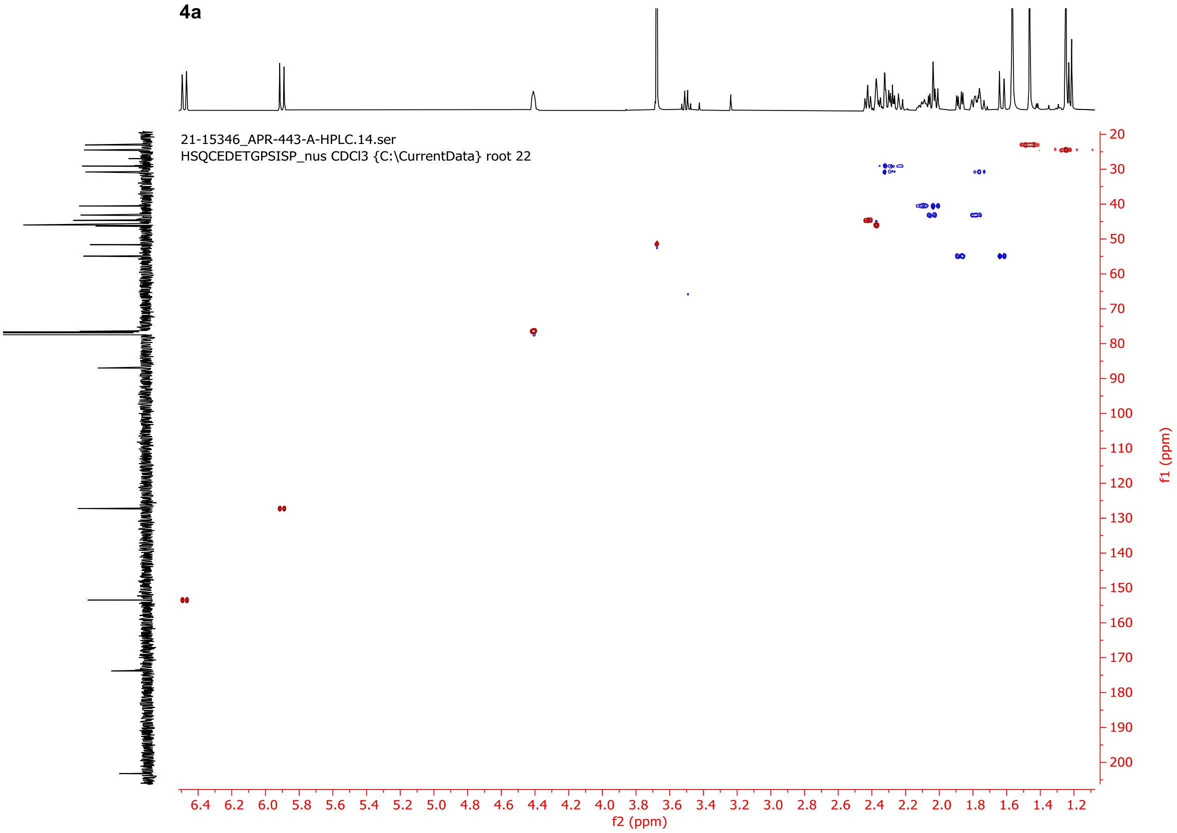

Supplement: Supplementary file 1 — ol3c01470_si_001.pdf [file ol3c01470_si_001.pdf]
