# Supplementary figures and images for: Bioinspired Synthesis of Platensimycin from Natural ent-Kaurenoic Acids
Source: Org Lett. 2023 Jun 20;25(29):5401–5. doi: 10.1021/acs.orglett.3c01470 (PMC10391625; doi:10.1021/acs.orglett.3c01470)

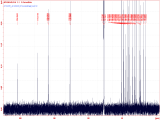

Supplement: Supplementary file 2 — ol3c01470_si_002.zip [file ol3c01470_si_002.zip › 13/13C/pdata/1/thumb.png]
