# Supplementary material for: Bioinspired Synthesis of Platensimycin from Natural ent-Kaurenoic Acids
Source: Org Lett. 2023 Jun 20;25(29):5401–5. doi: 10.1021/acs.orglett.3c01470 (PMC10391625; doi:10.1021/acs.orglett.3c01470)
Supplement: Supplementary file 2 — ol3c01470_si_002.zip [file ol3c01470_si_002.zip › 14/13C/pdata/1/iconweb.pdf]

C13CPD_Ali CDCl3 {C:\CurrentData} root 21

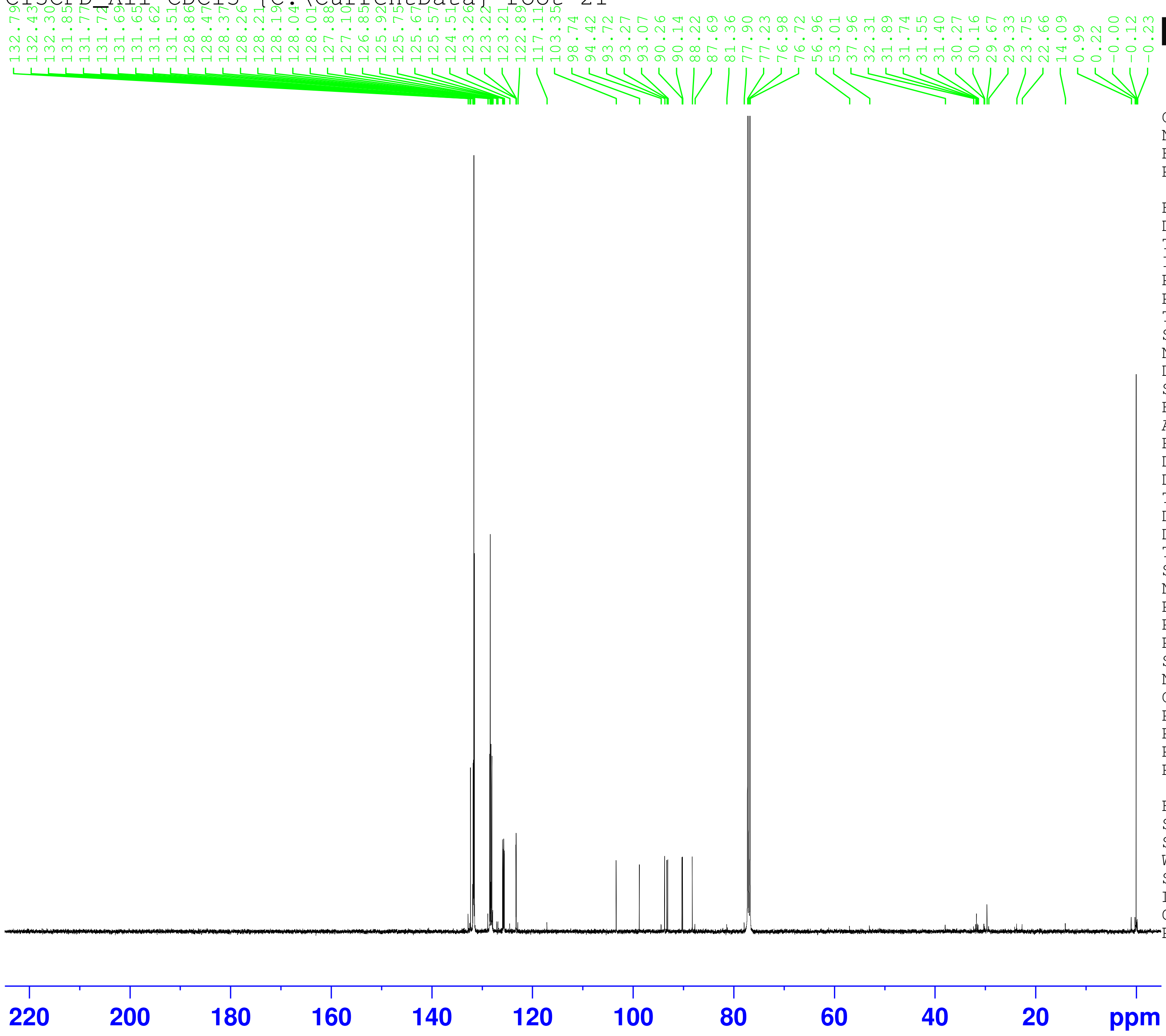

BRUKER

Current Data Parameters
NAME AOR6-95
EXPNO 11
PROCNO 1

F2 - Acquisition Parameters
Date_ 20210407
Time 23.07 h
INSTRUM AvanceNeo500
PROBHD Z119470_0013 (
PULPROG zgpg30
TD 65536
SOLVENT CDCl3
NS 4772
DS 2
SWH 28901.734 Hz
FIDRES 0.882011 Hz
AQ 1.1337729 sec
RG 101
DW 17.300 usec
DE 6.50 usec
TE 298.0 K
D1 2.00000000 sec
D11 0.03000000 sec
TD0 1
SFO1 125.7716219 MHz
NUC1 13C
P0 3.33 usec
P1 10.00 usec
PLW1 108.76999664 W
SFO2 500.1320005 MHz
NUC2 1H
CPDPRG[2 waltz16
PCPD2 80.00 usec
PLW2 14.96700001 W
PLW12 0.33676001 W
PLW13 0.16938999 W

F2 - Processing parameters
SI 262144
SF 125.7577954 MHz
WDW EM
SSB 0
LB 1.00 Hz
GB 0
PC 1.40
