# Supplementary material for: Bioinspired Synthesis of Platensimycin from Natural ent-Kaurenoic Acids
Source: Org Lett. 2023 Jun 20;25(29):5401–5. doi: 10.1021/acs.orglett.3c01470 (PMC10391625; doi:10.1021/acs.orglett.3c01470)
Supplement: Supplementary file 2 — ol3c01470_si_002.zip [file ol3c01470_si_002.zip › 2/13C/pdata/1/iconweb.pdf]

C13CPD_ali CDCl3 {C:\CurrentData} root 4

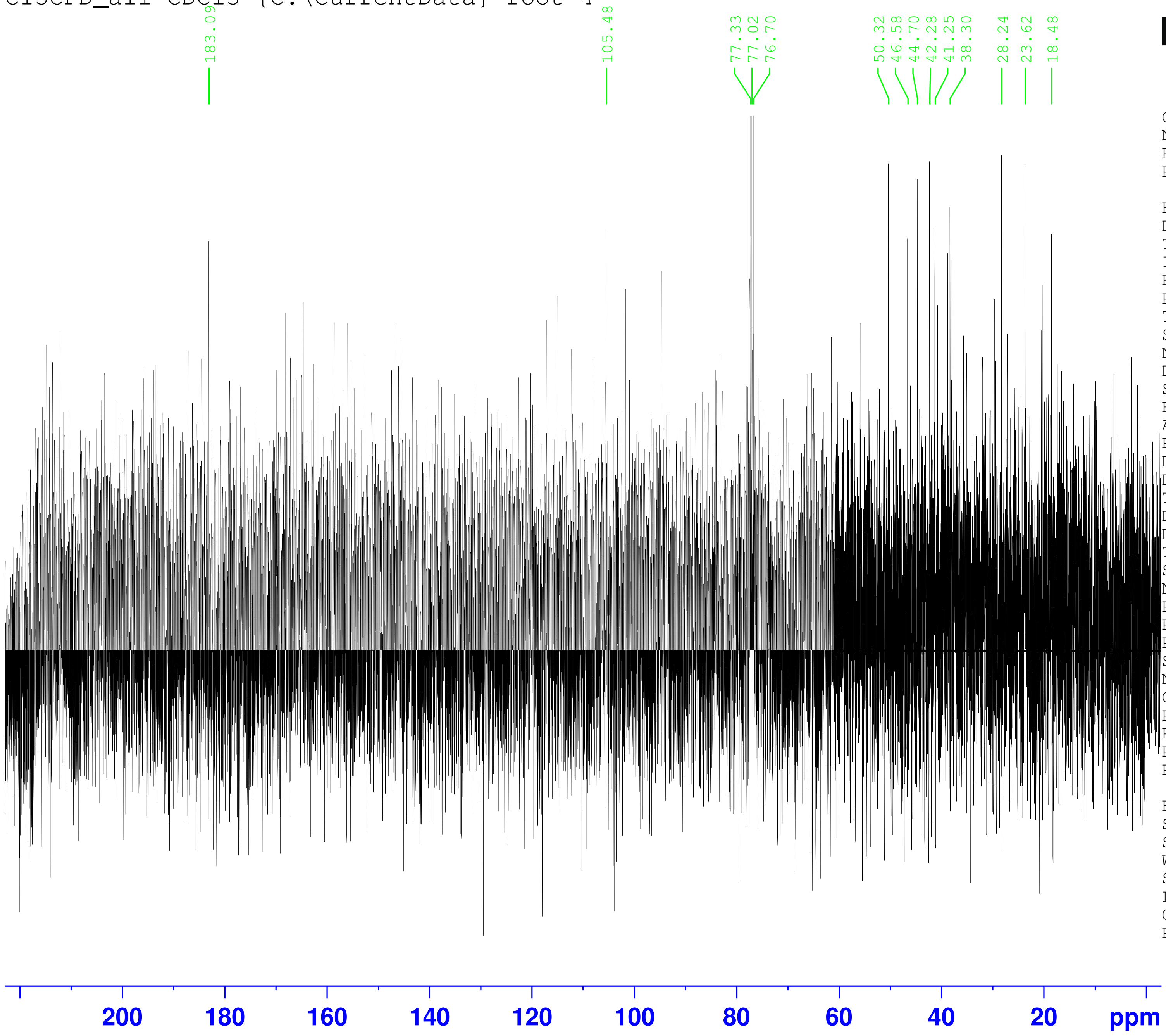

BRUKER

Current Data Parameters
NAME APR-9
EXPNO 11
PROCNO 1

F2 - Acquisition Parameters
Date_ 20210909
Time 13.24 h
INSTRUM Avance NEO
PROBHD Z163739_0001 (
PULPROG zgpg30
TD 65536
SOLVENT CDCl3
NS 22
DS 4
SWH 22727.273 Hz
FIDRES 0.693581 Hz
AQ 1.4417920 sec
RG 101
DW 22.000 usec
DE 10.00 usec
TE 299.8 K
D1 2.00000000 sec
D11 0.03000000 sec
TD0 1
SFO1 100.6313803 MHz
NUC1 13C
P0 3.33 usec
P1 10.00 usec
PLW1 60.21799850 W
SFO2 400.1616006 MHz
NUC2 1H
CPDPRG[2 waltz65
PCPD2 90.00 usec
PLW2 15.66699982 W
PLW12 0.19341999 W
PLW13 0.09728900 W

F2 - Processing parameters
SI 262144
SF 100.6203120 MHz
WDW EM
SSB 0
LB 1.00 Hz
GB 0
PC 1.40
