# Supplementary material for: Bioinspired Synthesis of Platensimycin from Natural ent-Kaurenoic Acids
Source: Org Lett. 2023 Jun 20;25(29):5401–5. doi: 10.1021/acs.orglett.3c01470 (PMC10391625; doi:10.1021/acs.orglett.3c01470)
Supplement: Supplementary file 2 — ol3c01470_si_002.zip [file ol3c01470_si_002.zip › 3a/13C/pdata/1/iconweb.pdf]

C13CPD_Ali CDCl3 {C:\CurrentData} root 19

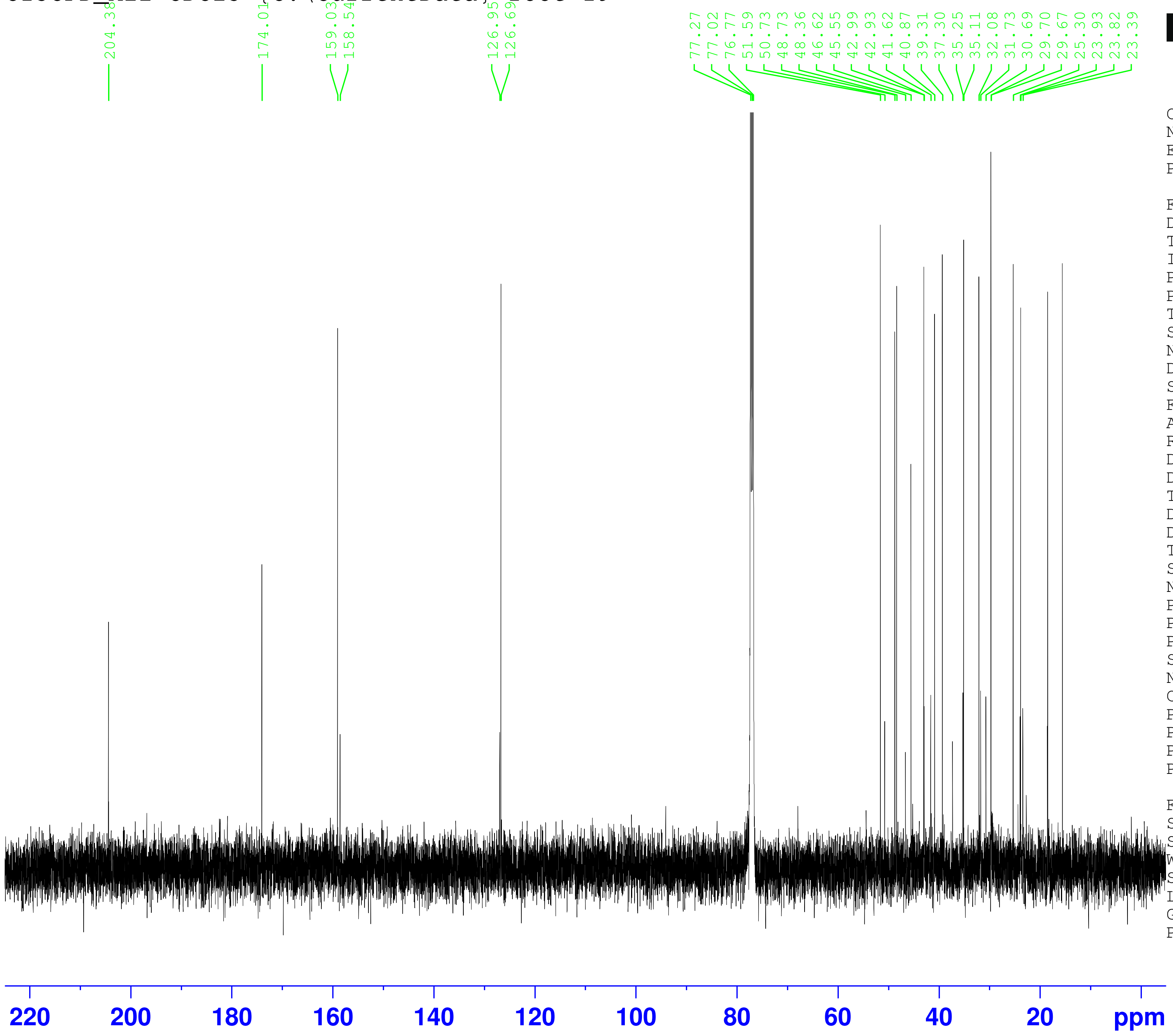

BRUKER

Current Data Parameters

NAME	APR-290-HPLC-M	
EXPNO	11	
PROCNO	1	

F2 - Acquisition Parameters

Date_	20210908	
Time	19.21	h
INSTRUM	AvanceNeo500	
PROBHD	Z119470_0013 (	
PULPROG	zgpg30	
TD	65536	
SOLVENT	CDCl3	
NS	8000	
DS	2	
SWH	28901.734	Hz
FIDRES	0.882011	Hz
AQ	1.1337729	sec
RG	101	
DW	17.300	usec
DE	6.50	usec
TE	298.2	K
D1	2.00000000	sec
D11	0.03000000	sec
TD0	1	
SFO1	125.7716219	MHz
NUC1	13C	
P0	3.33	usec
P1	10.00	usec
PLW1	108.76999664	W
SFO2	500.1320005	MHz
NUC2	1H	
CPDPRG[2	waltz16	
PCPD2	80.00	usec
PLW2	14.96700001	W
PLW12	0.33676001	W
PLW13	0.16938999	W

F2 - Processing parameters

SI	262144	
SF	125.7577885	MHz
WDW	EM	
SSB	0	
LB	1.00	Hz
GB	0	
PC	1.40	
