# Supplementary material for: Bioinspired Synthesis of Platensimycin from Natural ent-Kaurenoic Acids
Source: Org Lett. 2023 Jun 20;25(29):5401–5. doi: 10.1021/acs.orglett.3c01470 (PMC10391625; doi:10.1021/acs.orglett.3c01470)
Supplement: Supplementary file 2 — ol3c01470_si_002.zip [file ol3c01470_si_002.zip › 3a/1H/pdata/1/iconweb.pdf]

Barrero
proton_Ali CDCl3 {C:\CurrentData} root 19

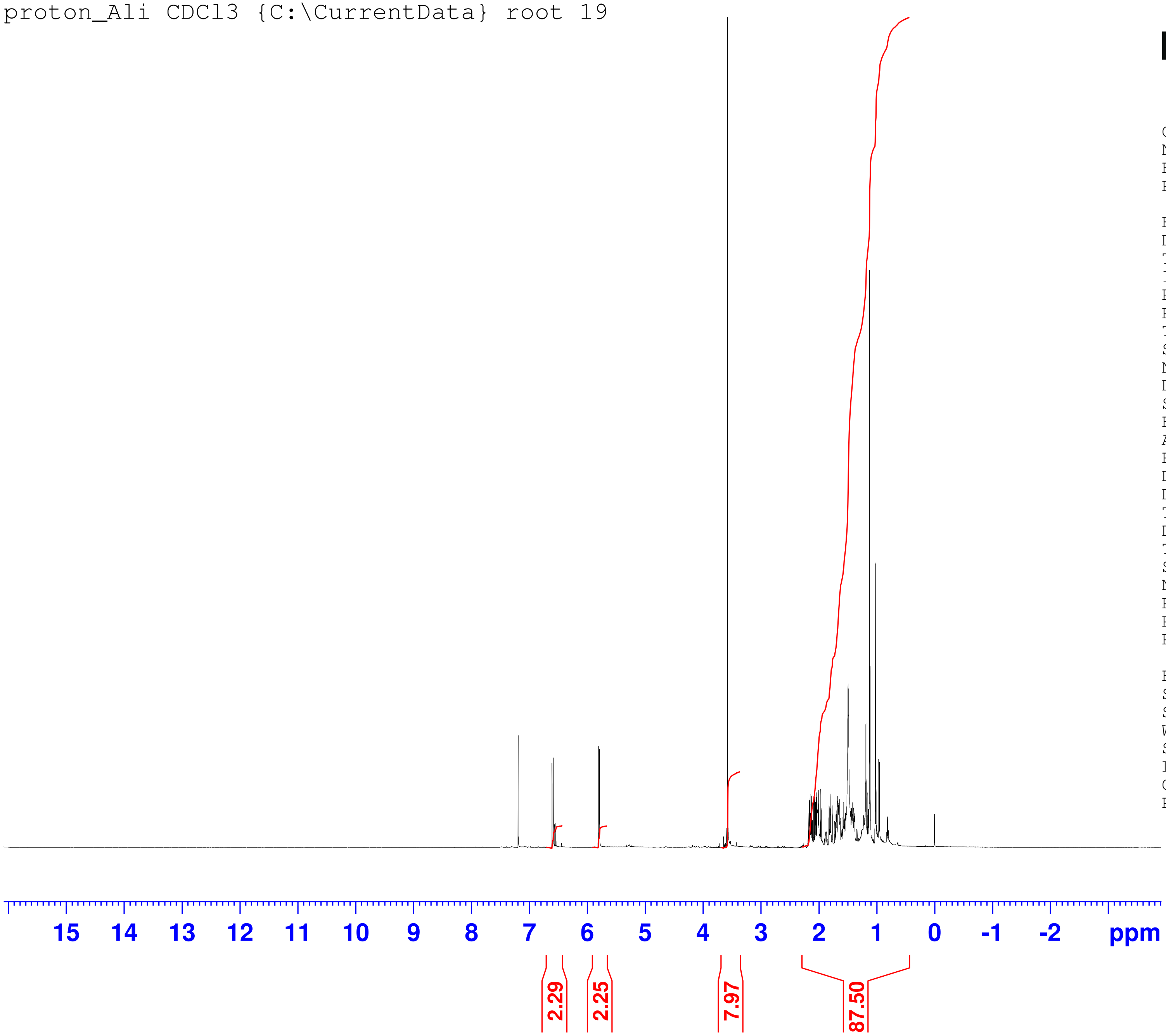

BRUKER

Current Data Parameters

NAME	APR-290-HPLC-M
EXPNO	10
PROCNO	1

F2 - Acquisition Parameters

Date_	20210908
Time	12.15 h
INSTRUM	AvanceNeo500
PROBHD	Z119470_0013 (
PULPROG	zg30
TD	65536
SOLVENT	CDCl3
NS	128
DS	2
SWH	10000.000 Hz
FIDRES	0.305176 Hz
AQ	3.2767999 sec
RG	101
DW	50.000 usec
DE	10.45 usec
TE	298.1 K
D1	1.00000000 sec
TD0	1
SFO1	500.1330883 MHz
NUC1	1H
P0	4.00 usec
P1	12.00 usec
PLW1	14.96700001 W

F2 - Processing parameters

SI	65536
SF	500.1300460 MHz
WDW	EM
SSB	0
LB	0.30 Hz
GB	0
PC	1.00
