# Supplementary material for: Bioinspired Synthesis of Platensimycin from Natural ent-Kaurenoic Acids
Source: Org Lett. 2023 Jun 20;25(29):5401–5. doi: 10.1021/acs.orglett.3c01470 (PMC10391625; doi:10.1021/acs.orglett.3c01470)
Supplement: Supplementary file 2 — ol3c01470_si_002.zip [file ol3c01470_si_002.zip › 3a/DEPT/pdata/1/iconweb.pdf]

C13DEPT135_Ali CDCl3 {C:\CurrentData} root 19

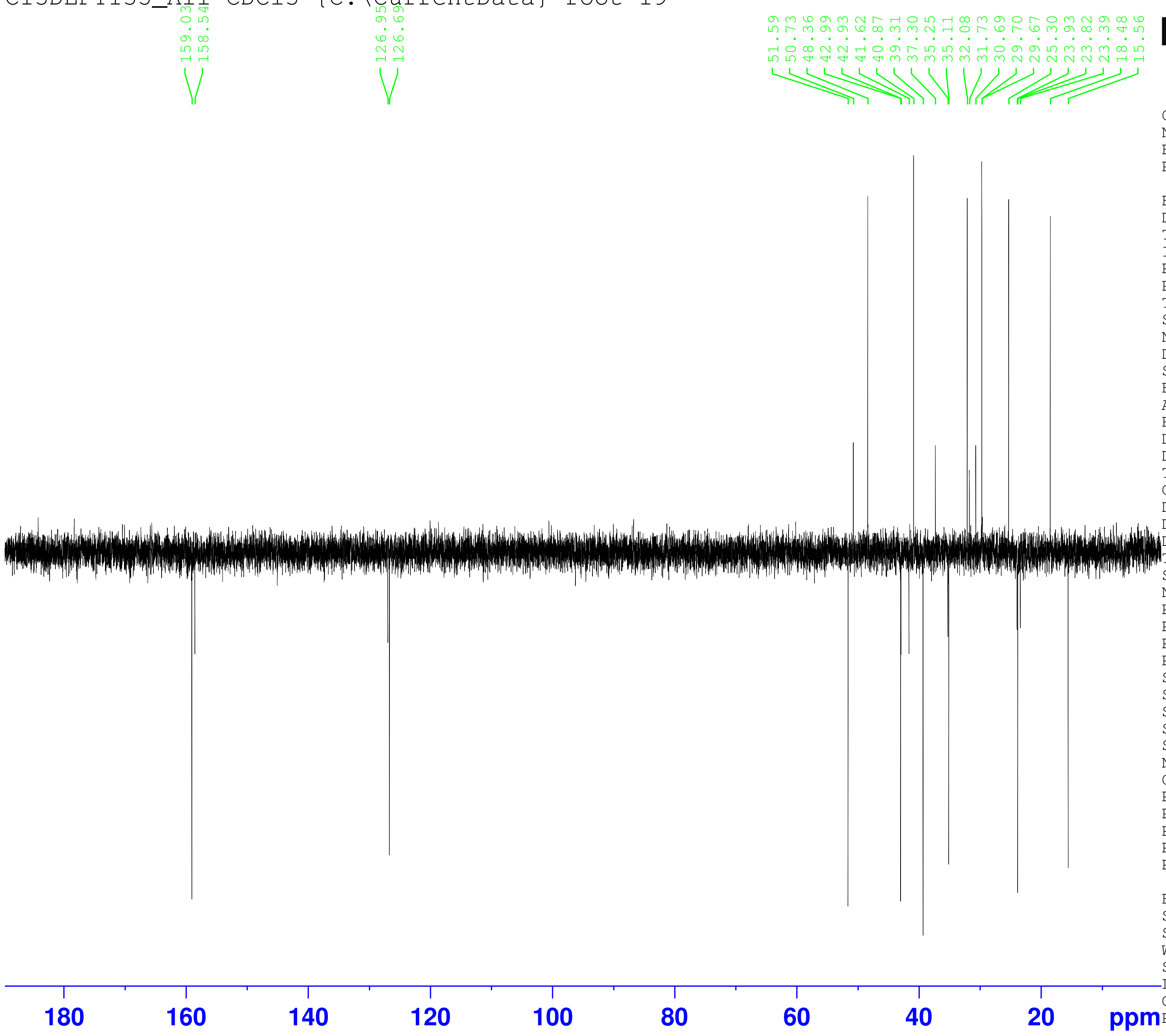

Current Data Parameters

NAME	APR-290-HPLC-M
EXPNO	12
PROCNO	1

F2 - Acquisition Parameters

Date_	20210908
Time	23.01 h
INSTRUM	AvanceNeo500
PROBHD	Z119470_0013 (
PULPROG	deptsp135
TD	59392
SOLVENT	CDCl3
NS	4000
DS	8
SWH	23809.523 Hz
FIDRES	0.801775 Hz
AQ	1.2472320 sec
RG	101
DW	21.000 usec
DE	6.50 usec
TE	298.1 K
CNST2	145.0000000
D1	2.00000000 sec
D2	0.00344828 sec
D12	0.00002000 sec
TD0	1
SFO1	125.7697355 MHz
NUC1	13C
P1	10.00 usec
P13	2000.00 usec
PLW0	0 W
PLW1	108.76999664 W
SPNAM[5]	Crp60comp.4
SPOAL5	0.500
SPOFFS5	0 Hz
SPW5	16.61899948 W
SFO2	500.1320005 MHz
NUC2	1H
CPDPRG[2	waltz16
P3	12.00 usec
P4	24.00 usec
PCPD2	80.00 usec
PLW2	14.96700001 W
PLW12	0.33676001 W

F2 - Processing parameters

SI	262144
SF	125.7577885 MHz
WDW	EM
SSB	0
LB	1.00 Hz
GB	0
PC	1.40
