# Supplementary material for: Bioinspired Synthesis of Platensimycin from Natural ent-Kaurenoic Acids
Source: Org Lett. 2023 Jun 20;25(29):5401–5. doi: 10.1021/acs.orglett.3c01470 (PMC10391625; doi:10.1021/acs.orglett.3c01470)
Supplement: Supplementary file 2 — ol3c01470_si_002.zip [file ol3c01470_si_002.zip › 3a/HSQC/pdata/1/iconweb.pdf]

HSQC_MEDINA CDCl3 {C:\CurrentData} root 19

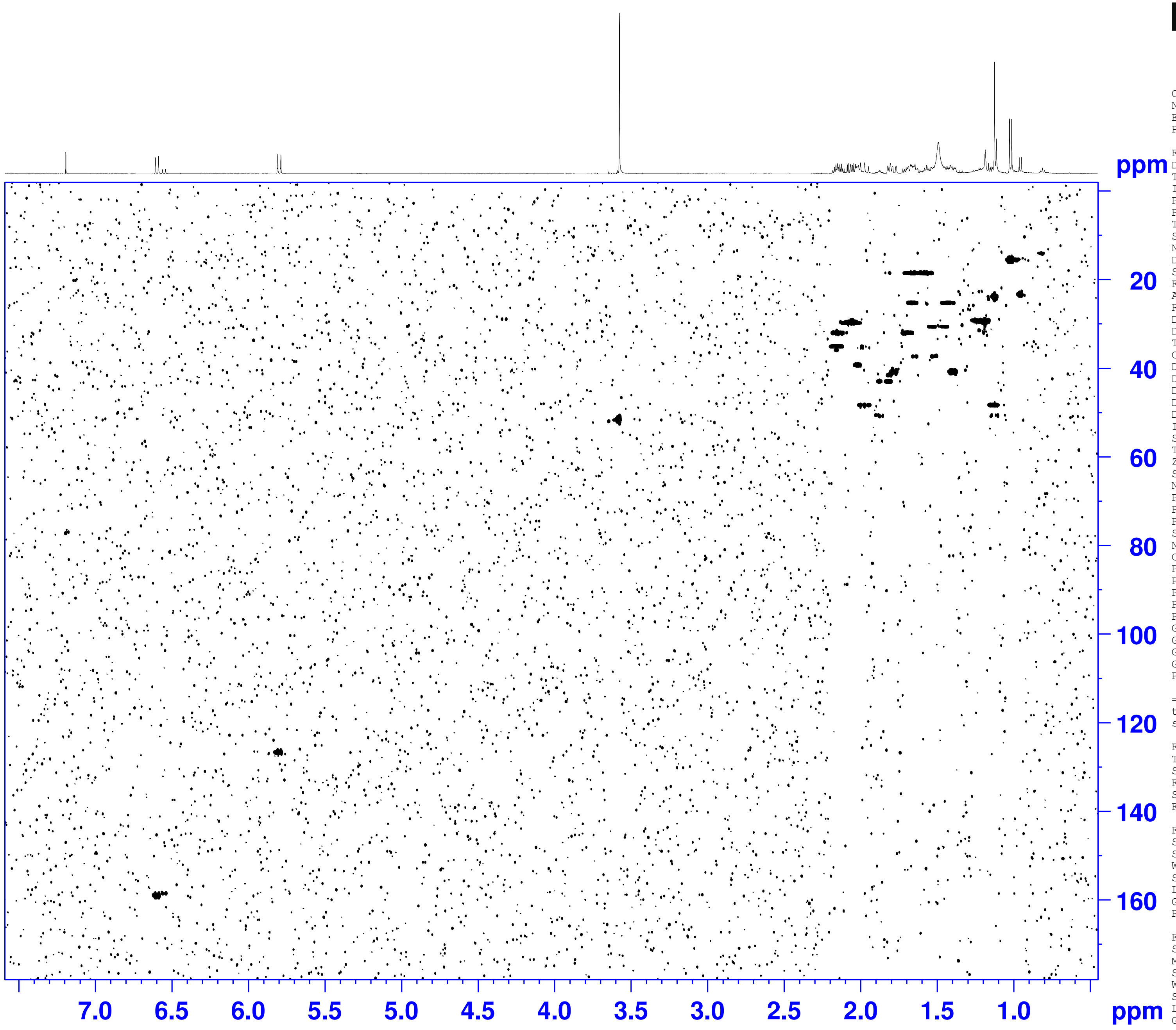


```
Current Data Parameters
NAME      APR-290-HPLC-M
EXPNO                 14
PROCNO                 1

F2 - Acquisition Parameters
Date_           20210909
Time                2.11 h
INSTRUM      AvanceNeo500
PROBHD     Z119470_0013 (
PULPROG         hsqcetgp
TD                  2048
SOLVENT            CDCl3
NS                   120
DS                    16
SWH             3571.428 Hz
FIDRES          3.487723 Hz
AQ             0.2867200 sec
RG                   101
DW               140.000 usec
DE                 17.50 usec
TE                 298.1 K
CNST2        145.0000000
D0            0.00000300 sec
D1            1.50000000 sec
D4            0.00172414 sec
D11           0.03000000 sec
D16           0.00020000 sec
IN0           0.00002209 sec
SPARSELI               0
TDav                   1
ZGOPTNS
SFO1         500.1320567 MHz
NUC1                  1H
P1                 12.00 usec
P2                 24.00 usec
PLW1         14.96700001 W
SFO2         125.7688552 MHz
NUC2                 13C
CPDPRG[2           garp4
P3                 10.00 usec
P4                 20.00 usec
PCPD2              70.00 usec
PLW2        108.76999664 W
PLW12         2.21980000 W
GPNAM[1]      SMSQ10.100
GPZ1               80.00 %
GPNAM[2]      SMSQ10.100
GPZ2               20.10 %
P16              1000.00 usec

======== F1 INDIRECT DIMENSION ========
td1       512
sw_F1     180.003967

F1 - Acquisition parameters
TD                    52
SFO1            125.7689 MHz
FIDRES        870.663025 Hz
SW               179.991 ppm
FnMODE     Echo-Antiecho

F2 - Processing parameters
SI                  8192
SF           500.1300460 MHz
WDW                QSINE
SSB                    2
LB                     0 Hz
GB                     0
PC                  1.40

F1 - Processing parameters
SI                  1024
MC2       echo-antiecho
SF           125.7577885 MHz
WDW                QSINE
SSB                    2
LB                     0 Hz
GB                     0
```
